# Key Signatures of Magnetofossils Elucidated by Mutant Magnetotactic Bacteria and Micromagnetic Calculations

**DOI:** 10.1029/2021JB023239

**Published:** 2021-12-28

**Authors:** Matthieu Amor, Juan Wan, Ramon Egli, Julie Carlut, Christophe Gatel, Ingrid Marie Andersen, Etienne Snoeck, Arash Komeili

**Affiliations:** 1 Department of Plant and Microbial Biology, University of California, Berkeley, CA, USA; 2 Aix-Marseille Université, CEA, CNRS, BIAM, Saint-Paul-lez-Durance, France; 3 Zentralanstalt für Meteorologie und Geodynamik (ZAMG), Vienna, Austria; 4 Université de Paris, Institut de Physique du Globe de Paris, CNRS, Paris, France; 5 CEMES CNRS, Toulouse, France; 6 Université de Toulouse, Toulouse, France; 7 Department of Molecular and Cell Biology, University of California, Berkeley, CA, USA

## Abstract

Magnetotactic bacteria (MTB) produce single-stranded or multi-stranded chains of magnetic nanoparticles that contribute to the magnetization of sediments and rocks. Their magnetic fingerprint can be detected in ancient geological samples and serve as a unique biosignature of microbial life. However, some fossilized assemblages bear contradictory signatures pointing to magnetic components that have distinct origin(s). Here, using micromagnetic simulations and mutant MTB producing looped magnetosome chains, we demonstrate that the observed magnetofossil fingerprints are produced by a mixture of single-stranded and multi-stranded chains, and that diagenetically induced chain collapse, if occurring, must preserve the strong uniaxial anisotropy of native chains. This anisotropy is the key factor for distinguishing magnetofossils from other populations of natural magnetite particles, including those with similar individual crystal characteristics. Furthermore, the detailed properties of magnetofossil signatures depend on the proportion of equant and elongated magnetosomes, as well as on the relative abundances of single-stranded and multi-stranded chains. This work has important paleoclimatic, paleontological, and phylogenetic implications, as it provides reference data to differentiate distinct MTB lineages according to their chain and magnetosome morphologies, which will enable the tracking of the evolution of some of the most ancient biomineralizing organisms in a time-resolved manner. It also enables a more accurate discrimination of different sources of magnetite particles, which is pivotal for gaining better environmental and relative paleointensity reconstructions from sedimentary records.

## Plain Language Summary

Magnetotactic bacteria (MTB) produce chains of magnetic nanoparticles that contribute to the magnetization of sedimentary rocks. Fossilized chains can be detected in ancient geological samples using magnetic measurements. They provide unique evidence for microbial life. However, remains of fossil MTB possess ambiguous magnetic properties that make their detection problematic. Here, we demonstrate, with the help of numerical simulations and a modified MTB producing looped chains, that the magnetic properties of MTB-rich sediments can be explained by a combination of chains made of single and multiple strands, and that chain collapse in the sediment, if occurring, likely preserves key magnetic properties, enabling the identification of fossil MTB. These properties also depend on the proportion of equidimensional and elongated magnetosomes, as well as on the relative abundances of single- and multi-stranded chains. This work has important implications, as it provides new data to differentiate distinct MTB types according to their chain and magnetosome morphologies, enabling the tracking of the evolution of some of the most ancient mineral-producing organisms. It also enables a more accurate discrimination of different sources of magnetite particles, which helps gain better environmental and relative paleointensity reconstructions from sedimentary records.

## 1. Introduction

Magnetite (Fe_3_O_4_) is a ubiquitous iron oxide found in sediments and sedimentary rocks. It is a major carrier of sediment and rock magnetization used for numerous applications in Earth sciences, including paleomagnetic, paleoclimate, paleoenvironmental, paleogeographic, and paleontological reconstructions (e.g., Egli et al., 2013; Evans & Heller, 2003; Tauxe et al., 2010). In addition to lithogenic magnetite-bearing minerals (e.g., Chang et al., 2016), sediments contain secondary magnetite crystals formed in situ through diagenetic processes (e.g., Franke et al., 2007; Oldfield, 2007). Secondary magnetite is also produced by bacteria participating in the sedimentary iron cycle (e.g., Fortin & Langley, 2005; Lovely et al., 1987; Miot et al., 2009).

Magnetotactic bacteria (MTB) are the only known microorganisms that produce intracellular nanoparticles of magnetite in organelles called magnetosomes under a genetically controlled pathway (Uebe & Schueler, 2016). They represent some of the most ancient biomineralizing organisms, with a proposed origin of ~3 Ga (Lin et al., 2017). They are markers of oxic-anoxic transition zones in aquatic and sedimentary environments (Flies et al., 2005; Simmons et al., 2004), making them useful paleoenvironmental indicators (Chang et al., 2018; Hesse, 1994; Roberts et al., 2011; Usui et al., 2017; Wagner et al., 2021). Magnetosomes are assembled in chains, and provide the bacteria with a permanent magnetic dipole for navigation purposes (McCausland & Komeili, 2020). When MTB cells die, their magnetite chains can be trapped into sediments and subsequently fossilized (Kopp & Kirschvink, 2008; Petersen et al., 1986). MTB fossils (hereafter referred to as magnetofossils) can be preserved over geological times and contribute to sedimentary paleomagnetic and environmental records (Larrasoaña et al., 2014; Ouyang et al., 2014; Roberts et al., 2012). Their unambiguous identification and discrimination from abiotic magnetite would thus provide strong constraints on the evolution of life, biomineralization processes, and environmental conditions over geological times.

The assemblage of single domain (SD) magnetite crystals in linear chains combines two robust magnetofossil identification criteria: size and arrangement (Kopp & Kirschvink, 2008), which are key parameters granting a unique magnetic signature among all sources of natural magnetic minerals. Other types of secondary magnetite particles tend to have wide size distributions exceeding the SD stability range (e.g., Faivre et al., 2004; Gibbs-Eggar et al., 1999; Liu et al., 2005). Most importantly, the special conditions required to form isolated, non-branched strings of abiogenic SD particles (e.g., Ruder et al., 2012) are unlikely to occur frequently in natural sediments. Because the magnetic extraction procedure required to prepare concentrated samples for statistically significant TEM observations destroys the native arrangement of SD magnetite crystals in the sediment matrix, the in-situ identification of chain structures is only possible through indirect methods based on magnetic measurements of the bulk sediment. Unfortunately, magnetic methods are not always unique (e.g., Roberts et al., 2019). An additional complication arises from the fact that the lack of chain structures (e.g., Weiss et al., 2004) does not necessarily exclude a biological origin of SD magnetite particles, since forms of chain collapse that erase the original magnetic fingerprints might occur during early diagenesis.

**Figure 1.**
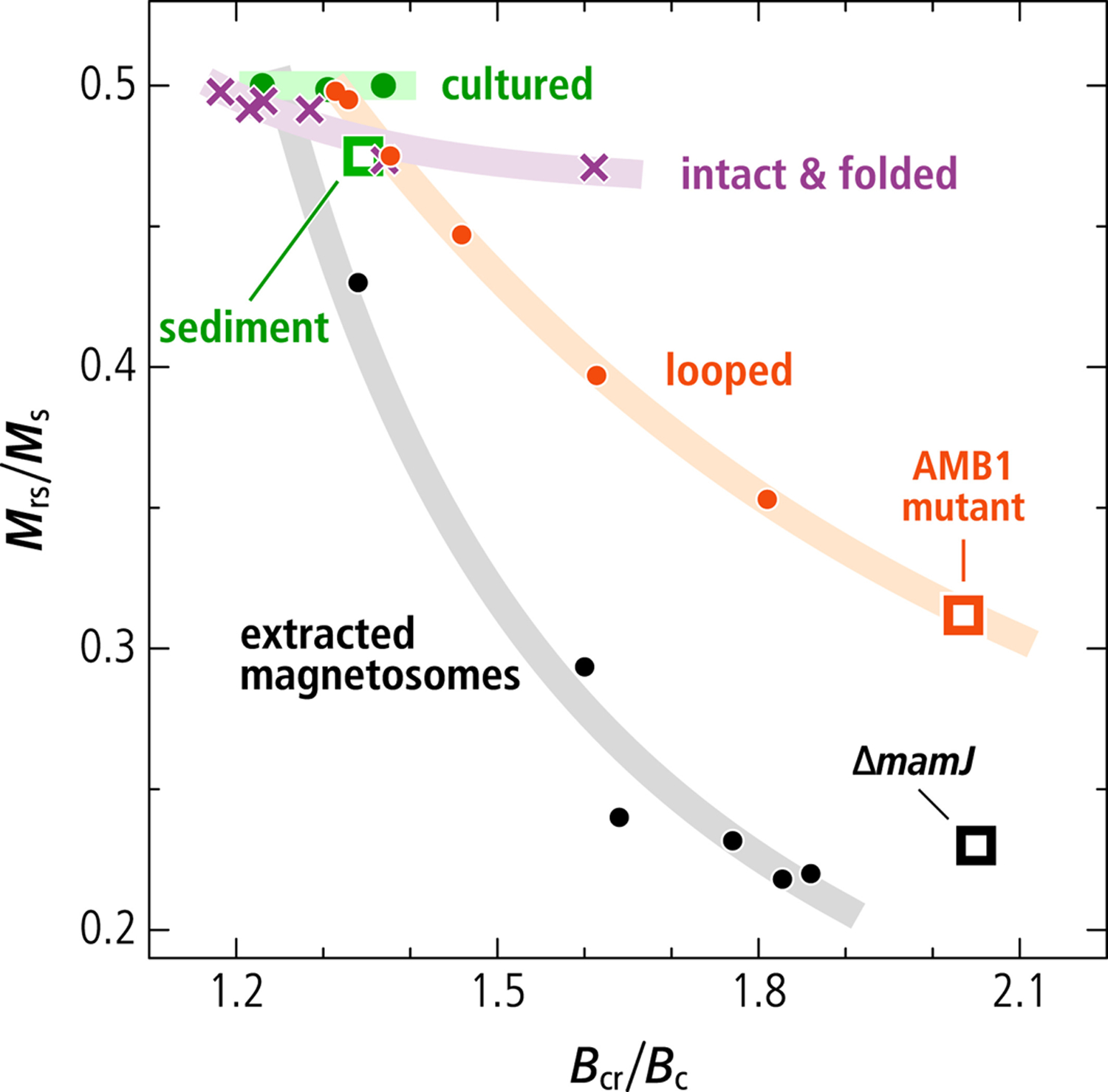
Day diagram (Dunlop, 2002) showing the hysteresis properties *M*_rs_/*M*_s_ and *B*_cr_/*B*_c_ (symbols) of the following relevant SD particle structures: magnetosome chains in intact cultured cells (“cultured,” from Li et al., 2012; Moskowitz et al., 1993; Pan et al., 2005); extractable SD particles in a magnetofossil-rich sediment (“sediment,” from Ludwig et al., 2013); the AMB-1 mutant producing looped magnetosome arrangements (“AMB1 mutant,” this work); a Δ*mamJ* mutant producing small magnetosome clusters (“Δ*mamJ*,” from Katzmann et al., 2013); magnetosomes extracted from cultured MTB (“extracted magnetosomes,” Li et al., 2012); micromagnetically simulated chains with increasing degree of axis randomization (“looped,” from Chang et al., 2019); micromagnetic simulations of intact and fold-collapsed chains (“intact & folded,” this work). Departures from *M*_rs_/*M*_s_ =0.5 for ideal, non-interacting, uniaxial SD particles follow trends (thick lines, as guide for the eye) with increasing slopes, from intact and fold-collapsed configurations with strong uniaxial anisotropy to complete randomization (extracted magnetosomes), through the intermediate trend of looped and randomized chains.

First-order reversal curve (FORC) diagrams (Egli, 2021; Pike et al., 1999; Roberts et al., 2000), which rely on the measurement of partial hysteresis curves, can be used to discriminate isolated SD magnetite particles and magnetosome chains from other structures, such as SD particle clusters and larger lithogenic (titano-)magnetite crystals containing magnetic vortices (VO) or multiple magnetic domains (MDs) (Lascu et al., 2018; Roberts et al., 2017), through a sharp signature called the central ridge (Egli et al., 2010; Heslop, Roberts, & Chang, 2014; Roberts et al., 2012). FORC diagrams of magnetofossil components isolated from sediments with principal component analysis (PCA) (Lascu et al., 2015) and through selective chemical leaching (Ludwig et al., 2013) contain signatures of VO or strongly interacting SD particle clusters with a large vertical dispersion around the central ridge, which contribute to 35%–65% of the total FORC magnetization (Table 1). FORC diagrams with these features can be explained by mixtures of (a) well-dispersed uniaxial, non-interacting SD particles, or linear chains of such particles, contributing to the central ridge (collectively referred to as UNISD in the following), and (b) non-SD or clustered SD particles contributing to the remaining parts of the FORC distribution. The associated magnetic hysteresis parameters *M*_rs_/*M*_s_ and *B*_cr_/*B*_c_ (Dunlop, 2002) are close to the theoretical limit for non-interacting SD particles and intact magnetosome chains (Table 1). In particular, hysteresis squareness (the ratio *M*_rs_/*M*_s_ of remanent and saturation magnetizations) values ≳0.44 are unrealistically large for samples containing non-SD or strongly interacting SD particles associable with 35%–65% non-central ridge FORC contributions (Ludwig et al., 2013; Figure 1).

The UNISD signature of magnetofossil-rich sediments is usually characterized by two narrow coercivity distributions (called biogenic soft, BS, and biogenic hard, BH), which are particularly evident in SD-selective magnetic responses such as the central ridge in FORC diagrams and the AF demagnetization of an anhysteretic remanent magnetization (ARM) (Egli, 2004a; Egli et al., 2010; Heslop, Roberts, & Chang, 2014). The peculiar narrowness of these coercivity distributions is caused by the relatively constant uniaxial magnetic anisotropy of the chain structure, which, together with the shape anisotropy of equidimensional (BS) and elongated (BH) magnetosomes, yields median coercivities of ~30–50 and ~65–80 mT, respectively, for these two components.

The mixed FORC properties of magnetofossil endmembers have been explained by postulating a form of chain collapse where the linear magnetosome arrangement becomes increasingly randomized after MTB death, leading to the formation of fractal-like magnetosome clusters (Chang et al., 2019; Harrison & Lascu, 2014). Isolated magnetosome chains fold and collapse if bent beyond their elastic limit, due to the magnetostatic attraction between the opposed magnetic poles at their extremities (Kiani et al., 2015; Shcherbakov et al., 1997). Bending forces might be produced by mechanical interactions with sediment particles during mixing in the bioturbated layer, and during compaction. Chains might also be stabilized by electrostatic adhesion onto larger sediment particles, such as clay platelets (Galindo-Gonzalez et al., 2009). The lack of direct in situ observations of magnetofossil structures, the mixed character of the magnetic signature of magnetofossil-rich sediments, and the sometimes-poor definition of characteristic coercivity components, leave margins for contrasting interpretations of some geological materials. Most notable examples include the controversial biogenic origin of magnetite nanoparticles in the Martian meteorite ALH84001 (Golden et al., 2004; Thomas-Keprta et al., 2000; Weiss et al., 2004), and the origin of the sudden rise in SD magnetite concentration during the Paleocene-Eocene thermal maximum (Kent et al., 2003; Kopp et al., 2007; Wagner et al., 2021; H. Wang et al., 2013).

Extracted magnetosomes obtained from MTB cultures after cell lysis tend to form large clumps with an excessively low hysteresis squareness (Kobayashi et al., 2006; Li et al., 2012) (Table 1, Figure 1). This also holds for the hysteresis squareness of smaller magnetosome clusters produced by MTB whose genes responsible for chain assembly have been deleted, namely the Δ*mamJ* MSR-1 strain in Katzmann et al. (2013) (see sections below), which might serve as an analog for the full collapse of isolated chains. Finally, the random-walk-based magnetosome assemblage algorithm of Harrison and Lascu (2014) produces a range of magnetic properties (Chang et al., 2019) comprised between the endmembers corresponding to linear chains and fractal-like particle clusters, respectively, depending on the assumed degree of randomness (Figure 1). In these simulations, FORC diagrams containing non-UNISD contributions comparable with those of magnetofossil endmembers are associated with hysteresis squareness values of ~0.4 or lower. Pedogenic magnetite, the only well-characterized natural example of non-biogenic secondary magnetite particles (Dearing et al., 2001), also features mixed UNISD and non-UNISD FORC properties (Table 1); however, in this case, the hysteresis squareness of ~0.2 is compatible with the FORC signature. Thus, current interpretations of magnetofossil signatures appear to miss structures possessing the correct combination of UNISD and non-UNISD magnetic properties.

**Figure 2.**
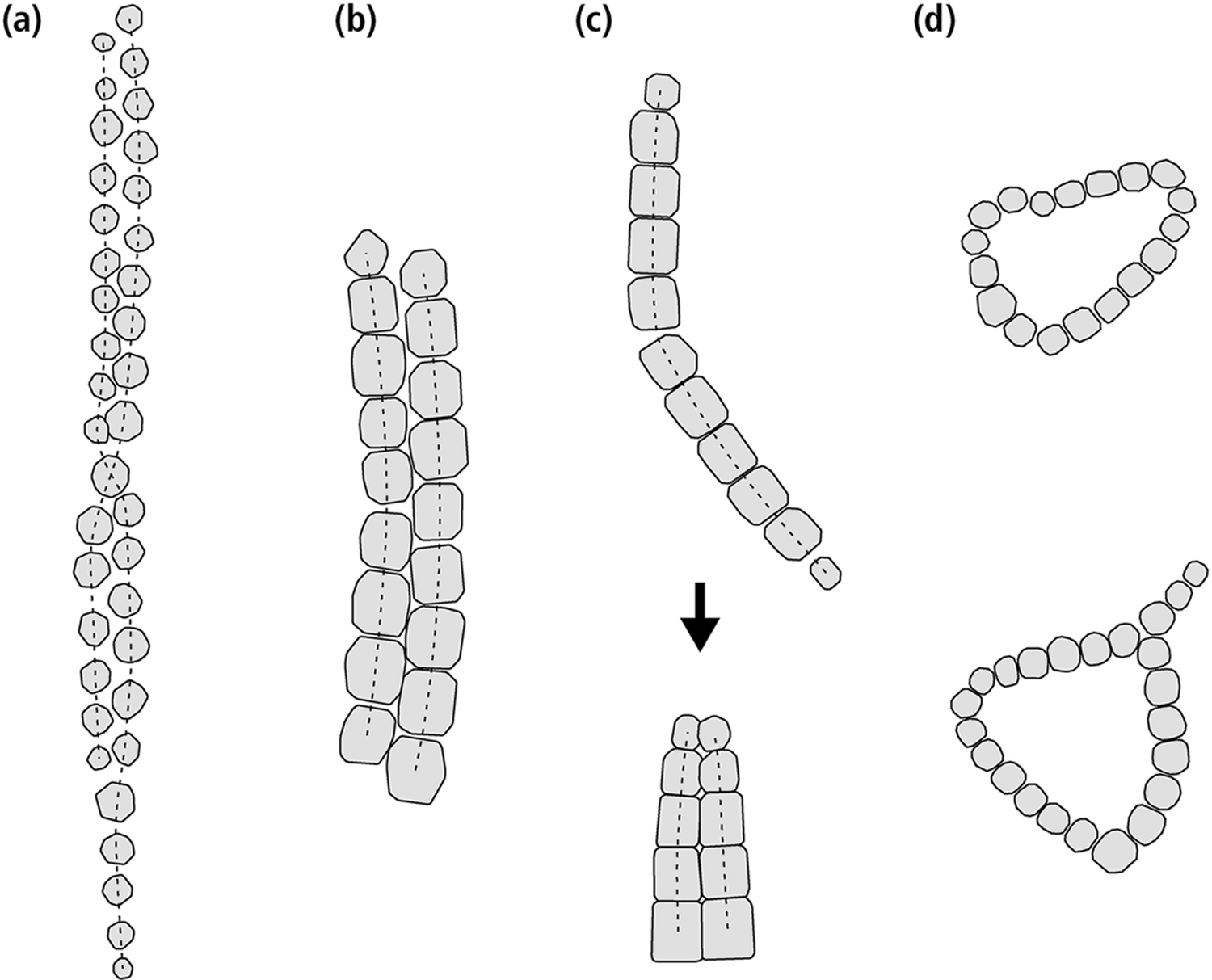
Representative examples of native and collapsed double-stranded magnetosome chains, as well as looped chains, obtained from TEM micrographs. (a) Native, twisted double-stranded chain of equant magnetosomes produced by *Magnetospirillum gryphiswaldense*. Notice the magnetosome size tapering at both ends. (b) Native double-stranded chain of prismatic magnetosomes (Lefèvre et al., 2011). Notice the staggered magnetosome arrangement. (c) Top: kinked single-stranded chain of prismatic magnetosomes (Shcherbakov et al., 1997). Bottom: complete folding of a single-stranded chain around a kink point. Notice the side-by-side arrangement of magnetosomes, and one-sided tapering of magnetosome size. (d) Two examples of rings obtained from magnetophoresis of magnetosome chains extracted from cultures of *M. magnetotacticum* (Philipse & Maas, 2002).

Excluding disordered clusters, which do not possess the correct magnetic signature, and the already investigated chain axis randomization, possible candidates for missing magnetofossil morphologies include native multistranded chains of magnetosomes produced by some MTB (Amor et al., 2020) and mechanically meaningful minimum-energy structures obtained from the collapse of isolated chains, such as rings and double strands resulting from the fold-collapse of single-stranded chains that have been bent beyond the elastic limit (Kiani et al., 2018; Philipse & Maas, 2002; von Dobeneck et al., 1987). Single-stranded and multi-stranded chains in MTB and in the derived intact magnetofossils (Figures 2a and 2b) are in a native SD state with maximum total magnetic moment, with the magnetization of all magnetosomes being parallel to the chain axis (Hanzlik et al., 2002). This is the only possible zero-field magnetic configuration for single-stranded chains, but not for multiple strands, in which intermediate states with lower magnetic moments can be nucleated with the application of strong external fields (Hanzlik et al., 2002). Intermediate remanent states are formed when the magnetic moment of one strand is reversed with respect to the others, or when the magnetic moments of individual crystals form complex patterns (Varón et al., 2013). On the other hand, structures arising from chain collapse (Figures 2c and 2d) are expected to possess native flux-closure (FC) configurations characterized by a small net magnetic moment, as observed for instance in nanoparticle rings (Dunin-Borkowski et al., 2004; Takeno et al., 2014). These configurations minimize the magnetostatic energy by suppressing the external stray field of SD-like magnetic states. The small magnetic moment of collapsed structures makes them a poor sedimentary recorder of the Earth magnetic field, contrary to intact magnetosome chains, so that the fate of fossil chains during diagenesis is expected to have important paleomagnetic implications in the case of magnetofossil-rich sediments.

FORC measurements are sensitive to transitions between high-moment (SD-like) and low-moment (FC-like) magnetic configurations and their in-field stability ranges (Egli, 2021; Nikolaisen et al., 2020). In particular, hysteresis squareness values close to 0.5, typical of randomly oriented UNISD particles, depend on the room-temperature zero-field stability of high-moment states. This stability, which enables MTB to navigate along the Earth magnetic field, has been demonstrated experimentally for native single-stranded and multi-stranded chains (Hanzlik et al., 2002; Pan et al., 2005), but no information is available for collapsed structures.

Here, we demonstrate that the unresolved magnetic signature of magnetofossils (i.e., FORC features typical of magnetostatic interactions and FC coexisting with the hysteresis squareness typical of UNISD particles) can be explained by the predominant contribution of intact or elastically deformed single- or multi-stranded magnetosome chains (non-collapsed). Contributions from fold-collapsed chains are also possible. Nucleation and annihilation of FC magnetic states explain non-UNISD signatures in FORC diagrams of magnetofossil-bearing sediments, while the strong uniaxial anisotropy of intact and fold-collapsed chains ensures that they still possess a UNISD-like hysteresis squareness of ~0.5. Our claim is supported by micromagnetic simulations of native single-stranded and double-stranded chains and fold-collapsed chains. Unfortunately, MTB producing multi-stranded chains are not available for culture in the laboratory; however, the validity of our micromagnetic calculations has been tested by comparing the magnetic signature of intact chains with some structures that may be produced by the spontaneous collapse of isolated chains. For this purpose, we produced a mutant of the magnetotactic bacterium *Magnetospirillum magneticum* strain AMB-1 synthesizing looped structures similar to the rings described by Kiani et al. (2018), and to some structures obtained after dissolution of cultured cells of the same strain (Philipse & Maas, 2002). Looped chains might form in sediment after cell disruption, but before stabilizing structures such as magnetosome membranes and the connecting cytoskeletal filament (Komeili et al., 2006) are dissolved, owing to the larger elastic response of membrane-filled gaps (Shcherbakov et al., 1997). Fold-collapsed chains, on the other hand, might form after dissolution of all biological structures, because of the much lower elastic limit associated with contacting magnetosomes (Shcherbakov et al., 1997).

From magnetic measurements and off-axis electron holography characterizations, we show that the looped chains produced by the AMB-1 mutant generate FC magnetic signatures similar to those obtained with our micromagnetic simulations of single-stranded and multi-stranded chains, while lacking the hysteresis squareness of the simulated configurations and of natural magnetofossil signatures. This demonstrates that the unique magnetic fingerprints of magnetofossils originate from strongly uniaxial magnetosome arrangements, rather than clusters, rings, and other forms of looped chains lacking a uniaxial shape. This work resolves longstanding and ongoing controversies about the interpretation of sedimentary sources of SD magnetite, establishing a theoretical and experimental framework that explains the magnetofossil signature and provides insights into the diversity of ancient MTB populations, and into mechanisms affecting the paleomagnetic recording efficiency of magnetofossil-rich sediments. A glossary of specialized terms used to describe magnetic measurements and micromagnetic modeling results is provided in the Supporting Information.

## 2. Material and Methods

### 2.1. Micromagnetic Modeling

To determine whether native multi-stranded and fold-collapsed magnetosome chains could explain the observed magnetic signatures of magnetofossil-rich sediments, we modeled their FORC properties. Micromagnetic models of high-resolution FORC measurements have been calculated for selected magnetosome morphologies and chain configurations representative of intact or elastically deformed chains, and of fold-collapsed chains. The magnetic properties of the simulated structures are determined mainly by two factors: the anisotropy of individual magnetosomes, which is controlled by their shape and crystal orientation, and in-chain magnetostatic interactions, which are controlled mainly by the chain geometry (Berndt et al., 2020). Magnetosome shapes can be classified as equant (cuboctahedral, octahedral), elongated (prismatic, elongated octahedral), and highly elongated (tooth-shaped and bullet-shaped) (Amor et al., 2020). These categories form well-defined clusters of crystal sizes and shapes within the SD stability range of magnetite (Muxworthy & Williams, 2006; Newell, 2009; Figure S1 in Supporting Information S1).

The geometry of intact single-stranded chains is controlled by the gaps between magnetosomes, the size decrease of immature crystals toward the chain extremities (tapering), and chain bending within the elastic limit. Magnetosomes in native multi-stranded chains are staggered: each magnetosome in one strand faces the gap between two magnetosomes of a nearby strand (Figures 2a and 2b). This arrangement favors the formation of strand bundles with a consistent magnetic polarity, eliminating repulsive forces (Hanzlik et al., 2002; Ruder et al., 2012). Contrastingly, magnetosomes in fold-collapsed chains are arranged side-to-side, and size tapering occurs only at one end, the other end being the kink point of the native chain (Figure 2c). The side-to-side magnetosome arrangement maximizes lateral attractive forces between strands with opposite magnetic polarities (Varón et al., 2013), as they result from fold-collapse. Fold-collapsed chains can be observed inside freeze-dried cells (e.g., Figure 2b in Lins and Farina, 2004), where collapse is likely triggered by cell shrinking.

The full variability of natural chain structures can only be partially reproduced because geometric parameters must be extrapolated from few observations. Therefore, we selected combinations of two magnetosome morphologies (cuboctahedral and prismatic), and three chain geometries (single-stranded, native double-stranded, and double-stranded from collapse by folding), as representative examples of magnetic endmembers dictated by the magnetosome aspect ratio (equant vs. elongated), and the existence of flux-closure configurations (single-stranded vs. double-stranded). We generated ~10^5^ synthetic chains with random orientations and realistic geometries for each of the six categories, obtained by combining the above-mentioned magnetosome morphologies and chain configurations (see Supporting Information S1 for details).

Micromagnetic modeling has been performed in two steps using an energy minimization method. First, randomly oriented synthetic chains have been generated for each of the six configurations. Magnetosome-specific control parameters include size, shape, and crystal axes orientation, based on realistic distributions obtained from the literature (Figure S1 in Supporting Information S1). Chain-specific control parameters include magnetosome gaps, number of crystals, size tapering, and chain bending within the elastic limit. The geometry of double-stranded chains is additionally controlled by the lag of one strand with respect to the other, and by twisting of the two strands about a common axis (Figure 2a). The distributions of these parameters (Table S1 in Supporting Information S1) have been empirically chosen to match images reported in the literature. Representative examples of synthetic double-stranded chains are shown in Figures S2–S5 in Supporting Information S1. Next, FORC measurements comprised between ±0.3 T have been simulated in 1 mT steps using a micromagnetic model of the chain magnetization. These calculations are based on the local minimization of the total energy determined by the magnetocrystalline and shape anisotropy of individual crystals, the Zeeman energy in the applied field, and the energy of magnetostatic interactions between pairs of crystals. Because FORC measurements consist of a series of field sweeps starting at positive saturation, where there is only one local energy minimum (LEM), the evolution of LEM states is uniquely determined.

Like in Harrison and Lascu (2014), we assumed that individual crystals are homogeneously magnetized, so that the magnetic state of a single-stranded or double-stranded chain containing *N* magnetosomes is entirely specified by the vectors ***θ*** = (*θ*_1_,…,*θ*_*N*_) and ***φ*** = (*φ*_1_,…*,φ*_*N*_) of polar and azimuthal angles of the magnetic moment directions **u**_*i*_ = (cos*φ*_*i*_ sin*θ*_*i*_, sin*φ*_*i*_ sin*θ*_*i*_, cos*θ*_*i*_) for *i* = 1…*N*. The total free energy of the chain is then given by

(1) $$F=\sum_{i=1}^{N}F_{i}^{\text{c}}+F_{i}^{\text{s}}+F_{i}^{\text{z}}+\sum_{i=1,j>i}^{N}F_{ij}^{\text{int}}$$

where $F_{i}^{\text{c}}$, $F_{i}^{\text{s}}$, and $F_{i}^{\text{z}}$ are function of (*θ*_*i*_*, φ*_*i*_) expressing the magnetocrystalline anisotropy, the shape anisotropy, and the Zeeman energy of the *i*th magnetosome, respectively, and $F_{ii}^{\text{int}}$ the magnetostatic interaction energy between magnetosomes *i* and *j* (see the Supporting Information for details). We assumed that one of the <111> crystallographic axes is parallel to the chain axis, up to a small random misorientation angle, since this is the typical crystal orientation observed for cuboctahedral and prismatic magnetosomes (Körnig et al., 2014; Pósfai et al., 2013; Simpson et al., 2005).

Unlike in Harrison and Lascu (2014), we calculated the shape anisotropy and the magnetostatic interaction energies for the actual shape of the modeled magnetosomes. This distinction does not matter in the case of triaxial ellipsoids used to represent small deviations of equidimensional magnetosomes from isometric shapes, since uniformly magnetized ellipsoids can be treated as point dipoles with demagnetizing tensors given by Osborn (1945). The shape of prismatic magnetosomes, on the other hand, has been represented by cylinders with a slight random ellipticity and chamfered ends that approximate faceting (e.g., Buseck et al., 2001). Chamfering changes the demagnetizing tensor by <10% for the typical geometries required to approximate prismatic magnetosomes, so that the analytical solution for cylinders (Joseph, 1966) has been extended through an empirical correction factor obtained numerically (see the Supporting Information). The magnetostatic interaction energy, on the other hand, is strongly affected by details of the crystal shape, such as the size and orientation of gap-delimiting facets. In case of triaxial shapes, the interaction energy between two uniformly magnetized particles with magnetic moments parallel to the unit vectors **u**_1_ and **u**_2_ can be expressed in tensor form as

(2) $$F_{12}^{\text{int}}\left(u_{1},u_{2}\right)=\sum_{i,j=1}^{3}F_{ij}\left(u_{1}\cdot e_{i}\right)\left(u_{2}\cdot e_{j}\right)$$

with **e**_*i*_ being the basis vectors of a common coordinate system and *F*_*ij*_ coefficients representing the cases where each magnetic moment is parallel to one of the **e**_*i*_. These coefficients need to be calculated numerically only once for each chain configuration, before the FORC simulation is started, by integrating the surface charges generated by a homogeneous magnetization (see the Supporting Information for details).

The detailed definition of magnetosome shapes and chain geometries does not produce drastic changes in the magnetic properties with respect to simpler cases, but it is a physically meaningful way to eliminate the granularity of excessive simplifications. This is particularly important for discriminating intrinsic magnetic features, such as the FORC lobes generated by transitions between multiple magnetic states, from random features. Parameter randomization occurs at three levels: first, chains with different characteristics are generated by using global parameters, such as the mean size and shape of magnetosomes (Figure S1 in Supporting Information S1), the size tapering degree toward the chain ends, the mean size of gaps, and staggering of double-stranded chains. Next, the size, shape, and arrangement of individual crystals is further randomized to simulate deviations from the ideal geometry. Finally, the chain is twisted and bended within the elastic limits. The detailed procedure is described in the Supporting Information.

The homogeneous magnetization approximation, which is needed to speed up calculations and cover the whole parameter space with a sufficiently large number of high-resolution FORC simulations, holds reasonably well in all applied fields, including those immediately preceding transitions between magnetic states, for magnetosome sizes up to ~50 nm, as seen by comparison with full micromagnetic models (Berndt et al., 2020). At larger sizes, the validity of this approximation declines progressively, as helical configurations start to nucleate inside the end magnetosomes. Nevertheless, the coercivities of remanence calculated by Berndt et al. (2020) for single-stranded chains of equidimensional and elongated magnetosomes are compatible with the coercivity distributions obtained from our simulations, so that the errors introduced by our approximation are not expected to exceed the uncertainties associated with a poor knowledge of the real distribution of model parameters. Simulated FORC measurements (Figure S6 in Supporting Information S1) have been further processed with VARIFORC (Egli, 2013), using minimal smoothing parameters (*s* = 1 over the central ridge, and *s* = 4, *λ* = 0.08 over the remaining parts of the FORC diagram) to eliminate statistical noise.

The role of chain geometry on the stabilization of high-moment states, which is essential for obtaining elevated hysteresis squareness values, has been investigated by calculating the energy barrier that must be overcome for a thermally activated transition to a different state at room temperature and in a null field. For this purpose, we considered four prototypical arrangements of identical, equidimensional magnetosomes with no intrinsic anisotropy: (a) single-stranded chains, (b) native double-stranded chains, (c) fold-collapsed chains, and (d) circular rings. Optimal transition paths between LEM states have been calculated with the nudge-elastic-band and action minimization technique of Fabian and Shcherbakov (2018), upon identifying the magnetic moments of individual magnetosomes with the magnetic moments of elemental cells used in full micromagnetic simulations (see the Supporting Information for details).

The choice of an adequate initial path is crucial for obtaining a globally optimized transition between states. Single-stranded chains of >4 crystals are known to reverse their remanent magnetization by nucleation of a reversed domain at one end (Hendriksen et al., 1994; Newell, 2009) and subsequent propagation of this domain to the other end. In this case, an adequate initial approximation of the reversing path is generated from an initial two-domain structure obtained by forcing the magnetic moment of the middle magnetosome to a direction perpendicular to the chain axis. This configuration is then relaxed while the strength of dipolar interactions in one of the two domains is slightly weakened to break the initial symmetry and favor its denucleation (Figure S7a in Supporting Information S1). The high-moment configuration of large rings consists of two domains separated by perpendicular magnetic moments pointing to the same direction (Figure S7b in Supporting Information S1). The transition to a zero-moment FC state with clockwise or counterclockwise magnetic moment arrangement is simulated in the same manner as for linear chains by denucleating one of the two domains. Finally, the transition path of double-stranded chains from the high-moment state, where the two strands are magnetized in the same direction, to the low-moment state, where the two strands have opposed magnetizations, requires the nucleation of a reverse domain at the extremity of one strand (Figure S7c in Supporting Information S1). The nucleated domain creates an energetically favored FC configuration with the other strand, so that the transition to a complete FC state is obtained by growing the reverse domain until it reaches the other chain extremity.

The algorithms for chain construction and FORC and energy barrier calculations have been implemented in Mathematica 12. Data visualization, which includes all figures in this paper, has also been performed in Mathematica 12. Scripts for the simulation of FORC diagrams and thermally activated transitions between zero-field states, as well as the Mathematica notebooks used to run the simulations are downloadable at the link provided in the availability statement.

### 2.2. Deletion Plasmid Construction and Generation of the Δ*mamJ*Δ*limJ*ΔMIS AMB-1 Mutant Strain

**Figure 3.**
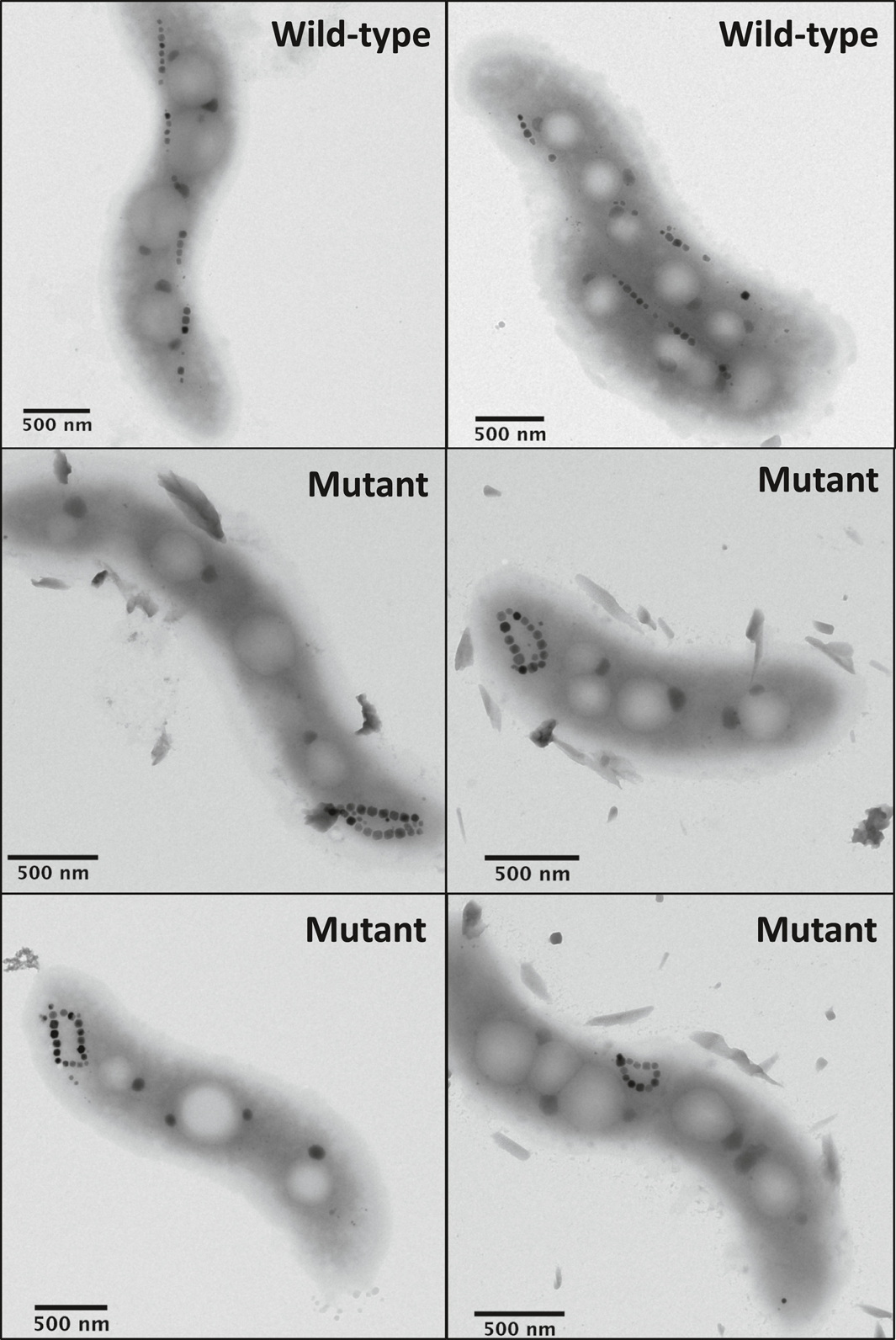
Electron microscopy images of wild-type and mutant AMB-1 cells.

To obtain a term of comparison for possible collapsed chain configurations and validation of our micromagnetic modeling, we generated a mutant AMB-1 strain with looped magnetosome chains (Figure 3). Magnetosome formation in MTB requires genes contained in a specific portion of the genome called the magnetosome island (MAI) (Murat et al., 2010). The AMB-1 genome also contains a small version of the magnetosome island called the magnetotaxis islet (MIS) (Rioux et al., 2010). The MIS contains several genes, some of which perform redundant functions with their homologs in the MAI (Abreu et al., 2014). Previous work in *Magnetospirillum gryphiswaldense* strain MSR-1, a close relative of AMB-1, showed that the loss of the MAI gene *mamJ* results in collapse and aggregation of magnetosome chains (Scheffel et al., 2006). Surprisingly, the deletion of *mamJ* and its other MAI homolog *limJ* in AMB-1 does not produce collapsed chains and instead results in minor disruptions to the continuity of the chain (Draper et al., 2011). We reasoned that redundant functions within the MIS might account for the dramatic differences between *mamJ* mutants in AMB-1 and MSR-1. Thus, a mutant strain (Δ*mamJ*Δ*limJ*ΔMIS) was produced by deleting the entire MIS and two MAI genes (*mamJ* and *limJ*) from AMB-1 genome. Detailed procedures for plasmid construction and AMB-1 transformation are provided in the Supporting Information.

### 2.3. Bacterial Cultures

*Magnetospirillum magneticum* strain AMB-1 (ATCC700264) and the mutant AMB-1 strain described in Section 2.2 were cultivated following ATCC recommendations in 500-ml screw-caped bottles until the end of the exponential phase. Bottles were filled with 300 ml of growth medium and placed in a glove box with controlled atmosphere (10% O_2_, 90% N_2_) at 30°C after inoculation (1/100). The sole iron source in bacterial growth medium was Fe(III)-citrate added at 150 μM.

### 2.4. Transmission Electron Microscopy

Wild type and mutant AMB-1 cells were deposited on copper grids coated with a Formvar and carbon films and characterized with an FEI Tecnai 12 transmission electron microscope operating at 120 kV.

### 2.5. Native Magnetic Moment Measurements

Magnetic characterizations of the mutant AMB-1 strain suggest that its looped magnetosome chain configurations possess a low net magnetic moment (see Section 3.3). We thus investigated the native magnetic state of wild-type and mutant AMB-1 strains at the population level by comparing the magnetization of two different preparations of aqueous cell suspensions in a maximum external field of 2 mT, using a vibrating-sample magnetometer (VSM). The external field is sufficiently large to partially align the cells, but not large enough to alter their native magnetic configuration. In this case, the magnetization of the suspension is expected to be proportional to the mean strength of the magnetic moments of individual cells. The first preparation contains cells directly taken from the culture and is used to assess the native magnetic moments. In the second preparation, the suspension is previously exposed to a 200-mT field. This field is strong enough to reset the native magnetic states, replacing them with a saturation remanent state that corresponds to the maximum magnetic moment that can be maintained in a null field. Cell suspensions for these experiments were prepared by centrifugation of AMB-1 cultures (8,000 rpm, 10 min), and subsequent suspension in 10 ml of phosphate buffer (PBS). 100 μl of cell suspension were transferred in plastic vials (diameter of 4 mm) and placed in a Lakeshore Micro-Mag 3900 VSM for acquisition of remanent magnetizations at room temperature.

### 2.6. Off-Axis Electron Holography

VSM characterizations of mutant AMB-1 demonstrated a vanishingly small native magnetic moment of the bacteria, which can be explained by the looped chain structures being in an FC state. To further confirm this hypothesis, we mapped the magnetic flux in the looped chains at the single-crystal level with off-axis electron holography at the Centre d’Elaboration de Matériaux et d’Etudes Structurales (CEMES) in Toulouse, France. Electron holography is an interferometric method that correlates morphological and local magnetic characterizations of magnetic materials. It allows quantitative mapping of the in-plane flux inside the magnetite chains produced by AMB-1 at the nanometer scale. Both wild-type and mutant AMB-1 strains were cultivated in 10-mL glass tubes following the protocol described above. Cells were centrifuged (8,000 rpm, 10 min) and suspended in 100 μl of phosphate-buffered saline (PBS). They were then deposited on copper grids coated with an ultra-thin carbon membrane. Off-axis electron holography was carried out using a Hitachi HF3300 C microscope operated at 300 kV and equipped with a cold field emission gun and a spherical aberration corrector (CEOS B-Corr). Electron holography experiments were performed in a specific corrected Lorentz mode, allowing a spatial resolution down to 0.5 nm in a magnetic field-free sample environment (Snoeck et al., 2014). All holograms were recorded in a two-biprism configuration to avoid artifacts linked to Fresnel fringes and to set separately the interference area size and the fringe spacing (Harada et al., 2004). The fringe spacing is equal to 1 nm (7 pixels) allowing for a spatial resolution of 1.5 nm for the treated magnetic phase images. The exposure time was set to 1 nm using dynamic automation acquisition for removing instabilities and applying the fringe π-shift method (Gatel et al., 2018; Volkov et al., 2013). Phase and amplitude images were extracted from the holograms by using homemade software based on fast-Fourier transform approach. The magnetic and electrostatic contributions have been separated by acquiring two holograms for which the sample has been switched upside down (flipped 180°): the magnetic contribution was obtained by evaluating the difference of the phase images from the two holograms divided by two.

### 2.7. FORC Measurements

The magnetic properties of the wild-type and mutant AMB-1 strains were characterized with high-resolution FORC analyses using a VSM. AMB-1 cultures were recovered by centrifugation (8,000 rpm, 10 min). Bacterial pellets were transferred in a 1.5-ml Eppendorf tube and dried at room temperature under anoxic conditions in a glove box ([O_2_] < 1 ppm) to prevent magnetite oxidation. Samples were stored under anoxic conditions until being measured with a Lakeshore 8600 VSM. High-resolution FORC measurements were performed in steps of 0.3 mT with a stepwise approach to the reversal field to avoid overshooting artifacts (Wagner et al., 2021), and a pause of 1 s at reversal. Measurements have been processed with VARIFORC (Egli, 2013) using a minimum smoothing factor *s* = 2 across the central ridge, and *s* = 4, *λ* = 0.14 over the remaining parts of the FORC diagram.

## 3. Results

### 3.1. Chain Geometry and Magnetic States

**Figure 4.**
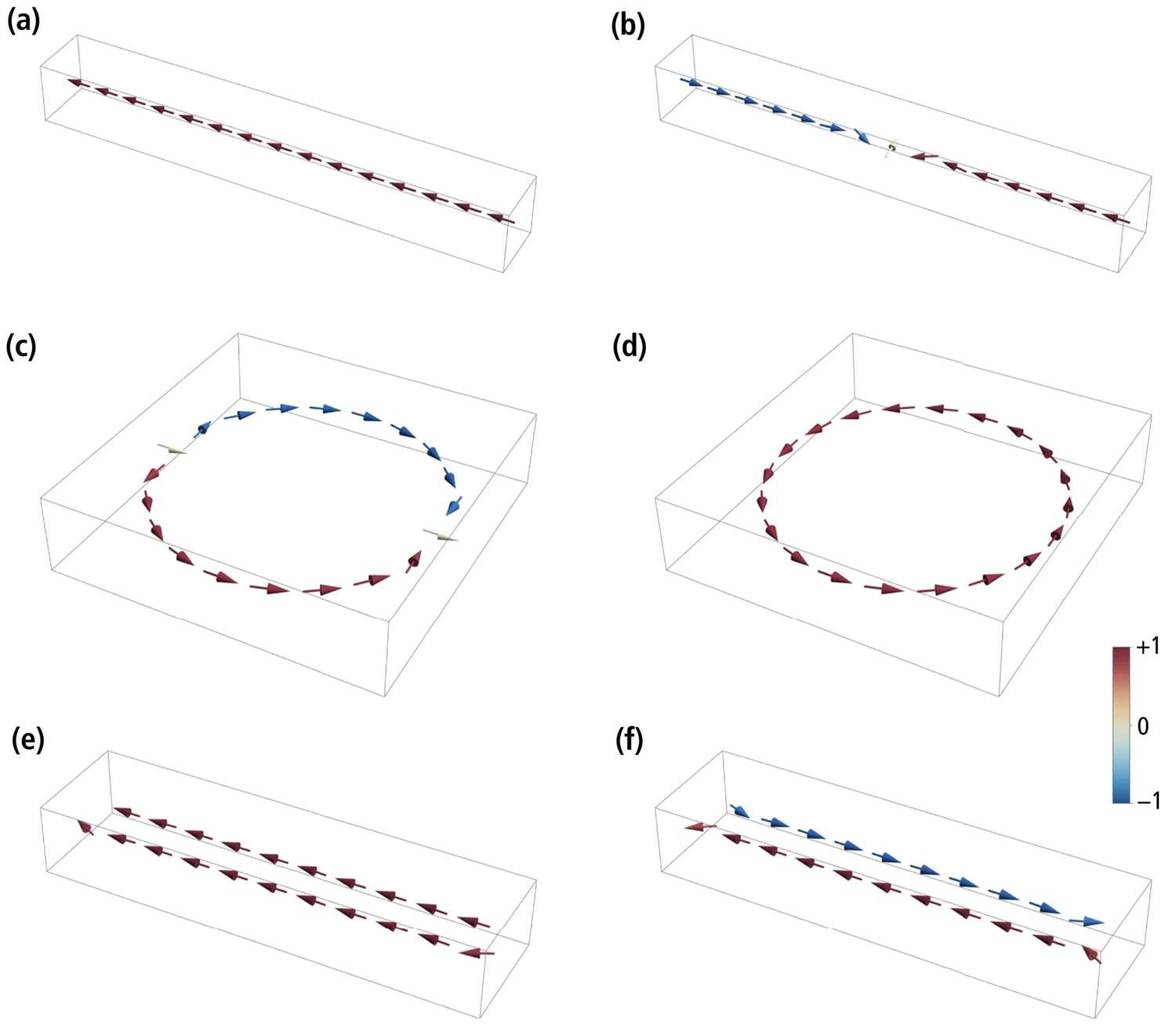
Numerically simulated zero-field magnetic states in idealized chains of identical magnetosomes with dipolar interactions but no magnetic anisotropy. The magnetic moments of individual crystals are indicated by arrows with the color coding corresponding to the axial component. (a) High-moment (SD-like) state of a single-stranded chain with 15 magnetosomes. (b) Two-domain state of the same chain as in (a), during a thermally activated magnetization reversal. (c) High-moment state of a ring with 20 magnetosomes, consisting of two symmetric domains with opposed vorticity. (d) Low-moment, flux-closure (FC) state of the same ring as in (c). (e) High-moment (SD-like) state of a double-stranded chain with 11 + 10 staggered magnetosomes. (f) Low-moment (FC) state of the same double-stranded chain as in (e). See Movies S1–S4 for the corresponding transitions between states.

The existence of one or more than one pair of magnetic states, and the stability of these states against thermal relaxation and applied fields, depends on the interplay between the magnetic anisotropy of individual crystals and the magnetostatic interactions. The stabilizing or destabilizing effect of magnetostatic interactions depends in turn on the chain geometry. Because magnetosome elongation is the main factor controlling the magnetic anisotropy of individual crystals, and because elongation, if present, is parallel to the chain axis and has a stabilizing effect, the minimum stability granted by the chain geometry is best investigated for the case of equidimensional magnetosomes with no magnetocrystalline anisotropy and purely dipolar interactions, in a null field, as in Hendriksen et al. (1994). In this case, single-stranded chains possess only one pair of remanent states with all magnetic moments parallel or antiparallel to the chain axis (Figure 4a). Newell (2009) showed analytically that, in a null field, the magnetic moment of chains with up to four crystals is reversed by symmetric fanning, while the reversal mode of longer chains is domain nucleation. Fanning among the end crystals also precedes field-induced chain moment reversals at small angles between chain axis and field direction, as shown in Berndt et al. (2020) and in our FORC simulations.

The energy barrier of reversal modes defined by local symmetries, such as fanning, is proportional to the number of crystals, so that, for longer chains, these modes are always replaced by a nucleation mode that involves only few crystals at a time. Because end magnetosomes are less strongly coupled to the rest of the chain, thermally activated transitions occur by nucleating a domain with reversed magnetization at one end of the chain. The two domains with opposed axial moments are separated by a transition region of ~3 crystals whose magnetic moments deviate strongly from the chain axis. Once the two domains are formed, the moment reversal is completed by moving the transition region toward the other end of the chain (Figure 4b), until the original domain is denucleated (Movie S1). The energy barrier that needs to be overcome for this transition is Δ*E* = *β*_0_*k*_*B*_*T*, where *k*_*B*_ is the Boltzmann constant, *T* the absolute temperature, and *β*_0_ the so-called Boltzmann factor. At room temperature (*T* = 293K), *β*_0_ ≈ 836 for *N* = 15 magnetite magnetosomes with a diameter of 50 nm separated by 5 nm gaps. The energy barrier depends only weakly on the number of magnetosomes if *N* > 6 (Table 2). For comparison, the stability threshold predicted by the Néel-Arrhenius law *τ* = *τ*_0_exp(*β*_0_), where *τ* is the time constant of the spontaneous magnetization decay and *τ*_0_ ≈ 0.1–1 ns (Moskowitz et al., 1997), is *β*_0_ ≈ 59 for geologic time scales (*τ* = 1 Ga), ~32 for the typical lifespan of a cell (*τ* ≈ 1 day), and ~25 for the typical time required to measure a single FORC (*τ* ≈ 1 min). This yields a lower crystal size limit of ~17 nm for *τ* ≈ 1 min and magnetosome gaps of 0.1 particle diameters. For comparison, Newell (2009) obtained a limit of ~15 nm for the same chain geometry and *N* = 6, using a purely analytical approach and upon including the magnetocrystalline anisotropy of magnetosomes with <111> axes parallel to the chain axis. The latter adds stability to the magnetic moments, explaining the slightly smaller size limit.

The large energy barrier generated by axial magnetostatic interactions ensures that field-induced transitions between the two stable states of single-stranded chains occur in proximity of the theoretical switching fields predicted by micromagnetic models that neglect thermal activations, such as the one used here and those of Harrison and Lascu (2014) and Berndt et al. (2020). Nevertheless, thermal activations are still sufficiently large to produce a measurable vertical offset of the central ridge (Egli, 2013, 2021), due to the intrinsic time asymmetry of the FORC measurement protocol (Berndt et al., 2018). The central ridge offset of high-resolution FORC measurements of magnetofossil-rich sediments, which is of the order of 0.3–0.5 mT (Egli et al., 2010; Ludwig et al., 2013; Wagner et al., 2021), can be explained by a Stoner-Wohlfarth model of thermally activated UNISD particles (Berndt et al., 2018; Lanci & Kent, 2018) with *β*_0_ ≈ 400–600.

The high-moment remanent state of magnetosome rings, obtained by decreasing the applied field from saturation to zero, recalls the “onion state” of toroidal magnetite nanoparticles described by Lewis et al. (2020), in which the magnetic flux enters the ring from one side, flows past the central hole, and exits on the other side. In the limit case of large rings, the “onion state” is equivalent to two magnetic domains with clockwise and counterclockwise fluxes tangential to the circumference, separated by two transition zones similar to that of a single-stranded chain with two domains (Figure 4c). Deviations from the tangential magnetization in the transition zones point to the in-plane component of the field used to impart a saturation remanence. Unlike the case of single-stranded chains, where most of the energy barrier is associated with the nucleation of a new domain, transitions to a zero-moment FC state (Figure 4d) occur with little additional energy by moving one of the two domain boundaries until it merges with the other boundary, leaving a single domain with circular magnetization (Movie S2). The energy barrier of this transition is *β*_0_ ≈ 27 for a ring made of 20 magnetosomes with 5 nm gaps. The FC state is much more stable, as seen from the energy required for the opposed transition (*β*_0_ ≈ 1,630). In practice, the saturation remanence of *N* = 20 rings will decay exponentially to zero (which is the net magnetization of the FC state) in ~9 min. The saturation remanence of smaller rings is better stabilized (e.g., *β*_0_ ≈ 55 for *N* = 16), but still much less than single-stranded chains with the same number of magnetosomes.

The high-moment remanent state of double-stranded chains is similar to that of single-stranded chains, with the two strands possessing nearly axial moments with same polarity (Thomas et al., 2008; Figure 4e). The magnetic moments of the end magnetosomes are not completely aligned with the chain axis, due to the reduced axial magnetostatic coupling and the stray field concentration at the chain extremities. Transitions to the low-moment remanent state, where the two strands with opposed polarities form a closed flux loop (Figure 4f), occur by nucleating a reverse domain in one of the two strands. An example can be seen in Figure 10 of Simpson et al. (2005), where two antiparallel domains in one strand are stabilized by a kink. The transition is accomplished by extending the reverse domain until the original domain is fully denucleated (Movies S3 and S4). Native double-stranded chains with staggered magnetosomes (Figures 2a and 2b), and double-stranded chains with facing magnetosomes resulting from fold-collapse (Figure 2c), undergo the same type of transitions between their high-moment and low-moment states; however, the high-moment state of native double-stranded chains is more stable than that of fold-collapsed chains (e.g., *β*_0_ ≈ 751 vs. 242 for *N* = 11 + 11, Table 2), due to the staggered magnetosome arrangement (Hanzlik et al., 2002; Ruder et al., 2012). In both cases, high-moment states are stable over geologic times. The energy barriers that need to be overcome for the reverse transitions, from low-moment to high-moment states, are slightly larger than those of single-stranded chains.

### 3.2. Micromagnetic FORC Models

**Figure 5.**
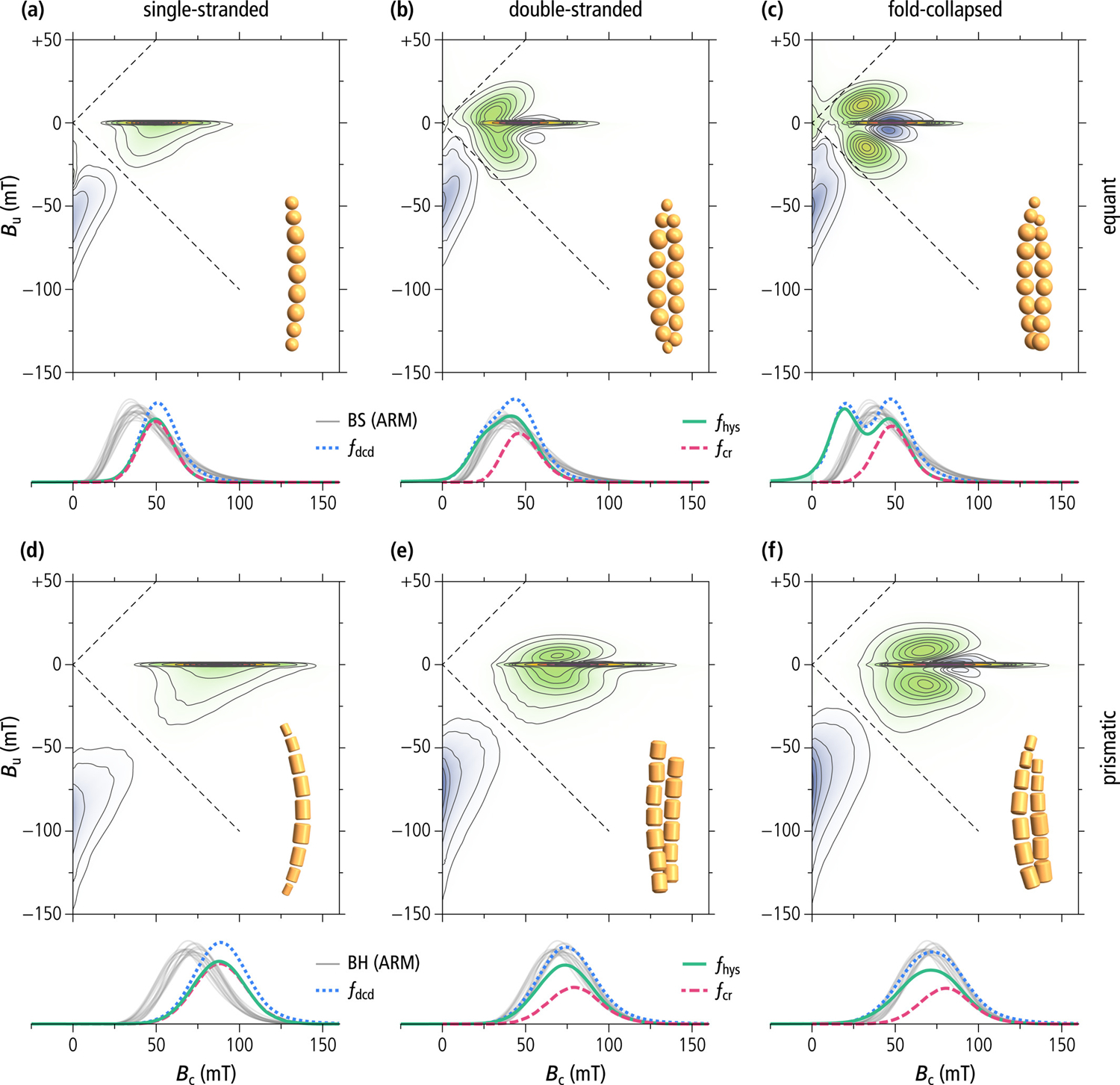
Micromagnetically simulated FORC diagrams of single-stranded chains (left), native double-stranded chains (middle) and fold-collapsed chains (right) of equidimensional (top) and prismatic (bottom) magnetite magnetosomes. Each FORC diagram corresponds to ~10^5^ randomly oriented chains with a realistic distribution of geometric parameters as explained in the text. Coercivity distributions obtained from the simulated FORC data (*f*_dcd_—DC demagnetization of saturation remanence, *f*_hys_—irreversible component of the ascending hysteresis branch, *f*_cr_—central ridge) are shown below, together with coercivity distributions of the biogenic component BS (gray lines in (a–c)) and BH (gray lines in (d–f)), obtained from AF demagnetization curves of ARM (from Egli, 2004a). All FORC diagrams share the same color scale (blue—negative values, green to violet—positive values) with *q* = 5%, 10%, 20%, 30%, 40%, 50%, 60%, 70%, 80%, and 90% quantile contours. The FORC region enclosed by the *q*-quantile contour contributes to a fraction 1 – *q* of the total FORC integral (Egli, 2021). Dashed lines indicate the left boundary of the so-called memory region of the FORC space.

Simulated FORC diagrams of single-stranded chains (Figures 5a and 5d) possess all the expected characteristics of UNISD particles (Newell, 2005), as also seen in cultured MTB (Jovane et al., 2012; Li et al., 2012; H. Wang et al., 2013; Y. Wang et al., 2015). These characteristics include (a) a sharp central ridge along *B*_u_ ≈ 0, which is produced by the collective switching of all magnetic moments within individual chains, (b) lack of contributions in the upper quadrant, and (c) a distribution of positive and negative amplitudes in the lower quadrant, nearly antisymmetric with respect to the *B*_c_ = −*B*_c_ diagonal, produced by the reversible rotation of the magnetic moments in the applied field (Newell, 2005; Egli, 2021). FORC measurements define three coercivity distributions (Egli, 2021) associated with (a) irreversible magnetization changes along the lower branch of the hysteresis loop (*f*_hys_), (b) the remanent magnetization of a so-called DC or backfield demagnetization curve (*f*_dcd_), and (c) the central ridge (*f*_cr_). In our simulations of single-stranded chains, all three distributions have identical, Gaussian-like shapes dictated by the in-field (*f*_irr_, *f*_cr_) and zero-field (*f*_dcd_) magnetic moment changes associated with transitions from negative to positive high-moment states. The coercivity distributions of simulated single-stranded chains of equidimensional and prismatic magnetosomes are slightly narrower and biased toward ~30% higher fields with respect to the biogenic components BS and BH commonly found in magnetofossil-rich sediments (A. P. Chen et al., 2014; Egli, 2004a; Heslop, Roberts, & Chang, 2014). This difference can be explained in part by the fact that micromagnetic calculations were based on stoichiometric magnetite, while real magnetofossils are often partially or completely maghemitized (Housen & Moskowitz, 2006; Vali et al., 1987). The maghemite endmember (γ-Fe_2_O_3_) has a ~20% lower spontaneous magnetization, and a similar reduction in shape anisotropy is therefore expected. Furthermore, the magnetosome gap in real magnetofossils might be larger than assumed in our calculations, producing a further reduction of the switching field (Berndt et al., 2020).

Simulated FORC diagrams of double-stranded chain configurations feature typical flux-closure signatures (Figure 5b, 5c, 5e, and 5f) as described in Egli (2021). These features are fully developed only in fold-collapsed chains of equidimensional magnetosomes, resulting in a slightly constricted hysteresis loop (Figure S6e in Supporting Information S1) and clearly identifiable pairs of positive and negative lobes surrounding the central ridge (Figure 5c). These lobes are generated by transitions between high- and low-moment states with different in-field stability ranges (see Egli, 2021, and Figure 57 therein for a detailed explanation). The central ridge, on the other hand, is associated exclusively with the denucleation of FC configurations. Magnetosome elongation and staggering in native double-stranded chains add stability to the high-moment state, as seen by the partial suppression of the negative lobes around the central ridge (Figures 5b, 5e and 5f). The only natural example of FORC signature produced by multi-stranded chains is that of a concentrate of wild-type *M. bavaricum* cells extracted from a pond sediment (Roberts et al., 2012). This sample features positive contributions in the upper quadrant of the FORC diagram which might be attributed to the denucleation of FC states.

Contrary to the AMB-1 mutant (see below) and other examples of magnetic systems featuring this type of FORC signature, such as single-vortex (SV) particles (Dumas et al., 2007; Katzmann et al., 2013; Pike & Fernandez, 1999; Roberts et al., 2017), the hysteresis squareness of our modeled double-stranded chains is only slightly lowered with respect to the uniaxial SD case (*M*_rs_/*M*_s_ = 0.471, Table 1, Figure 1), and fully compatible with magnetofossil signatures (Ludwig et al., 2013). Squareness values close to 0.5 for all simulated double-stranded and fold-collapsed chains imply that they possess stable SD states in zero field, as observed on native chains (Li et al., 2015), and unlike the looped chains of the AMB-1 mutant. Stable high-moment states in zero field require the hysteresis loop to be fully reversible until the applied field reverses sign, which means that the FORC function is fully comprised within the so-called memory region (dashed lines in Figure 5).

The central ridge coercivity distributions of double-stranded chains are almost identical to those of single-stranded chains made of the same type of magnetosomes. This is expected from the similar stability of high-moment states in single-stranded chains and of FC states in double-stranded chains (Table 2). Non-central ridge coercivity distributions, on the other hand, contain additional low-field contributions related to the nucleation of FC states. In the case of equant magnetosomes, these contributions produce a second peak (fold-collapsed chains) or a shoulder (native chains) at *B*_c_ ≈ 15 mT. In the case of prismatic magnetosomes, coercivity distributions remain unimodal but become wider, and the peak position is lowered by ~10 mT with respect to the central ridge. Non-central ridge coercivity distributions of native double-stranded chains are remarkably similar to the biogenic coercivity components BS and BH (Figures 5b and 5e).

### 3.3. Mutant AMB-1 Strain With Looped Chains

Unlike the Δ*mamJ* MSR-1 mutant, which produces agglomerated clusters of magnetosomes, the phenotype of our AMB-1 mutant contains looped magnetosome chains with necklace structures located either at one of the poles or at the center of the cell (Figure 3). Looped chains in the AMB-1 mutant strain show different degrees of opening, as expected from the 2D projection of circular structures with different plane orientations. Among the 109 mutant cells imaged with TEM, only 7 showed loops sufficiently closed to be confused with double-stranded chains. Magnetosomes in the mutant strain fall mostly within the stable SD size range of single-stranded chains (Muxworthy & Williams, 2006; Newell, 2009). The magnetic behavior of bacteria was assessed using the magnetic coefficient *C*_mag_, which relies on the differential measurement of a culture’s optical density when a magnet is oriented either vertically or horizontally close to the cell suspension. *C*_mag_ is defined as the ratio of the maximum and minimum optical density and quantifies the capacity of bacteria to orientate along an external magnetic field (Schüler et al., 1995). *C*_mag_ values at the end of bacterial growth were 1.82 ± 0.09 and 1.09 ± 0.01 for wild-type and mutant AMB-1 (three replicates for each strain), respectively, demonstrating a very limited orientation capability for the mutant strain.

### 3.4. Native Magnetic States of Wild-Type and Mutant AMB-1 Cells

**Figure 6.**
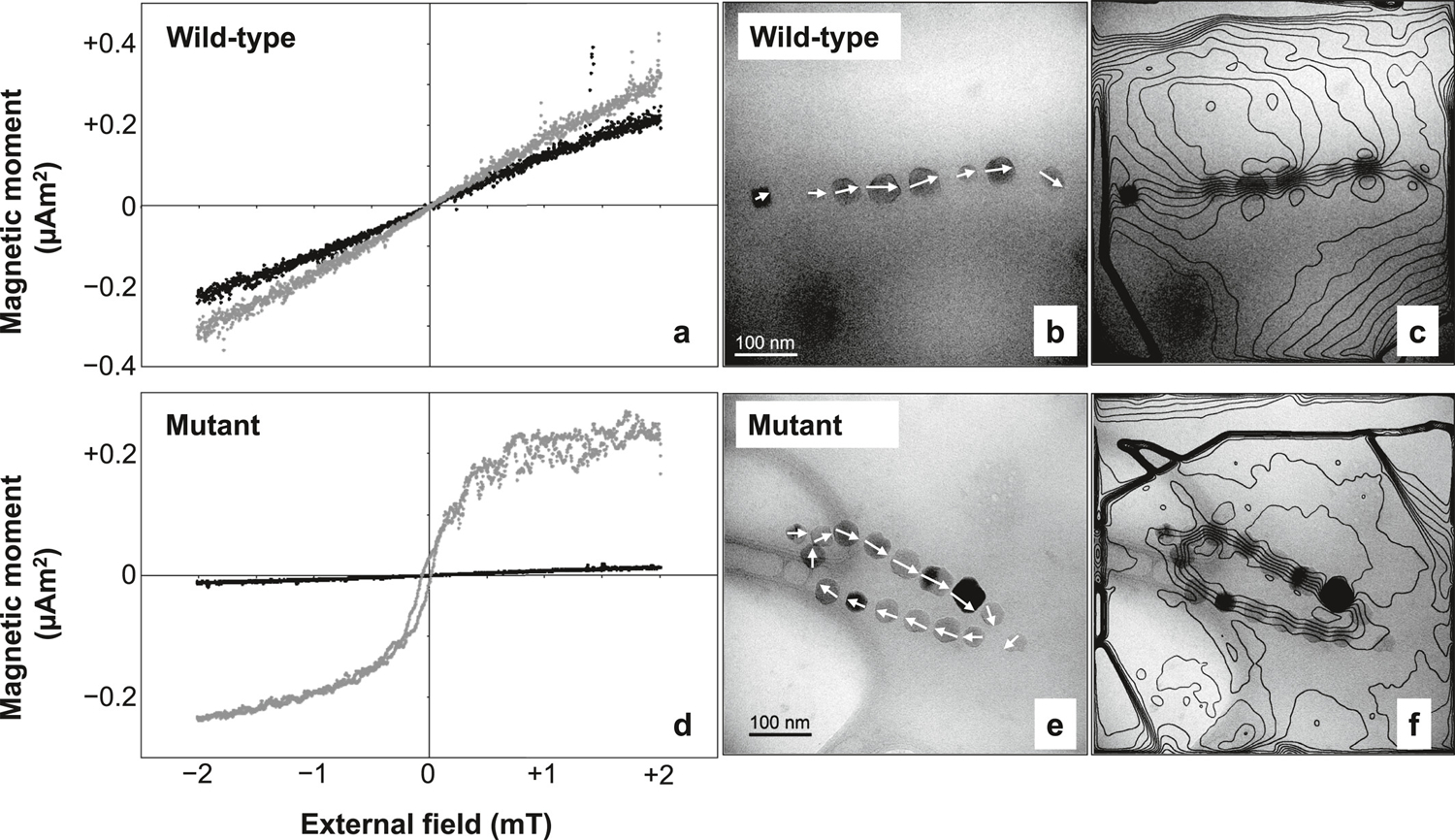
Native magnetic states of wild-type AMB-1 and the AMB-1 mutant. Left: magnetization of a suspension of (a) wild-type and (d) mutant AMB-1 cells in small fields varying between ±2 mT, before (black) and after (gray) saturation in a 200 mT field. Right: electron microscopy and corresponding magnetic phase contours determined by off-axis electron holography images of (b)–(c) wild-type and (e)–(f) mutant magnetite chains.

MTB cells containing ideal magnetite chains possess already a saturation moment. Exposing them to large fields will thus not change the magnetization of a cell suspension. In practice, a ~40% increase is observed for the wild-type AMB-1 after applying a 200-mT field (Figure 6a). This increase can be explained by the growth of aligned chain fragments with opposite native polarities within the same cell (Le Nagard et al., 2019). In this case, the application of a strong external field imparts the same polarity to all fragments, increasing the cell’s total magnetic moment. A drastically different behavior is observed with the AMB-1 mutant, where the application of a 200-mT field produces an 18-fold magnetization increase (Figure 6d). Along with the magnetic behavior assays (*C*_mag_), this result indicates a vanishingly small native magnetic moment of the AMB-1 mutant, which is compatible with a FC configuration of its looped chains. Overall, these results show that virtually all mutant cells contain looped magnetosome chains.

### 3.5. Electron Holography

Mapping of the magnetic flux in magnetosome chains produced by wild-type AMB-1 (Figures 6b and 6c) indicates that the magnetic moments of individual magnetosomes are nearly parallel to the chain axis, as previously observed (Dunin-Borkowski et al., 1998). In contrast, necklace structures of the mutant strain display a closed magnetic flux (Figures 6e and 6f). This configuration has a zero-net magnetic moment, up to small fluctuations due to asymmetries (e.g., larger crystals on one side of the structure), confirming the origin of the vanishing native magnetic moments of the AMB-1 mutant deduced from magnetic measurements.

### 3.6. FORC Measurements

**Figure 7.**
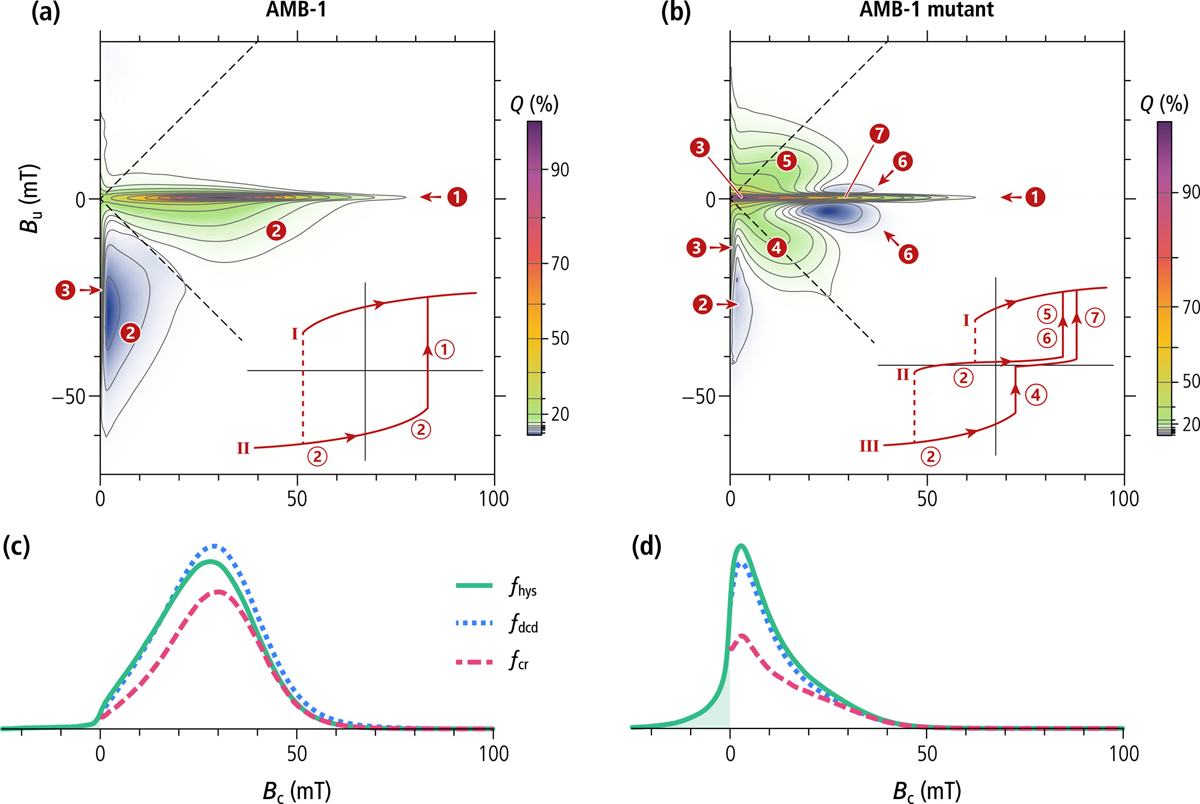
Magnetic properties of wild-type AMB-1 and the AMB-1 mutant. (a–b) FORC diagrams with 2%, 5%, 10%, 20%, 30%, 40%, 50%, 60%, 70%, 80%, 90% quantile contours. Insets are schematic representations of the magnetization of an individual cell as a function of the applied field. Numbers highlight the following features: 1—central ridge created by the magnetization jump through which the lowest curve merges with curve I; 2—positive and negative contributions from reversible magnetic moment rotation (difference between the slopes of consecutive curves); 3—signatures of magnetically viscous particles (vertical ridge and central ridge extending to the origin); 4—nucleation of a FC state from a negative SD state (contribution of the first magnetization jump in curve III to the difference between curves II and III); 5—annihilation of a FC state (contribution of the magnetization jump in curve II to the difference between curves I and II); 6—same as 5, but for the difference between curve II and III; 7—annihilation of a FC state (contribution of the second magnetization jump in curve III to the difference between curves II and III). (c–d) Coercivity distributions of wild-type AMB-1 and the AMB-1 mutant, obtained from subsets of FORC data: *f*_hys_—irreversible component of the ascending hysteresis branch, *f*_dcd_—DC demagnetization of *M*_rs_, *f*_cr_—central ridge. The shaded area represents the *f*_hys_ contribution of SD magnetic states that cannot exist in a null field.

The FORC diagrams of the wild-type and the mutant AMB-1 (Figure 7) share many similarities with the numerical simulations of single-stranded and double-stranded chains of equidimensional crystals, but also feature important differences. The central ridge of wild-type cells extends to the origin and is accompanied by a vertical ridge along *B*_c_ = 0, located mainly in the lower quadrant. These are the typical signatures of thermal relaxation in systems containing particles close to the lower SD stability limit (Lanci & Kent, 2018; Pike et al., 2001), and has been observed in the growing phase of MTB cultures (Carvallo et al., 2009). In case of single-stranded chains of equidimensional crystals, the lower SD stability limit is comprised between 12 and 17 nm (Newell, 2009) and is compatible with the smallest crystal sizes observed with TEM (Figure 3). The hysteresis squareness (*M*_rs_/*M*_s_ = 0.475) is slightly smaller than expected for single-stranded chains, probably because of a minor superparamagnetic contribution associated with immature chains, as confirmed by electron microscopy (Figure 3). The coercivity distributions obtained from FORC measurements of the wild-type cells (Figure 7c) are unimodal with a peak at 28–30 mT and the right tail extending to a maximum of 60–70 mT. Contrary to the micromagnetic simulations of single-stranded chains of equidimensional magnetosomes (Figure 5a) and to some examples of cultured MTB producing single-stranded chains (e.g., Y. Wang et al., 2015), the coercivity distributions are left-skewed and include a small, non-zero contribution at *B*_c_ = 0 caused by thermal relaxation.

The mutant AMB-1 strain shares the same FORC features (i.e., central ridge, viscous component, and rotations of magnetic moments in the applied field), albeit over different field ranges, but also exhibits additional contributions (Figure 7b), consisting of a doublet of positive lobes at (*B*_c_, *B*_u_) ≈ (12, ±8) mT, and a doublet of negative lobes at (*B*_c_, *B*_u_) ≈ (25, ±3) mT, almost symmetrically arranged above and below the central ridge. These lobes are the typical hallmark of FC nucleation and annihilation (Egli, 2021), as seen with VO particles (Dumas et al., 2007; Roberts et al., 2017) and small magnetosome clusters produced by the Δ*mamJ* mutant of MSR-1 (Katzmann et al., 2013). The hysteresis loop is constricted (Figure S8 in Supporting Information S1), and has a much smaller squareness (Table 1, Figure 1) caused by the instability of high-moment magnetic states predicted by micromagnetic simulations of magnetosome rings (Section 3.1). This instability is associated with FORC contributions extending beyond the memory region of the FORC diagram (Figure 7b, left of the dashed lines). Constricted hysteresis loops are typically the result of bimodal coercivity distributions, which, in this case, consist of a main peak at ~2 mT, and a shoulder at ~25 mT (Figure 7d). The main peak of the central ridge, close to the origin, is caused by thermal relaxation of high-moment states, which, as predicted by numerical simulations (Section 3.1), are unstable at room temperature. FC nucleation from the negative high-moment state (feature four in Figure 7b) contributes additionally to the main peak of the other two coercivity distributions, explaining their larger amplitude compared to the central ridge. A single mechanism, namely the annihilation of FC states around +25 mT, explains the existence of a shoulder with similar amplitude in all three coercivity distributions.

Overall, the bimodality of *f*_irr_ and *f*_dcd_ coercivity distributions is more subdued than expected from the existence of high-moment and low-moment states because thermal relaxations tend to shift these distributions toward the origin. Nevertheless, ~50% of the high-moment states in looped chains of the AMB-1 mutant possess a stable remanence, as indicated by the portion of the positive lobes above and below the central ridge located within the memory region (i.e., right of the dashed lines in Figure 7b). This is explainable by the stability of smaller loops with *N* ≤ 16 magnetosomes predicted by numerical simulations (Table 2).

## 4. Discussion

### 4.1. Distinctive Characteristics of the Magnetofossil Signature

Magnetic assemblages showing coexisting FORC signatures of UNISD particles (central ridge) and non-SD or interacting SD particles (positive contributions above and below the central ridge) were previously interpreted as mixings of several magnetite sources including intact and collapsed magnetofossil assemblages, and non-SD particles of detrital origin (Chang et al., 2019; Heslop, Roberts, & Chang, 2014; Lascu et al., 2015). However, mixings of the above-mentioned magnetic sources cannot provide a satisfactory explanation for the elevated hysteresis squareness typical of secondary magnetite in magnetofossil-rich sediments (Ludwig et al., 2013). Our work demonstrates that the magnetic signatures of these sediments can be explained by the sole presence of MTB producing single- and multi-stranded magnetosome chains, as well as specific alterations of these native configurations, such as fold-collapse, which maintain the original strong uniaxial anisotropy. Fold-collapsed chains form a uniform trend with the hysteresis properties of intact chains and UNISD particles (Figure 1), which is characterized by a very limited decrease of the hysteresis squareness (*M*_rs_/*M*_s_ ≥ 0.47), and *B*_cr_/*B*_c_ values comprised between ~1.2 and ~1.6, close to the limits for narrow and wide SD coercivity distributions, respectively (Dunlop, 2002).

Other putative chain collapse products, such as disordered magnetosome clusters and looped chains, might possess compatible FORC signatures, but lack the elevated hysteresis squareness of systems with stable SD-like remanent states. The hysteresis properties of large clusters of extracted magnetosomes (Kobayashi et al., 2006; Li et al., 2012) follow a trend characterized by a steep decrease of *M*_rs_/*M*_s_ (Figure 1) typical of densely packed synthetic SD magnetite particles (Muxworthy et al., 2003). This decrease is associated with a vertical widening of the FORC function beyond the limits of the memory region (L. Chen et al., 2007), due to large random internal fields associated with magnetostatic interactions (Muxworthy & Williams, 2005). FORC diagrams of SD particle clusters lack a central ridge (Carvallo et al., 2005); minor central ridge contributions in magnetosome extracts (e.g., Li et al., 2012) are therefore associated with incomplete chain collapse.

Micromagnetic simulations of magnetofossil collapse through chain axis randomization (Chang et al., 2019) produce a trend of hysteresis properties that is intermediate between the two cases described above (Figure 1). This trend, which includes the looped chains produced by our mutant AMB-1 as the endmember with lowest squareness, is associated with the intermediate dimensionality of chain loops (*D* ≈ 2) and of the partially collapsed chains generated by the random walk algorithm of Harrison and Lascu (2014) (1 < *D* < 2), compared to intact or fold-collapsed chains (*D* ≈ 1) and dense clusters (*D* ≈ 3), respectively. FORC diagrams with non-central ridge contributions and hysteresis loops with *M*_rs_/*M*_s_ ≈ 0.5 are therefore important hallmarks of SD particle systems with a uniaxial anisotropy that is sufficiently strong to stabilize SD-like remanent states. The only known natural way for secondary SD magnetite particles to develop such a strong uniaxiality is by inheritance of native magnetosome chain structures, which must therefore survive diagenesis without becoming fully randomized. Fold-collapse is one of the possible fates of single-stranded chains that are not immediately stabilized by electrostatic adhesion to other sediment particles. Multi-stranded chains, on the other hand, are expected to be more resistant to mechanical solicitations, due to the staggered magnetosome arrangement, and might survive diagenesis with little structural modifications.

A bell-shaped central ridge coercivity distribution is the second distinctive element of magnetofossils. It is generated by the denucleation of magnetic states with largest switching field: specifically, the high-moment, SD-like state of single-stranded chains, and the low-moment, FC state of multi-stranded chains. Our simulations show that the switching fields of these states tend to form two relatively narrow distributions with medians of ~40–50 mT in the case of equidimensional magnetosomes and ~70–80 mT in the case of prismatic magnetosomes, before maghemitization. These distributions are similar to the biogenic components BS and BH discovered in AF demagnetization curves of ARM (Egli, 2004a) and later identified also in the central ridge of magnetofossil-rich sediments (Heslop, Roberts, & Chang, 2014; Ludwig et al., 2013). The existence of a minimum uniaxial anisotropy provided by the chain structure ensures that the coercivity distributions of BS and BH are inferiorly limited by a non-zero switching field of ~10 mT (BS) and ~25 mT (BH). This limit is absent in isolated SD particles and in interacting clusters of such particles (Egli, 2021). An interesting counterexample is provided by a mutant MTB producing widely spaced magnetosomes (X. Wang et al., 2019): in this case, the combined action of magnetic viscosity and lack of strong in-chain magnetostatic interactions shifts the central ridge distribution peak toward *B*_c_ = 0.

ARM and the central ridge are expected to have different sensitivities toward single- and double-stranded chains. In case of single-stranded chains, ARM represents the room-temperature thermodynamic equilibrium between two symmetric SD states in the decaying AF field, like for UNISD particles (Egli & Lowrie, 2002). The case of systems featuring two symmetric SD-like states with denucleation field *B*_n1_ and two symmetric FC states with higher denucleation field *B*_n2_, as in double-stranded and fold-collapsed chains, is more complex: once the AF field has decayed below *B*_n2_ during ARM acquisition, the system becomes trapped into one of the two FC states. In this case, the acquired ARM is the room-temperature thermodynamic equilibrium between two symmetric FC states. This ARM is reset to zero only in AF fields with initial amplitude ≳*B*_n2_. The similarity between *B*_n2_ and the switching field of single-stranded chains made of the same type of magnetosomes (Figure 5), yields comparable ARM and central ridge coercivity distributions. However, while the central ridge response is proportional to the mean amplitude of the magnetization jumps associated with FC denucleation, which amounts to ~40% of the saturation moment (*M*_cr_/*M*_s_ in Table 1), the ARM response is proportional to the remanent moment of FC states, which, in case of identical strands, is nearly zero. Therefore, the ARM of double stranded chains reflects the residual net magnetic moment of asymmetric strands, which is significantly smaller than the saturation moment. FC states are therefore expected to lower the ratio *χ*_a_/*M*_rs_ between the ARM susceptibility and the saturation remanence. A marked decrease of *χ*_a_/*M*_rs_ has been observed for the BS and BH components of lake sediments during a eutrophication event and was tentatively attributed to the presence of greigite magnetofossils (Egli, 2004a, 2004b). However, greigite-producing MTB appear to have similar magnetic signatures as magnetite-producing MTB (A. P. Chen et al., 2014), so that multistranded chains are the most likely explanation for *χ*_a_/*M*_rs_ variations of magnetofossil components. Indeed, *χ*_a_/*M*_rs_ values varying between ~1.3 and ~1.7 mm/A inside a magnetofossil-rich sediment core from the western equatorial Pacific have been found to be uncorrelated to a residual detrital component, but positively correlated with a FORC-PCA principal component that is sensitive to the relative contribution of the central ridge to the total FORC magnetization (Inoue et al., 2021). For comparison, our micromagnetic simulations predict relative central ridge contributions of >0.91 and <0.57 for single-stranded and double stranded chains, respectively (Table 3).

Coercivity distributions obtained from other types of magnetization curves, such as the commonly employed isothermal remanent magnetization (IRM) acquisition (Kruiver et al., 2001), contain contributions from all transitions between magnetic states, which, in the case of multi-stranded chains, include the denucleation of SD-like states in the field *B*_n1_. This additional contribution is easily identifiable as a secondary low-coercivity peak in the case of fold-collapsed chains of equidimensional magnetosomes (Figure 5c), while it merges with the distribution of *B*_n2_ in the other cases, lowering the median field. As a result, coercivity distributions obtained from IRM acquisition curves respond to a larger number of transitions between magnetic states than the central ridge or ARM, yielding less constrained coercivity components that make the identification of BS and BH more difficult or even impossible. More importantly, these coercivity distributions respond also to unstable remanent magnetic states that are switched in arbitrarily small fields, which means that they need to be described by left-skewed functions with non-zero contributions at *B*_c_ = 0 (Egli, 2003; Zhao et al., 2018).

### 4.2. Forward Models of Magnetofossil FORC Signatures

The FORC signature of single-stranded chains is identical to that of UNISD particles: both systems possess only a pair of antisymmetric SD-like states, and the in-field transition between these states produces the central ridge. The transition is preceded by a reversible rotation of magnetic moments, which generates additional FORC contributions in the lower quadrant. The coercivity distributions *f*_hys_ and *f*_cr_ associated with in-field magnetization jumps produced by the switching between SD-like states are identical and proportional to the coercivity distribution derived from remanent magnetization curves (e.g., *f*_dcd_). The small differences between *f*_hys_ and *f*_cr_ in Figures 5a and 5d are due to the numerical method used to isolate the central ridge from other FORC contributions. The difference in amplitude between remanent and in-field coercivity distributions is quantified by *M*_cr_/*M*_rs_, where *M*_cr_ is the magnetization associated with the central ridge (Egli et al., 2010). Our micromagnetic simulations yield *M*_cr_/*M*_rs_ ≈ 0.704 and 0.711 for single-stranded chains of equidimensional and prismatic magnetosomes, respectively, compared to 0.544 for the Stoner-Wohlfarth model (Egli et al., 2010), whereby all these estimates do not take thermal activations into account. The higher *M*_cr_/*M*_rs_ values of single-stranded chains are explainable by the fact that reversible magnetic moment rotations are limited mostly to the end magnetosomes, while central magnetosomes tend to maintain a better alignment with the chain axis. Non-central ridge contributions in the lower quadrant of the FORC diagram are perfectly antisymmetric with respect to *B*_u_ = −*B*_c_ and do not contribute to the total magnetization *M*_forc_ obtained by integrating the FORC function, so that *M*_forc_ = *M*_cr_.

**Figure 8.**
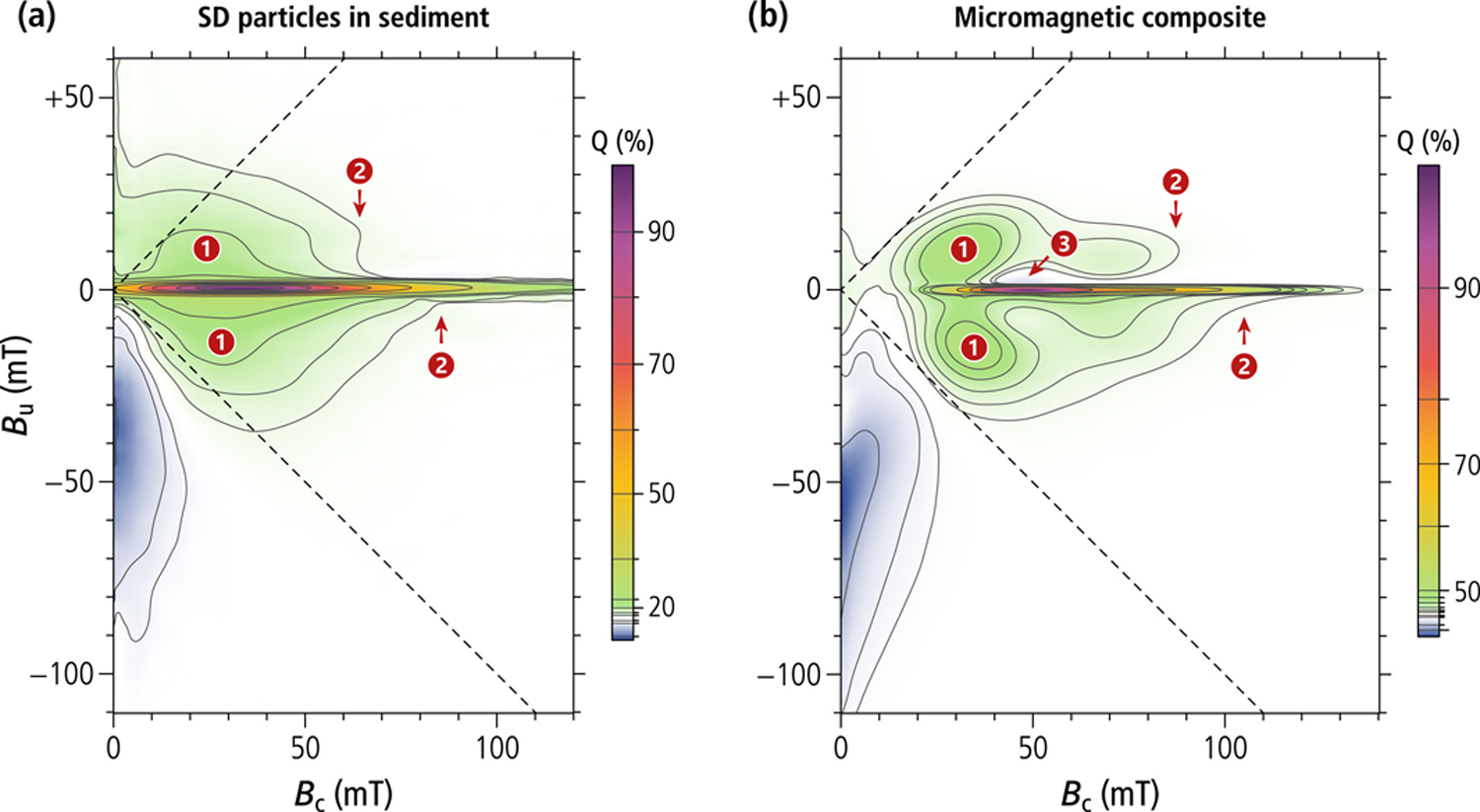
Comparison between the FORC signature of SD particles in a magnetofossil-rich sediment and a simulated magnetofossil composite. (a) FORC diagram of in-situ secondary magnetite particles (mostly magnetofossils) in a pelagic sediment (data from Ludwig et al., 2013). (b) FORC diagram of a simulated composite obtained from the six micromagnetically modeled chain structures, with the following relative contributions to the saturation magnetization, chosen for a visual match with the FORC diagram in (a): 1/3 single-stranded chains of equant magnetosomes, 1/6 single-stranded chains of prismatic magnetosomes, 1/3 native double-stranded chains of equant magnetosomes, 1/6 native double-stranded chains of prismatic magnetosomes, 1/3 fold-collapsed chains of equant magnetosomes, 1/6 fold-collapsed chains of prismatic magnetosomes. The same field range and quantile contour levels are used in both plots to ease the comparison. Numbers highlight the following features: 1—nucleation/annihilation of FC states, 2—high-field limit for the nucleation/annihilation of a FC state (asymmetric about the central ridge), 3—residual negative amplitude from the annihilation of positive FC states, uncompensated by positive amplitudes from intermediate coercivity contributions.

The FORC diagrams of double-stranded and fold-collapsed chains contain additional non-central ridge contributions originating from transitions between high- and low-moment magnetic states. These contributions create nearly symmetric FORC amplitudes around the central ridge, which contribute to *M*_rs_ and *M*_forc_, but not to *M*_cr_. The corresponding ratios *M*_cr_/*M*_forc_ ≈ 0.43–0.57 and *M*_cr_/*M*_rs_ ≈ 0.38–0.42 are therefore significantly smaller than those of single-stranded chains (Table 3). For comparison, the FORC signature of secondary magnetite particles isolated from a magnetofossil-rich sediment (Ludwig et al., 2013) is characterized by values of *M*_cr_/*M*_forc_ and *M*_cr_/*M*_s_ that are intermediate between those of our simulated single-stranded and double-stranded or fold-collapsed chains. Mixtures of nearly equal parts of single-stranded and double-stranded chains (native and/or fold-collapsed) yield magnetization ratios that are compatible with those of magnetofossils (Table 3). Therefore, a composite FORC diagram has been generated using a mixture of equal amounts of all chain configurations simulated in this work, assuming a 2:1 proportion of equidimensional and prismatic magnetosomes (Figure 8b). This proportion has been chosen to best reproduce the magnetofossil signature isolated from a pelagic carbonate through selective chemical dissolution of exposed (i.e., not in silicate inclusions) <0.3 μm magnetite particles (Figure 8a). This example probably represents the best available reconstruction of the FORC signature of secondary magnetite particles in a magnetofossil-rich sediment. As discussed in Ludwig et al. (2013), temperature-controlled variations of the leaching effectivity yielded identical in-situ magnetic properties of the dissolved fraction between 40°C and 60°C: this is possible only if (a) there is a gap between the grain size distributions of secondary (leachable) and primary (non-leachable) particles, and (b) the upper size limit for full dissolution falls within this gap. The corresponding FORC diagrams share striking similarities, but also some important differences. Both diagrams feature a central ridge; however, the coercivity range of simulations is comprised between ~20 and ~130 mT, while the natural central ridge covers also the 0–20 mT range (see Section 4.4).

Other signatures of our composite FORC diagram are qualitatively similar to those of magnetofossil-bearing sediments, including the slight asymmetry between the upper and the lower quadrant, and the abrupt *B*_c_-termination of FC contributions in the upper quadrant. The contour lines of the FORC simulation are clearly affected by localized contributions from FC nucleation and annihilation of the four double-chain configurations, which are not completely merged into a smooth distribution as in the natural counterpart. Small negative amplitudes above the central ridge are also not completely canceled. This is understandable considering that simulations were limited to few, well-defined geometries. For instance, single- and multi-stranded chains made of bullet- or tooth-shaped magnetosomes were not included. Multi-stranded chains of bullet-shaped magnetosomes are characterized by similarly elevated hysteresis squareness values as our double-stranded simulations, with a central ridge peaking at ~60 mT (Li et al., 2010). This intermediate coercivity range fills the gaps between simulated chains of equidimensional and prismatic magnetosomes, making the resulting FORC diagram more similar to that of natural magnetofossils.

### 4.3. Magnetofossil Fingerprints and Numerical Unmixing

Our numerical simulations show that magnetofossils possess a unique combination of magnetic properties related to the preservation of native or collapsed chain structures with a strong uniaxial anisotropy. This uniaxial anisotropy is at the basis of all magnetic criteria proposed so far for magnetofossil identification, which include (a) the central ridge (Egli et al., 2010; Heslop, Roberts, & Chang, 2014), (b) relatively narrow coercivity components not extending to *B*_c_ = 0 (Egli, 2004a), and (c) ferromagnetic resonance (FMR) spectra with positive anisotropy (Charilaou et al., 2011; Kind et al., 2011; Kopp et al., 2006). An additional criterion discussed in this work is the confinement of FORC contributions within the memory region, which ensures that *M*_rs_/*M*_s_ ≈ 0.5 also in case of multi-stranded and fold-collapsed chains. Each of these criteria, if taken singularly, is fulfilled by an equivalent assemblage of isolated SD particles with appropriated equivalent anisotropy, but all known natural examples other than magnetofossils do not fulfill all of them. For instance, titanomagnetite needles in the Tiva Canyon Tuff do possess a central ridge (Berndt et al., 2018), but the associated coercivity distribution and vertical offset are different from those of magnetofossils. In another example (Till et al., 2017), magnetite derived from the inorganic breakdown of nanocrystalline goethite produces FORC diagrams similar to those of magnetofossil-rich sediments, but with a much lower hysteresis squareness. Finally, SD magnetite can form along dislocation lines of silicate minerals, forming “strings of pearls” as documented in ancient zircons (Tang et al., 2019); however, the large irregular particle spacing resulting from this growth process is not expected to yield the narrow coercivity distribution of single-stranded chains.

Because natural sediments contain mixtures of magnetite particles with different primary and secondary origins (e.g., Franke et al., 2007; Just et al., 2012; Li et al., 2020), magnetofossil signatures need to be isolated using numerical unmixing methods. Numerical unmixing methods can be divided into parametric ones, which are based on model properties, such as coercivity distribution functions (Egli, 2003; Kruiver et al., 2001), and non-parametric ones, which are fundamentally linked to PCA, such as non-negative unmixing of remanent magnetization curves (Heslop & Dillon, 2007) and FORC-PCA (Harrison et al., 2018; Lascu et al., 2015). Parametric coercivity analyses are very sensitive to noise and to subtle variations of the shape (skewness) of individual coercivity distributions (Egli, 2003; Zhao et al., 2018). Furthermore, individual coercivity components might be associated with irreversible magnetization changes induced by a specific measurement protocol, rather than distinct types of magnetic particles. This is particularly evident in the case of fold-collapsed chains (Figure 5c), where *f*_cr_ is described by a single unimodal distribution, while the other coercivity distributions are bimodal and require two coercivity components for a correct fit. In this case, the analysis of magnetization curves that are sensitive only to certain transitions between magnetic states, such as the central ridge and the AF demagnetization of ARM, helps with the identification of magnetofossil-specific coercivity components.

The correct individuation of endmembers in PCA-based unmixing methods is based on the assumption that individual magnetic components possess fixed magnetic properties within the set of analyzed samples. In this case, a single magnetofossil endmember is expected from PCA analyses. More than one endmember is needed to describe magnetofossil signatures that change in time, reflecting, for instance, variable proportions of equidimensional and elongated crystals (Hesse, 1994; Wagner et al., 2021; Yamazaki & Kawahata, 1998), or single-stranded and multi-stranded chains.

Our micromagnetic simulations suggest that magnetosome elongation would control mainly the coercivity of magnetofossil endmembers (e.g., the proportion of BS and BH coercivity components), while higher proportions of multi-stranded chains would increase non-central ridge contributions to the FORC diagram. In the case of FORC-PCA analyses, this requires two principal components sensitive to the amplitude and the coercivity of the central ridge, respectively, as observed in a magnetofossil-rich sediment core (Figure 8 in Inoue et al., 2021), and the space defined by these two principal components is spanned by at least three magnetofossil-related endmembers. Splitting between these endmembers is controlled by the PCA covariance matrix, rather than by physical parameters. For instance, variable proportions of single-stranded and multi-stranded chains could be described by the linear combination of a pure central ridge and a pure non-central ridge endmember, instead of the mixed FORC signatures of single- and multi-stranded chains. This might explain FORC-PCA results from the North Atlantic (Channell et al., 2016), where the three identified endmembers include one with the typical FORC signature of detrital magnetite (EM three in Figure 11 of that study), one with a typical UNISD signature consisting of a central ridge and almost no contributions in the upper quadrant (EM2, ibid), and one that is compatible with non-central ridge contributions from SV magnetite particles or multi-stranded magnetosome chains (EM3, ibid). In this example, the interpretation of the latter endmember is ambiguous; especially when considering that PCA can group the signatures of magnetic components with completely different origins–such as multi-stranded magnetofossils and a fine detrital component SV signature–into a single endmember, if these components co-vary in time. Co-varying concentrations of primary and secondary magnetite particles can be expected if the abundance of magnetofossils is controlled by the flux of nutrients, which, in certain locations, is in turn controlled by primary mineral inputs (e.g., Roberts et al., 2011). Endmembers with characteristics similar to those of the above example are commonly found in marine sediments (Inoue et al., 2021; Lascu et al., 2015; Yamazaki et al., 2020). FORC-PCA results are also sensitive to the number of principal components and the choice of the endmembers: for example, the FORC signature of a fourth endmember featuring two lobes around the central ridge like those of our micromagnetic simulations of double-stranded chains is distributed among the other three endmembers in the case of a three-endmember analysis of the same samples (Figures 9 and 10 in Lascu et al., 2015).

The ambiguity of numerical unmixing results can be partially overcome by combining numerical methods with particle morphology statistics from TEM observations (Wagner et al., 2021) and with physical or chemical separation techniques, such as the selective dissolution of fine magnetite particles (Ludwig et al., 2013).

### 4.4. Authigenic Magnetite in Magnetofossil Signatures?

Fe(III)-reducing and Fe(II)-oxidizing bacteria are known to induce the precipitation of magnetite particles that can contribute to the magnetic signature of sediments affected by iron diagenesis. Ultrafine magnetite particles with broad volume distribution and irregular shapes have been often observed (e.g., Gibbs-Eggar et al., 1999; Franke et al., 2007; Li et al., 2020; Oldfield, 2007), but their magnetic fingerprint is uncertain, owing to the uncontrolled grain size distribution and the unknown arrangement in the sediment matrix. Magnetite samples produced by these bacteria in the laboratory (Table 1) are characterized by a small hysteresis squareness (Carvallo et al., 2008; Moskowitz et al., 1993) and the classical FORC signature of strong magnetostatic interactions (Miot et al., 2014). Because of size distribution overlapping with the stable SD range, authigenic magnetite has a similar preservation potential in geological materials (Roberts et al., 2012) and a magnetic signature that might be confused with that of magnetofossils. Irregularly shaped SD magnetite particles lacking a source of minimum uniaxial anisotropy, for instance by particle elongation or chain arrangement, are expected to produce a broad coercivity distribution with a finite contribution at *B* = 0 and median fields of 10–25 mT (Moskowitz et al., 1993). Coercivity components with similar characteristics have been found in freshwater and marine sediments, as well as in soils (EX and P in Egli, 2004a). These components are characterized by moderately large *χ*_a_/*M*_rs_ values typical of non-interacting SD particles (Egli, 2004a), and, in the case of soils, by a central ridge (Egli, 2021; Geiss et al., 2008). Therefore, authigenic magnetite consists, at least in part, of isolated SD particles dispersed in the sediment matrix. The broad grain size distribution of biologically uncontrolled magnetite precipitation (e.g., Liu et al., 2005), which includes SV particles, is expected to yield also FORC signatures typical of non-SD particles, as indeed observed in FORC diagrams of well-developed soils (Egli, 2021). Isolated SD magnetite particles with random departures from equidimensional shapes also produce low-coercivity FORC contributions above and below the central ridge (e.g., Figure 51 in Egli, 2021), due to the competition between magnetocrystalline and shape anisotropies. Finally, elevated local iron concentrations might lead to the formation of particle clusters with strong magnetostatic interactions that contribute to the vertical dispersion of the FORC function at low coercivities. Indeed, magnetite encrustations of Fe(III)-reducing or Fe(II)-oxidizing bacteria have been observed in culture (e.g., Miot et al., 2009). For instance, FORC signatures of non-interacting and interacting SD particles not produced by MTB have been identified in organic-rich methanogenic sediments (Ustra et al., 2021). Both non-SD and clustered SD magnetite contributions are expected to lower the hysteresis squareness and might therefore be distinguishable from pure magnetofossil contributions.

Overall, the SD fraction of well-dispersed authigenic magnetite particles can produce magnetic signatures that might merge continuously with those of magnetofossils over the low-coercivity range. For instance, the low-coercivity FORC-PCA endmembers EM1 and EM2 in red clays from the Pacific Ocean (Yamazaki et al., 2020) provide altogether a signature that is similar to that of the pedogenic enhancement in soils. A low-coercivity component peaking at *B*_c_ = 0 is also particularly evident in the central ridge of magnetofossil-bearing ferromanganese crusts formed mainly through biomineralization (Jiang et al., 2020), while being absent in other crusts (Oda et al., 2018). The interpretation of the low-coercivity range (<20 mT) of the central ridge remains uncertain. Short-chain fragments of 1–3 equant magnetosomes possess significantly lower coercivities than fully developed chains (Chang et al., 2019) and can therefore contribute to the central ridge over this range. It is not known whether such fragments are produced by MTB under natural, slow-growing conditions, or if longer chains are naturally fragmented through adhesion of segments of the same chain to different sediment particles. Overall, the existence of low-coercivity contributions in the central ridge of most sediments means that a biogenic and an inorganic origin of SD magnetite particles, as postulated by different authors for the Paleocene-Eocene Thermal Maximum, are not mutually exclusive (Kent et al., 2003; Lippert & Zachos, 2007).

### 4.5. Paleontological and (Paleo)Environmental Implications

Discrimination of single-stranded and multi-stranded chains based on the FORC signatures discussed above are of paleontological interest and could provide a temporal resolution on the evolution of magnetosome formation in MTB. Only bacteria belonging to the ancestral Delta-proteobacteria and Nitrospirae phyla were thought to produce multi-stranded chains of magnetosomes (Deng et al., 2016). However, they have now been identified in the more recent Alpha-proteobacteria and Gamma-proteobacteria phyla (Taoka et al., 2014; Zhang et al., 2017). Therefore, multi-stranded chain configuration has no specific phylogenetic distribution and might instead correspond to an adaptation to specific environments. For instance, single-stranded chain-producing cocci and the multistranded *M. bavaricum* display different responses to a sudden change of the oxygen gradient in sediment (Mao et al., 2014). In MTB, magnetosomes are aligned along cytoskeletal filaments made of actin-like proteins (Müller et al., 2020). Among them, the protein MamK is ubiquitously conserved in all known MTB. Bacteria forming multiple magnetosome chains contain several copies of genes encoding actin-like proteins in addition to MamK (Kolinko et al., 2016). We can thus hypothesize that genetic factors are responsible for multi-stranded chain configurations (Kolinko et al., 2016). They may include gene duplication events (Murat et al., 2010), or horizontal gene transfers (i.e., an exchange of genetic material through direct physical interactions between two organisms) (Monteil et al., 2020). Once the genetic basis of chain configurations (Du et al., 2019) is fully understood, the methodology we describe in this work can be used to provide a temporal constrain on evolutionary events that occurred in one of the oldest and more diverse group of biomineralizing organisms.

### 4.6. Paleomagnetic Implications

Owing to the widespread occurrence of living MTB in the topmost ~10–20 cm of the sedimentary column (Flies et al., 2005; Mao et al., 2014; Petermann & Bleil, 1993) and cell dissolution in the same layer, magnetofossils are expected to contribute to sedimentary records of the Earth magnetic field like other magnetic particles. However, specific differences between the magnetic and mechanical properties of magnetosome chains and other carriers of a natural remanent magnetization (NRM) might affect relative paleointensity records (RPI) of magnetofossil-rich sediments (A. P. Chen et al., 2017; Inoue et al., 2021; Larrasoaña et al., 2014; Ouyang et al., 2014). Although the exact mechanism of NRM acquisition is unknown, it is possible to make some predictions about the influence of chain geometry on the RPI efficiency of magnetofossils on the basis of our findings.

Magnetofossils in non-varved sediments are expected to form close to the depth range inhabited by living MTB (Petermann & Bleil, 1993), inside the surface mixed layer (SML). In this case, magnetofossils would undergo the same continuous reorientation process of other remanence carriers, until they are definitively buried below the SML. The resulting NRM has been described as the product of a dynamic equilibrium between (a) the aligning action of the Earth magnetic field on the net natural magnetic moment of isolated or grouped magnetic particles behaving as rigid mechanical units (e.g., clusters or inclusions in silicate minerals), and (b) the rotational diffusion associated with sediment mixing (Egli & Zhao, 2015; Zhao et al., 2016). In this case, the resulting remanent magnetization *M*_nrm_ = *M*_0_ *S*(*m*_0_*B*/*τ*_s_) is proportional to the total magnetization *M*_0_ of fully aligned remanence carriers, and to a sigmoidal function *S*, with *S*(0) = 0 and *S*(∞) = 1, which describes the partial alignment of remanence carriers in the Earth magnetic field *B* (Egli & Zhao, 2015). In small fields, this partial alignment is proportional to the ratio between the magnetic torque *m*_0_*B* acting on individual remanence carriers with natural magnetic moment *m*_0_, and the mean holding torque *τ*_s_ that resists particle reorientation. Writing *M*_0_ = *cm*_0_, where *c* is the concentration of remanence carriers, gives $M_{\text{nrm}}=cm_{0}^{2}\kappa_{\text{nrm}}$ for the low-field limit of NRM acquisition, with $K_{\text{nrm}}\propto \tau_{\text{S}}^{-1}$ being a coefficient that describes the sediment-specific mechanical alignment efficiency of the remanence carriers.

RPI records are obtained by normalizing *M*_nrm_ with a laboratory magnetization *M*_lab_ assumed to be proportional to *M*_0_. In this case, *M*_lab_ = *c m*_lab_, with *m*_lab_ being the mean magnetic moment component along the applied magnetic field, acquired by the remanence carriers during the acquisition of a laboratory magnetization. The normalized NRM is finally given by $R=\kappa_{\text{nrm}}m_{0}^{2}/m_{\text{lab}}B$. As expected for valid RPI normalizations, *R* does not depend on the concentration of remanence carriers. Furthermore, a fixed magnetic mineralogy yields a constant $m_{0}^{2}/m_{\text{lab}}$, so that *R* is proportional to *B*.

Each magnetic component contributing to the NRM is expected to possess a specific $m_{0}^{2}/m_{\text{lab}}$ value related to its magnetic properties, and a specific *κ*_nrm_ that reflects its mechanical properties. Accordingly, sediments containing variable proportions of two or more magnetic components (e.g., detrital and biogenic), yield RPI records that contain an unwanted environmental modulation caused by variations of $\kappa_{\text{nrm}}m_{0}^{2}/m_{\text{lab}}$ within the limit values of individual components (Fabian & Leonhardt, 2009). The role of $m_{0}^{2}/m_{\text{lab}}$ can be investigated by inspecting selected categories of remanence carriers. For instance, the natural magnetic moment of silicate minerals containing *n* ≲ 50 SD magnetic inclusions is proportional to the sum of *n* randomly oriented vectors, which yields $m_{0}\approx m_{\text{SD}}\sqrt{8n/3\pi }$, where *m*_SD_ is the mean magnetic moment of individual SD particles (Heslop, Roberts, & Hawkins, 2014). For comparison, intact chains of *n* magnetosomes are characterized by *m*_0_ = *m*_SD_*n*, while fold-collapsed chains have a much smaller native moment *m*_0_ = *m*_SD_δ*n*, where δ*n* is the mean absolute difference between the number of magnetosomes in the two strands (e.g., δ*n* = 0 for a chain that is folded exactly in the middle). A similar reasoning can be applied to the estimation of magnetic moments produced by the ARM and IRM commonly used as RPI normalizers (Table 4).

Magnetofossil RPI estimates obtained with the above NRM acquisition model display different degrees of sensitivity to the chain structure. If IRM is used as normalizer, similar RPI values are expected for intact single- and multistranded chains, since they carry a saturated magnetic moment in their native state and after application of a saturating IRM. Fold-collapsed chains, on the other hand, have a small native moment, making them inefficient NRM carriers. If ARM is used as a normalizer, intact double-stranded chains are expected to yield larger RPI values than intact single-stranded chains, because the ARM sets multistranded chains in an FC state with low magnetic moment (see Section 4.1). The weak negative correlation between *R* and *χ*_a_/*M*_rs_ in magnetofossil-rich sediments (Inoue et al., 2021) suggests that magnetofossils contain variable amounts of single- and multi-stranded chains, rather than collapsed chains: this is because collapsed chains would instead yield a positive correlation between NRM/IRM and *χ*_a_/*M*_rs_. In case of comparable mechanical alignment efficiencies (*κ*_nrm_), intact magnetofossils are expected to possess larger RPI efficiencies than silicate minerals containing SD magnetite inclusions with the same saturation moment, as reported by Ouyang et al. (2014) and A. P. Chen et al. (2017), because the magnetic moments of individual magnetosomes add arithmetically to the native magnetic moment, instead of randomly. The alignment efficiency of magnetofossils, on the other hand, is expected to depend strongly on the type of sediment particles onto which they might adhere.

## 5. Conclusions

Rock and sediment magnetism methodologies have been extensively used for the search and identification of magnetofossils. The central ridge observed in FORC analyses was established as a distinctive feature of the strong uniaxial anisotropy of isolated magnetosome chains. Our findings show that intact multi-stranded and fold-collapsed chains can generate additional signatures in FORC diagrams through the nucleation and annihilation of FC magnetic states while maintaining elevated hysteresis squareness (*M*_rs_/*M*_s_) values. FC magnetic states in multi-stranded chains, which can be described by individual strands possessing opposed magnetic polarities, are similar to FC states observed in small magnetosome clusters and looped magnetosome chains forming after cell dissolution in aqueous solutions, which might serve as term of comparison for diagenetic chain collapse in sediment. However, these artificial magnetosome structures are characterized by a significantly lowered hysteresis squareness that is not observed in magnetofossil-rich sediments. Micromagnetic calculations show that native multi-stranded chains and fold-collapsed chains feature stable SD states with large magnetic moments and the same hysteresis squareness of intact single-stranded chains. This stability, which has been confirmed experimentally in the case of native chains (Hanzlik et al., 2002; Thomas et al., 2008), originates from the strong uniaxial anisotropy of such structures and explains the coexistence of (a) magnetic signatures typical for systems of isolated SD particles and single-stranded magnetosome chains (central ridge and *M*_rs_/*M*_s_ ≈ 0.5), and (b) signatures typical of SD particle systems with random magnetostatic interactions (non-central-ridge contributions in the FORC diagram).

Full chain collapse leading to a complete loss of the original structure is likely prevented by the electrostatic adhesion of magnetosomes to sediment particles. Therefore, the strong uniaxial anisotropy of intact and fold-collapsed magnetosome chains appears to be a key feature for the preservation of a unique magnetic signature of magnetofossils, which, besides the elevated hysteresis squareness and a central ridge, includes two coercivity components (BS and BH in Egli, 2004a) with inferiorly limited, Gaussian-like switching field distributions distinct from those of isolated or clustered SD magnetite particles. Our micromagnetic models suggest that the low-coercivity portion of the central ridge of natural sediments (Figure 8) likely represents the contribution of isolated SD particles of authigenic origin. ARM is another key parameter of magnetofossil fingerprints: its coercivity distribution is similar to that of the central ridge, but, unlike to the central ridge, it is more selective toward single-stranded chains.

Preservation of a distinctive magnetofossil fingerprint, as demonstrated in our work, is pivotal for the interpretation of the origin of secondary SD magnetite particles in sediments, since this fingerprint is the only practical mean for verifying, in a quantitative manner, the existence of isolated chain-like structures of SD magnetite particles as the most essential magnetofossil identification criterion. The detailed characteristics of magnetofossil fingerprints depend on the relative abundances of equidimensional and elongated magnetosomes, which affect the coercivity distribution, and on the relative abundances of single-stranded and multi-stranded chains, which affect the fraction of magnetic remanence carried by the central ridge. Accordingly, two principal components might be required to describe the FORC properties of natural sediments with variable magnetosome shapes and chain structures. Magnetosome shape and chain structure reflect environmental conditions controlling the growth of different MTB strains. Therefore, detailed magnetofossil records obtained with high-resolution FORC measurements, combined with statistically significant TEM observations, will provide novel methodologies to discriminate distinct populations of ancient MTB and improve our understanding of paleomagnetic and environmental records of magnetofossil-rich sediments.

## Supplementary Material

Supplementary Materials

Supplementary Movie 1

Supplementary Movie 4

Supplementary Movie 3

Supplementary Movie 2

## Figures and Tables

**Table 1:** *Summary of Measured and Modeled Magnetic Properties of MTB, Magnetofossils, and Other SD Particle Assemblages, From This Study and From the Literature. M*_ts_ / *M*_s_ *Is The Ratio Between the Saturation Remanence and the Saturation Magnetization, χ*_a_ / *M*_rs_ *is the So-Called ARM Ratio, and M*_cr_/ *M*_rs_ *the Ratio Between the Total Magnetization of the Central Ridge and the Saturation Remanence*

Structure	References	*M*_rs_/ *M*_s_	*χ*_a_/ *M*_rs_ (mm/A)	*M*_cr_/ *M*_rs_

Natural sediment				
Lake Baldeggersee	Egli (2004a), Wagner et al. (2021)	0.41	2–4	0.37
Lake Ely	Egli et al. (2010)	0.47	–	0.48
Pelagic carbonate (CBD)^a^	Ludwig et al. (2013)	0.47	2–3	0.65
Paleosol S1 (CBD)^a^	Egli (2004a, 2021)	0.20	1.7	0.32
Single-stranded chains				
Cultured MTB	Li et al. (2012), Moskowitz et al. (1993)	0.50	3.1–3.7	0.65
AMB-1	This study, Li et al. (2012)	0.48–0.50	1.38	0.79
Simulated	This study	0.49–0.50	–	0.71–0.72
Simulated	Chang et al. (2019)	0.32–0.50	–	–
Multi-stranded chains				
Wild-type MTB	Pan et al. (2005)	0.47–0.51	0.5–0.8	–
Simulated (two strands)	This study	0.47–0.49	–	0.40–0.42
Simulated (fold-collapsed)	This study	0.47–0.50	–	0.37–0.38
Clumps and loops				
AMB-1 extract	Li et al. (2012)	0.22	0.23	–
MV-1 extract	Wang et al. (2013)	0.31	–	0.092
Δ*mamJ* mutant	Katzmann et al. (2013)	0.23	–	0.36
Δ*mamJ*Δ*lim* ΔMIS mutant	This study	0.31	–	0.53
Extracellular magnetite				
*Geobacter metallireducens*	Moskowitz et al. (1993)	0.03	0.25	–
*Shewanella putrefaciens*	Carvallo et al. (2008)	0.14	–	–
*Acidovorax on green rust*	Miot et al. (2014)	–	–	0

a Citrate-Bicarbonate-Dithionite (CBD) extractable component calculated from the difference between pre-CBD and post-CBD measurements.

**Table 2.** Room-Temperature Boltzmann Factors β_0_ Associated With the Denucleation of High-Moment States (HMS) and Low- Moment States (LMS) for Various Chain Geometries With N Equidimensional Magnetosomes of 50 nm Diameter, Separated by a Diameter-Normalized Gap g

Chain geometrys	Gap *g*	*N*	*β*_0_ (HMS)	*β*_0_ (LMS)

Single-stranded	0.1	2	439.9	–
	0.1	7	763.9	–
	0.1	8	784.2	–
	0.1	9	795.0	–
	0.1	15	835.9	–
	0.1	16	835.7	–
	0.1	17	842.9	–
	0.05	17	969.2	–
Double-stranded (native)	0.1	10 + 9	667.8	800.4
	0.1	10 + 10	688.5	803.1
	0.1	11 + 10	705.2	801.6
	0.1	11 + 11	751.5	829.2
	0.05	11 + 11	864.0	953.4
Double-stranded (fold-collapsed)	0.1	10 + 9	235.0	1,119
	0.1	10 + 10	268.4	1,345
	0.1	11 + 10	277.7	1,210
	0.1	11 + 11	242.2	1,367
	0.05	11 + 11	278.5	1,572
Ring	0.1	12	65.2	1,518
	0.1	16	55.5	1,606
	0.1	20	27.0	1,633
	0.05	20	31.0	1,877

**Table 3.** Central Ridge Magnetization M_cr_ and Total FORC Magnetization M_forc_ From Micromagnetic Simulations of (a) Single-Stranded and Double-Stranded Chains, (b) a Composite of Intact Chain configurations With a 2:1 Proportion of Equant Versus Prismatic Magnetosomes, (c) a Composite of All Chain configurations With 2:1 Proportion of Equant Versus Prismatic Magnetosomes (Figure 8b, and (d) the CBD-Extractable Fraction of a Magnetofossil-Rich Pelagic Carbonate From the Equatorial Pacific (Ludwig et al.,2013)

SD system	*M*_cr_/*M*_s_	*M*_forc_/*M*_s_	*M*_cr_/*M*_rs_	*M*_cr_/*M*_forc_

Stoner-Wohlfarth	0.272	0.272	0.544	1.0
Single-stranded equidimensional	0.347	0.379	0.704	0.914
Single-stranded prismatic	0.355	0.374	0.711	0.947
Double-stranded equidimensional	0.194	0.396	0.407	0.489
Double-stranded prismatic	0.209	0.367	0.424	0.570
Fold-collapsed equidimensional	0.171	0.397	0.366	0.431
Fold-collapsed prismatic	0.187	0.354	0.381	0.528
Composite chains (intact)	0.274	0.382	0.561	0.718
Composite chains (all)	0.242	0.382	0.500	0.632
Pelagic carbonate (CBD-extractable)	–	–	0.653	0.666

**Table 4:** RPI Parameters for Different Types of SD Remanence Carriers Made of n SD Crystals With Mean Magnetic Moment m_SD_

SD configuration	NRM *m*_0_/*m*_SD_	IRM *m*_IRM_/*m*_SD_	ARM *m*_ARM_/*r*_a_*m*_SD_	*R (IRM)* $m_{0}^{2}/m_{\text{IRM}}$	*R (ARM)* $r_{\text{a}}m_{0}^{2}/m_{\text{ARM}}$

Silicate inclusions	$\sim 0.9\sqrt{n}$	~0.5*n*	$\sim 0.9\sqrt{n}$	~1.7	$\sim 0.9\sqrt{n}$
Single-stranded chains	*n*	0.5*n*	*n*	2*n*	*n*
Double-stranded chains	*n*	0.5*n*	*δn*	2*n*	*n*^2^/*δ*
Fold-collapsed chains	*δn*	0.5*n*	*δn*	2*δ*^2^/*n*	*δn*

*Note.* Other symbols: *m*_0_—native magnetic moment, *m*_IRM_—magnetic moment after IRM acquisition, *m*_ARM_—magnetic moment after ARM acquisition, *r*_a_—ARM/IRM of isolated SD crystals, δ*n*—number of magnetosomes with uncompensated magnetic moment in the FC configuration of double–stranded and fold–collapsed chains.

## Data Availability

All data generated in this work and the Mathematica scripts used for micromagnetic simulations are available on https://figshare.com/collections/Magnetic_Signatures_of_Magnetofossils/5579547.

## Key Points:

- Fossilized magnetotactic bacteria in sediments bear magnetic signatures associable with different sources of magnetite particles
- Single-stranded, multi-stranded, and fold-collapsed magnetosome chains explain all observed signatures of fossilized magnetotactic bacteria
- We provide criteria for the discrimination of distinct populations of ancient magnetotactic bacteria
