# Supplementary material for: Key Signatures of Magnetofossils Elucidated by Mutant Magnetotactic Bacteria and Micromagnetic Calculations
Source: J Geophys Res Solid Earth. 2022 Jan 18;127(1):e2021JB023239. doi: 10.1029/2021JB023239 (PMC9017866; doi:10.1029/2021JB023239)
Supplement: Supplementary file 1 — Supporting Information S1 [file JGRB-127-0-s004.pdf]

AGU PUBLICATIONS

Journal of Geophysical Research: Solid Earth

Supporting information for

Key signatures of magnetofossils elucidated by mutant magnetotactic bacteria and micromagnetic calculations

Matthieu Amor [1,2], Juan Wan [1], Ramon Egli [3,4†], Julie Carlut [4], Christophe Gatel [5,6], Ingrid Marie Anderssen [5], Etienne Snoeck [5], Arash Komeili [1,7]

[1] Department of Plant and Microbial Biology, University of California, Berkeley, CA 94720-3102

[2] Aix-Marseille-University, CEA, CNRS, BIAM, 13108 Saint-Paul-lez-Durance, F-13108 France

[3] Zentralanstalt für Meteorologie und Geodynamik (ZAMG), Hohe Warte 38, A-1190 Vienna, Austria

[4] Université de Paris, Institut de Physique du Globe de Paris, CNRS, 75005 Paris, France

[5] CEMES CNRS, 29 Rue Jeanne Marvig, F-31055 Toulouse, France

[6] Université de Toulouse, F-31077 Toulouse, France

[7] Department of Molecular and Cell Biology, University of California, Berkeley, CA 94720-3200

Contents of this file

1. Supporting Table S1
2. Supporting Figures S1-S8
3. Glossary of magnetic terms
4. Supporting information: AMB-1 mutant
5. Supporting information: Micromagnetic calculations

† Corresponding Author: ramon.egli@zamg.ac.at

Supporting Tables

Table S1: Parameter values used for chain simulation. Statistical distributions ($\mathcal{F}$ = Fischer-Von Mises, $\mathcal{B}$ = Beta, $\mathcal{N}$ = Normal, $\mathcal{U}$ = Uniform) indicate that the corresponding parameter is a random realization of the given distribution, e.g. $10\mathcal{B}(4,4)$ means 10 times a random variate with Beta distribution $\mathcal{B}(4,4)$. See supporting information for the parameter definition.

**Parameter**	**Single-stranded chains**	**Native double-stranded chains**	**Fold-collapsed chains**
Axes orientation			
$\measuredangle(\mathbf{a}_3,\hat{\mathbf{z}})$	$\mathcal{F}(\varkappa_s)$	$\mathcal{F}(\varkappa_s)$	$\mathcal{F}(\varkappa_s)$
$\measuredangle(\mathbf{c}_3,\hat{\mathbf{z}})$	$\mathcal{F}(\varkappa_s)$	$\mathcal{F}(\varkappa_s)$	$\mathcal{F}(\varkappa_s)$
$\varkappa_s^{-1}$, deg	$10\mathcal{B}(4,4)$	$10\mathcal{B}(4,4)$	$10\mathcal{B}(4,4)$
Size			
s_0 (equant, nm)	$150\mathcal{B}(4,6)$	$150\mathcal{B}(4,6)$	$150\mathcal{B}(4,6)$
s_0 (prisms, nm)	$130\mathcal{B}(5,7.3)$	$130\mathcal{B}(5,7.3)$	$130\mathcal{B}(5,7.3)$
$\delta s/s_0$	$\mathcal{N}(0,\sigma_s)\in[-0.8,+0.8]$	$\mathcal{N}(0,\sigma_s)\in[-0.8,+0.8]$	$\mathcal{N}(0,\sigma_s)\in[-0.8,+0.8]$
σ_s	$0.1\mathcal{B}(4,4)$	$0.1\mathcal{B}(4,4)$	$0.1\mathcal{B}(4,4)$
Elongation			
λ_0 (equant)	1	1	1
λ_0^{-1} (prisms)	$0.694+\mathcal{B}(10,4)$	$0.694+\mathcal{B}(10,4)$	$0.694+\mathcal{B}(10,4)$
Chamfering			
w_0	$\mathcal{U}(0.02,0.15)$	$\mathcal{U}(0.02,0.15)$	$\mathcal{U}(0.02,0.15)$
$\delta w/w_0$	$\mathcal{N}(0,\sigma_s)\in[-0.8,+0.8]$	$\mathcal{N}(0,\sigma_s)\in[-0.8,+0.8]$	$\mathcal{N}(0,\sigma_s)\in[-0.8,+0.8]$
Chain length/lag			
N_1	$\mathcal{U}(4,9)$	$\mathcal{U}(5,7)$	$\mathcal{U}(5,7)$
N_2	—	$\mathcal{U}(N_1-2,7)$	$\mathcal{U}(N_1-2,7)$
l	—	$\mathcal{U}(0,2)$	0
Gaps			
g_0 (equant, nm)	$\mathcal{U}(1,5)$	$\mathcal{U}(1,5)$	$\mathcal{U}(1,5)$
g_0 (prisms, nm)	$\mathcal{U}(2,10)$	$\mathcal{U}(2,10)$	$\mathcal{U}(2,10)$
$\delta g/g_0$	$\mathcal{N}(0,\sigma_z)$	$\mathcal{N}(0,\sigma_z)$	$\mathcal{N}(0,\sigma_z)$
δg	$\in[g_{\min},2g_0]$	$\in[g_{\min},2g_0]$	$\in[g_{\min},2g_0]$
$g_{\min}$ (equant)	0.5 nm	0.5 nm	0.5 nm
Transverse position			
$\delta x/s_0$, $\delta y/s_0$	$\mathcal{N}(0,\sigma_x)$	$\mathcal{N}(0,\sigma_x)$	$\mathcal{N}(0,\sigma_x)$
σ_x	$0.05\mathcal{B}(4,4)$	$0.05\mathcal{B}(4,4)$	$0.05\mathcal{B}(4,4)$
Chain tapering			
s_t	$\mathcal{U}(0.5,1)$	$\mathcal{U}(0.5,1)$	$\mathcal{U}(0.5,1)$
n_t	$\mathcal{U}(1,N/2)$	$\mathcal{U}(1,N/2)$	$\mathcal{U}(1,N/2)$
Chain deformation			
β_b (deg)	$12\mathcal{B}(4,4)$	$12\mathcal{B}(4,4)$	$12\mathcal{B}(4,4)$
β_t (deg)	—	$30\mathcal{B}(4,4)$	$30\mathcal{B}(4,4)$

Supporting Figures

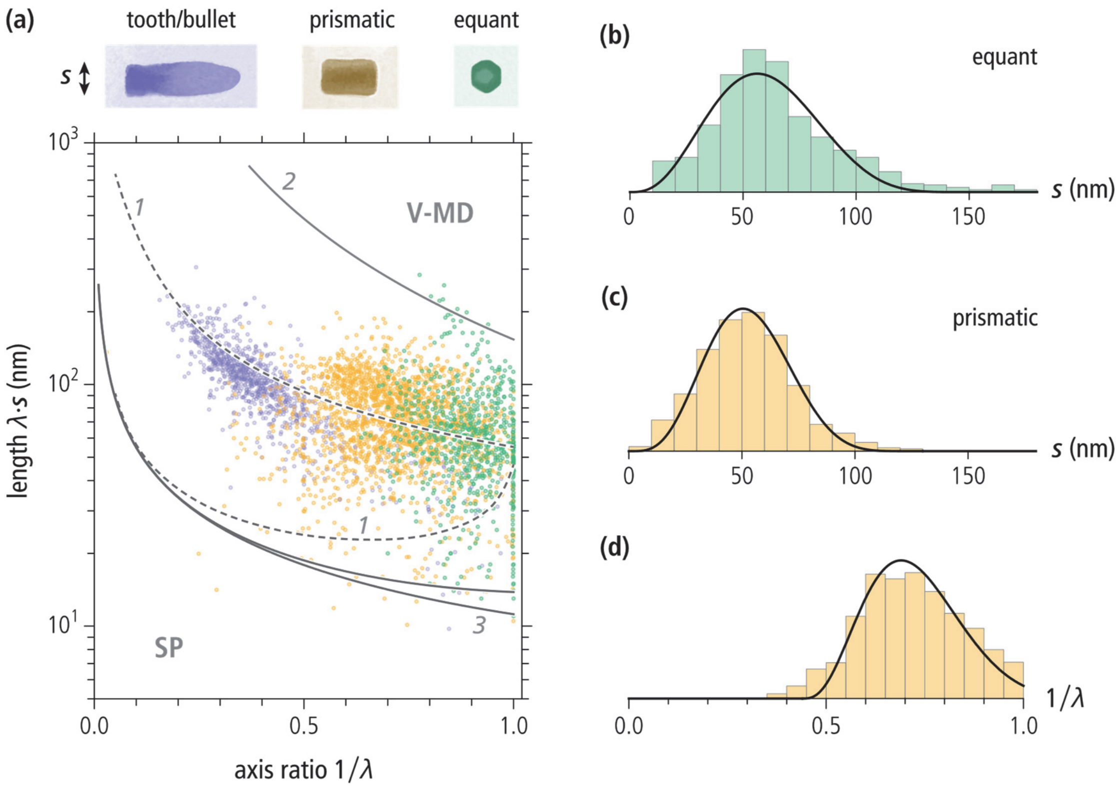

Figure S1: Magnetofossil shapes and sizes used for micromagnetic modeling. **(a)** Inverse elongation $1/\lambda$ vs. crystal length for 3441 magnetofossil crystals classified as equant (cuboctahedral, octahedral), prismatic, or tooth/bullet-shaped, obtained from TEM micrographs of magnetotactic bacteria and magnetic extracts. Three representative examples from Isambert et al., (2007) are shown above: s is the crystal width. Dashed lines "*1*" represent the magnetic stability limits of isolated, single-domain magnetite crystals after Butler and Banerjee (1975). The solid line "*2*" is the upper stability limit for magnetosome chains with gaps $g/s = 0.1$ between crystals (Muxworthy and Williams, 2006). Above this limit, magnetic vortices (V) or multiple magnetic domains (MD) nucleate spontaneously. The pair of solid lines "*3*" is the lower stability limit for magnetosome chains with gaps $g/s = 0.6$ (upper curve) and $g/s = 0$ (lower curve) at room temperature (Newell, 2009). Below this limit, magnetosome chains are superparamagnetic (SP). **(b)** Histogram of short axis length for all equant crystals in (a), compared with the rescaled Beta distribution $\mathcal{B}(s/\overline{s};\beta_1,\beta_2)$ with $\overline{s} = 150$ nm, $\beta_1 = 4$, $\beta_2 = 6$ (black line). **(c)** Histogram of short axis length for all prismatic crystals in (a), compared with the rescaled Beta distribution $\mathcal{B}(s/\overline{s};\beta_1,\beta_2)$ with $\overline{s} = 130$ nm, $\beta_1 = 5$, $\beta_2 = 7.3$ (black line). **(d)** Histogram of inverse elongation for all prismatic crystals in (a), compared with the rescaled Beta distribution $\mathcal{B}(1/\overline{\lambda} - 1/\lambda;\beta_1,\beta_2)$ with $1/\overline{\lambda} = 0.694$, $\beta_1 = 10$, $\beta_2 = 4$ (black line). Data from Isambert et al. (2007), Petersen et al. (1986, 1989), Mann et al. (1987), Vali et al. (1987), McNeill (1990), Sparks et al. (1990), Meldrum et al. (1993b), Hesse (1994), Iida and Akai (1996), Lean and McCave (1998), Simpson et al. (2005), Lippert and Zachos (2007), Moisescu et al. (2008), and Lefèvre et al. (2011).

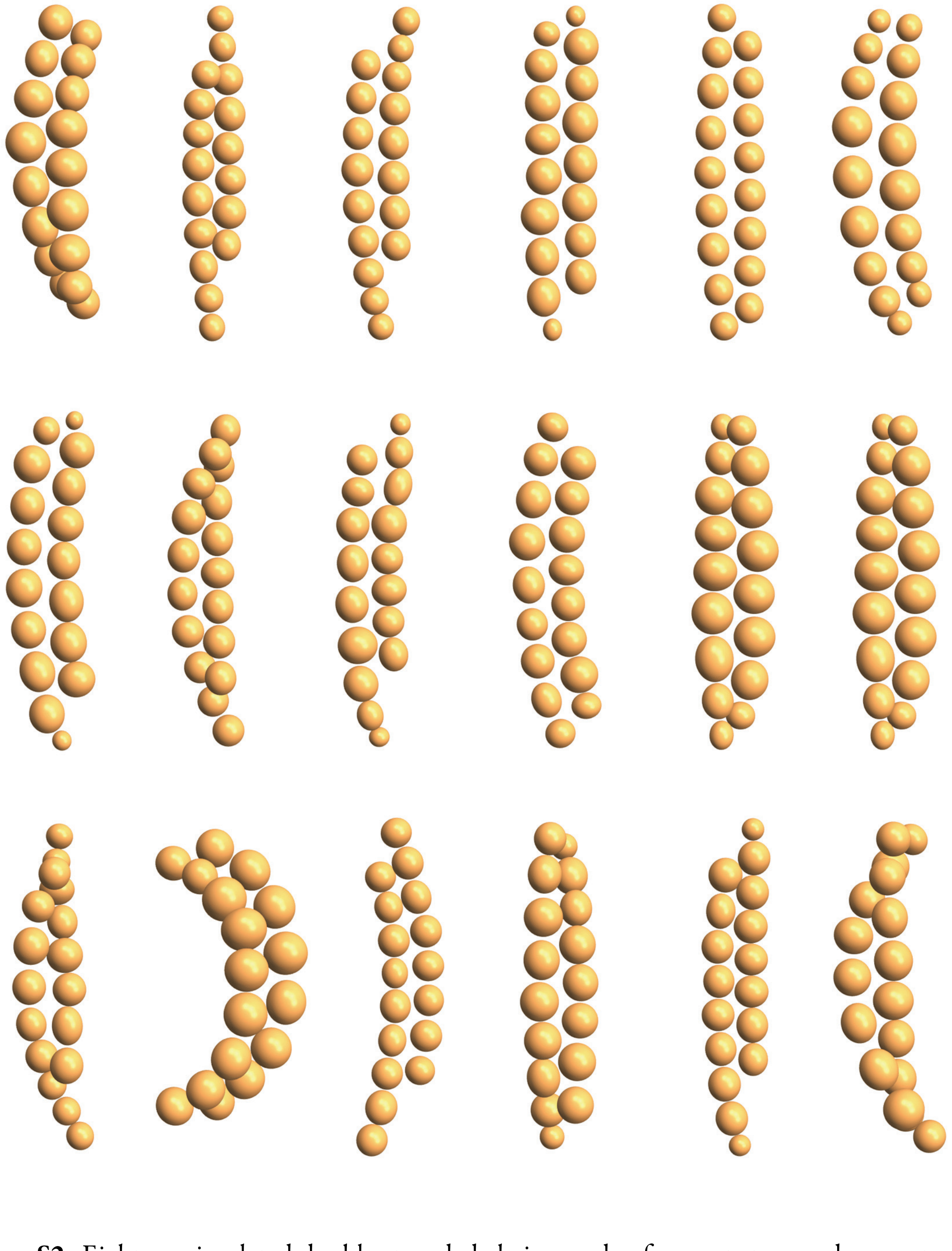

Figure S2: Eighteen simulated double-stranded chains made of equant staggered magnetosomes with $N_1 = 9$, $N_2 = 8$, and other parameters chosen according to Table S1.

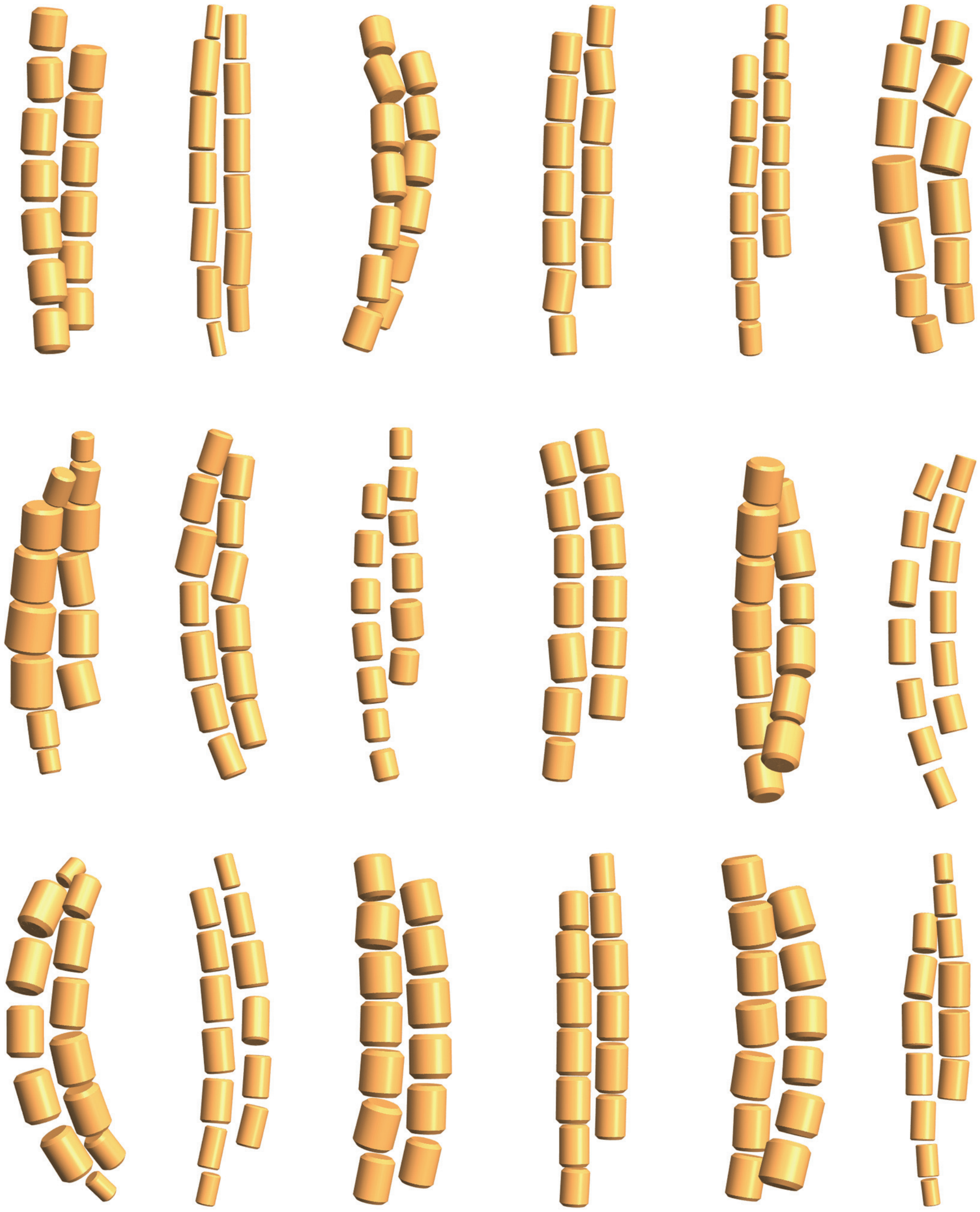

Figure S3: Eighteen simulated double-stranded chains made of prismatic staggered magnetosomes with $N_1 = 7$, $N_2 = 6$, and other parameters chosen according to Table S1.

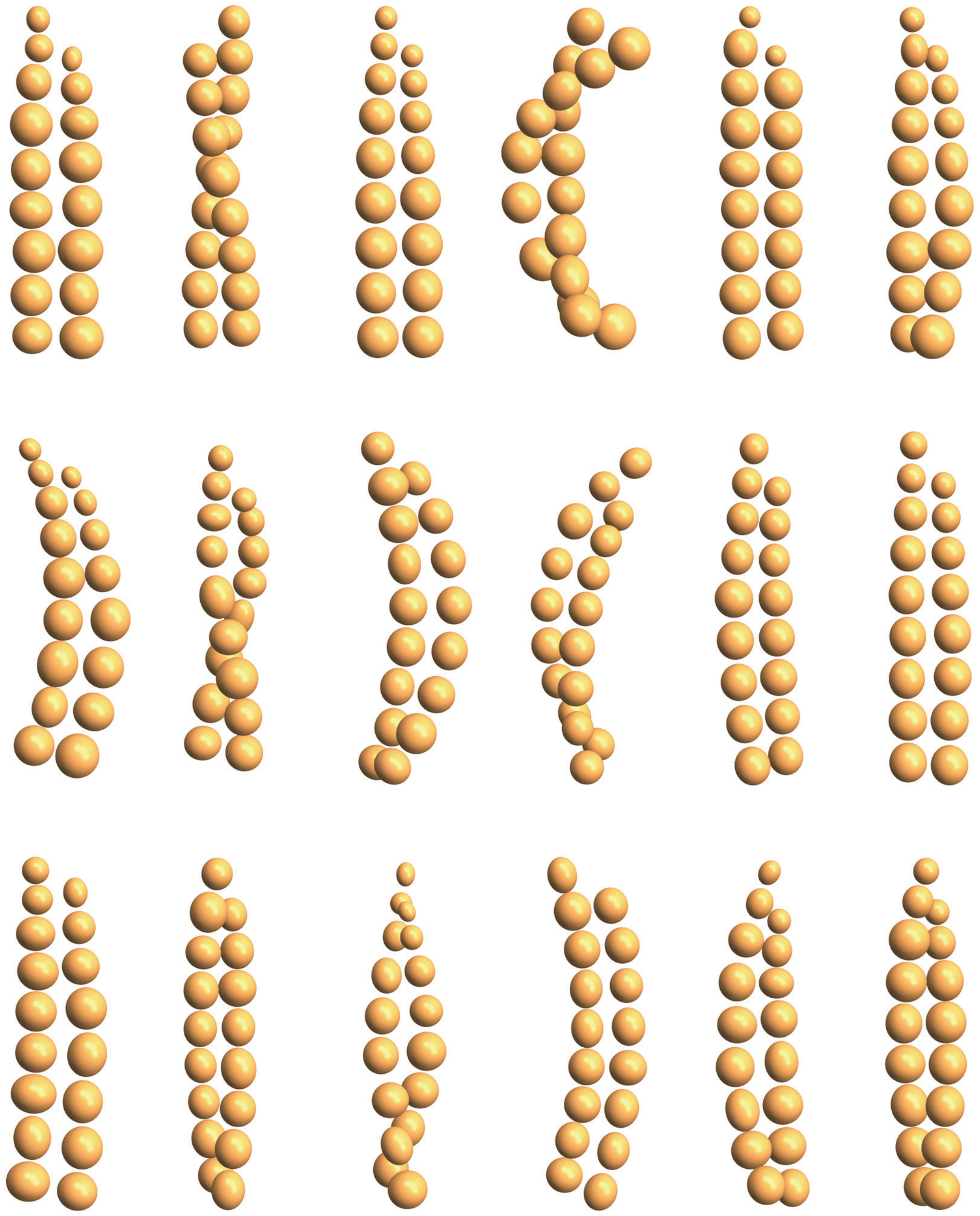

Figure S4: Eighteen simulated double-stranded chains resulting from complete folding of a single-stranded chain made of equant magnetosomes with $N_1 = 9$, $N_2 = 8$, and other parameters chosen according to Table S1.

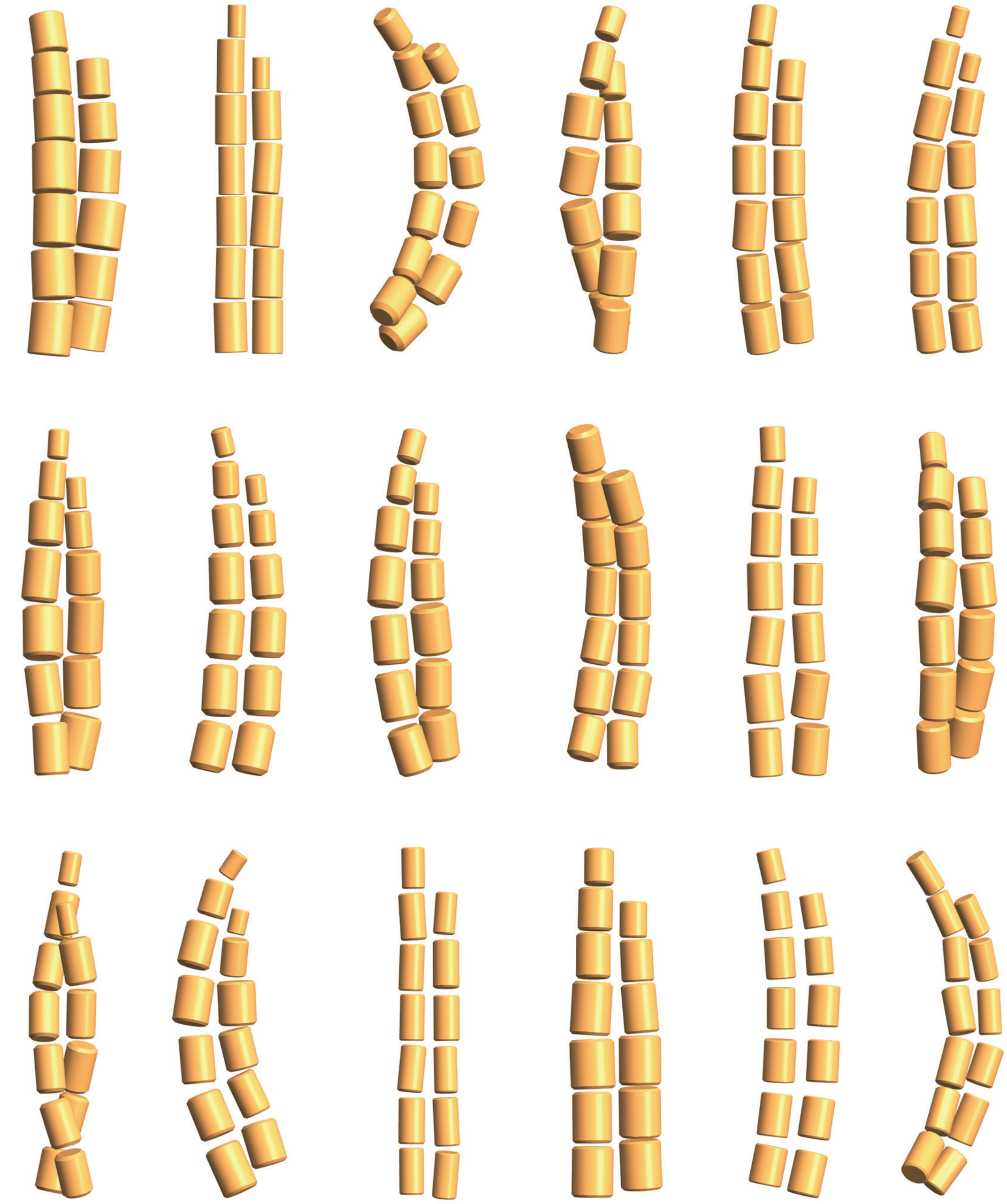

Figure S5: Eighteen simulated collapsed double chains resulting from complete folding of a single-stranded chain made of prismatic magnetosomes with $N_1 = 7$, $N_2 = 6$, and other parameters chosen according to Table S1.

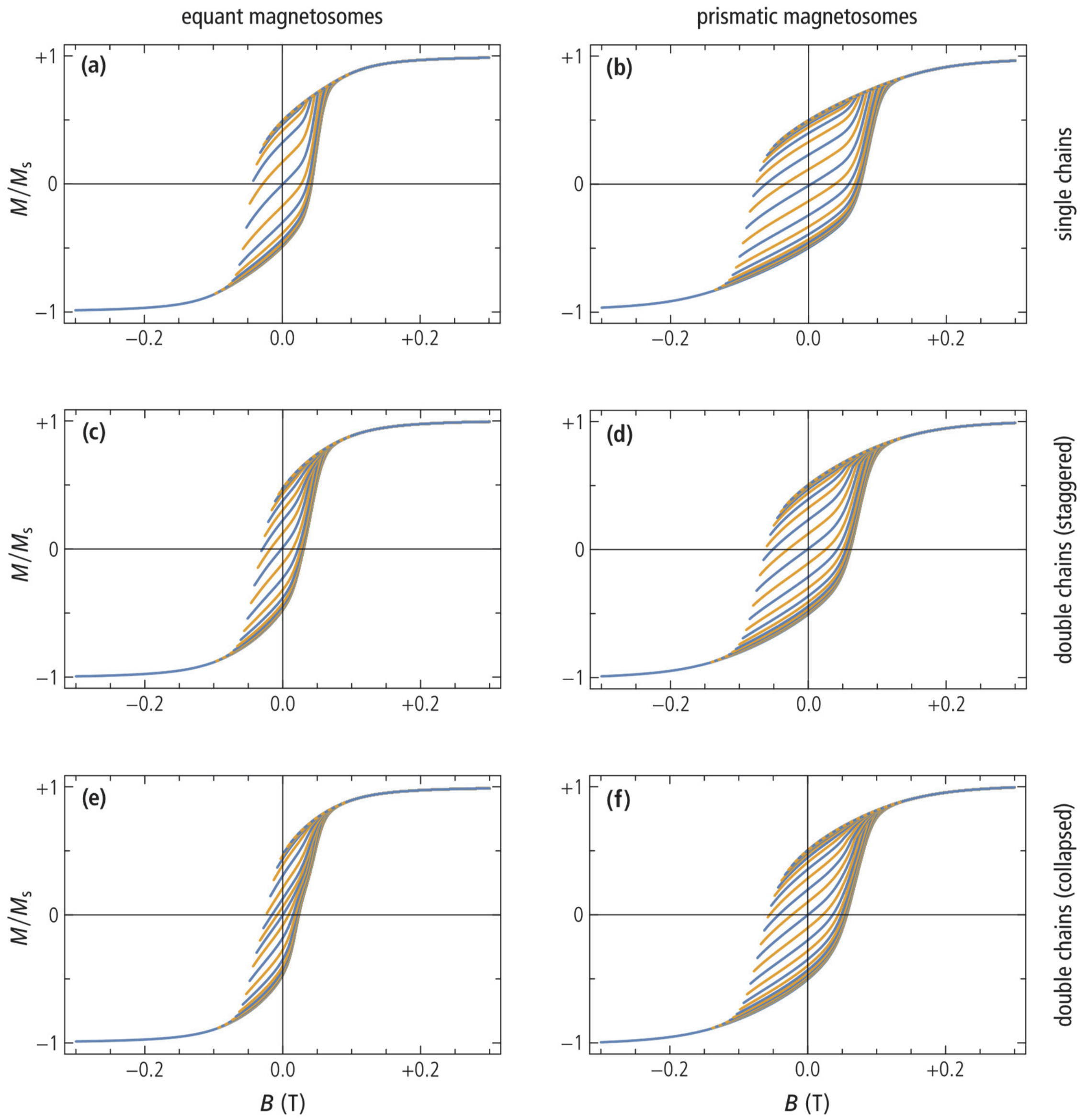

Figure S6: Simulated FORC measurements obtained from micromagnetic simulations of six combinations of magnetosome shape (equant or prismatic), and chain geometry (single-stranded, native double-stranded, and double-stranded from complete folding of a single-stranded chain).

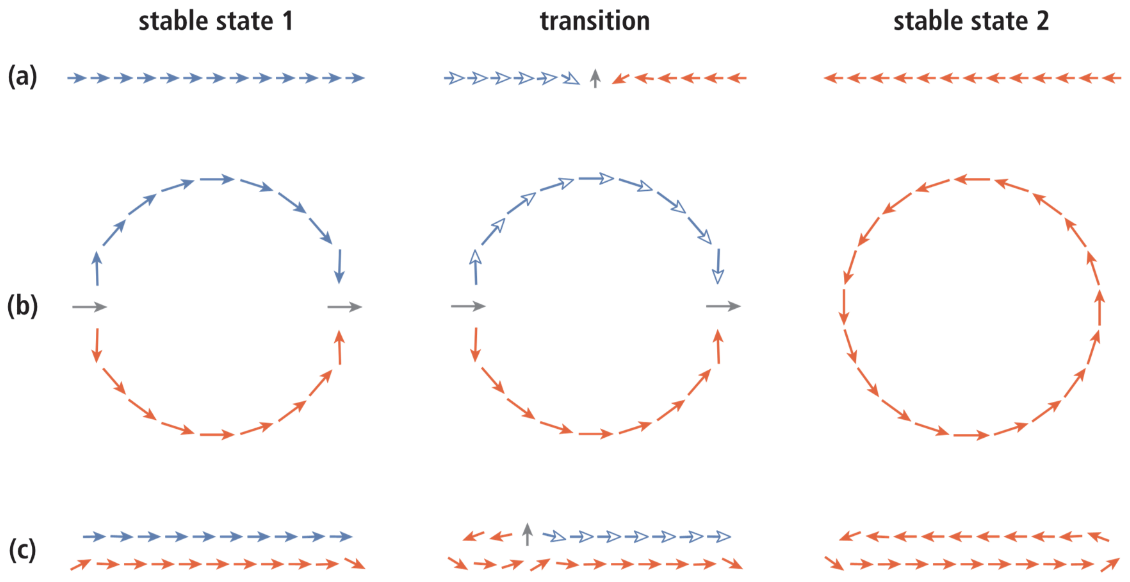

Figure S7: Calculation of the transition between stable states in a single-stranded chain (**a**), a ring (**b**), and a double-stranded chain (**c**). The magnetic moments if individual particles are indicated by arrows. Blue and red arrows indicate magnetic moments belonging to two different domains, gray arrows the magnetic moments with intermediate directions that separate the domains. Open arrowheads in the transitional configuration used to calculate the transition path (middle column) indicate magnetic moments in the domain that is denucleated to complete the transition. Domain denucleation is promoted by a slight weakening of the dipolar interactions between the magnetic moments with open arrowheads.

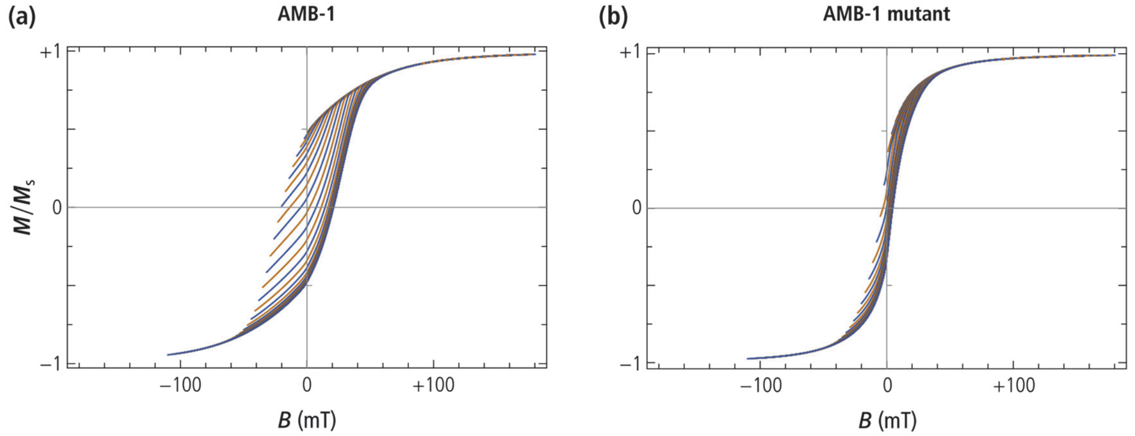

Figure S8: High-resolution FORC measurements of wild-type AMB-1 (**a**) and the AMB-1 mutant (**b**), normalized by the saturation magnetization M_s. Every 10th of the 636 curves is shown for clarity. Notice the slight loop constriction of the AMB-mutant at $M = 0$.

Supporting Movies

Movie S1: Simulated zero-field reversal of a single-stranded chain. The chain is made of 15 SD particles without shape and magnetocrystalline anisotropy.

Movie S2: Simulated zero-field transition between the high- and low-moment states of a ring. The ring is made of 20 SD particles without shape and magnetocrystalline anisotropy.

Movie S3: Simulated zero-field transition between the high- and low-moment states of a double-stranded chain. The strands contain 11 and 10 SD particles, respectively, without shape and magnetocrystalline anisotropy.

Movie S4: Simulated zero-field transition between the high- and low-moment states of a fold-collapsed chain. The two strands contain 11 and 10 SD particles, respectively, without shape and magnetocrystalline anisotropy.

Glossary of magnetic terms

Anhysteretic remanent magnetization (ARM): Remanent magnetization obtained by placing the specimen in a decaying alternating magnetic field superimposed to a small direct current (DC) field. ARM is strongly selective towards non-interacting SD particles and magnetic systems that behave equivalently, such as chains. This selectivity is similar, but not identical, to that of the central ridge in FORC diagrams. The anhysteretic susceptibility, $\chi_a = \mathrm{ARM}/H_{dc}$ is defined as the ARM normalized by the DC field H_{dc} (in A/m) used during acquisition.

Applied field (B): In the FORC measurement protocol, it is the magnetic field applied during magnetic measurements.

ARM ratio (χ_a/IRM): The ARM ratio χ_a/IRM, where IRM is the isothermal remanent magnetization acquired in a DC field equal to the maximum amplitude of the AC field used to obtain the ARM, is a magnetic grain size indicator. Non-interacting SD particles and intact magnetosome chains are characterized by $\chi_a > 1\,\mathrm{mm/A}$.

Bias field (B_u): Vertical coordinate of the FORC diagram. It has a direct physical interpretation only in the Preisach-Néel model of interacting SD particles, where it corresponds to the internal interaction field acting on individual particles.

Biogenic soft (BS): Typically used to describe the magnetic signature of conventional (i.e., not giant) magnetofossils made of equidimensional crystals. The coercivity distribution is characterized by a median field of ~45 mT and a mean dispersion parameter of ~0.18. The magnetic anisotropy required to explain the range of coercivities covered by this component (~10-100 mT) originates largely from the chain structure, since individual crystals are almost isotropic.

Biogenic hard (BH): Typically used to describe the magnetic signature of conventional (i.e., not giant) magnetofossils made of elongated crystals. The coercivity distribution is characterized by a median field of ~70 mT and a mean dispersion parameter of ~0.1. The magnetic anisotropy required to explain the range of coercivities covered by this component (~30-140 mT) originates from the chain structure and from magnetosome elongation.

Blocking volume: The minimum volume that a SD magnetic particle must have to maintain a stable magnetization at a given temperature.

Boltzmann factor: The ratio $\beta = \Delta E/k_B T$ between the energy barrier ΔE that needs to be overcome for a transition between two given magnetic states at the absolute temperature T, and the energy $k_B T$ of thermal fluctuations, where k_B is the Boltzmann constant. The frequency of transitions occurring under these conditions is $\nu = \tau_0^{-1} e^{-\beta}$, where $\tau_0 \approx 0.1 \ldots 1\ \mathrm{ns}$ is the time constant of thermal fluctuations.

Central ridge: Name given to a distinct feature of FORC diagrams, which consists of a narrow ridge extending along, or very close to the horizontal axis defined by $B_u = 0$. It is a distinctive feature of isolated magnetic systems possessing only few magnetic states. Notable examples are non-interacting SD and vortex particles, and systems of particles that mimic these domain states, such as magnetosome chains. Because of its quasi-unidimensional nature, the central ridge is separable from other contributions to the FORC diagram. In sediments, the central ridge is often uniquely associated with secondary SD magnetic particles and magnetofossils, since primary production of non-interacting SD magnetite requires very fast cooling and magnetic particles with larger sizes are rarely limited to the narrow range of vortex particles.

Coercivity (B_c): In the context of magnetic hysteresis, it is defined as the absolute value of the applied field for which the magnetization is zero during the measurements of a major hysteresis loop; that is, $M_-(B_c) = 0$ for the ascending branch M_-, and $M_+(-B_c) = 0$ for the descending branch M_+. It is a common measure of the "magnetic hardness" of a specimen. In case of magnetic particle assemblages, B_c depends strongly on the domain state and on magnetic interactions. In SD particles, B_c is a measure of their magnetic anisotropy energy, with larger values in case of strong particle elongation or strong magnetocrystalline anisotropy. In MD particles, B_c depends essentially on how strongly pinned are domain walls by crystal defects. B_c is also used to denote the horizontal coordinate of FORC diagrams, where it is interpreted as the coercivity of rectangular hysterons associated with the FORC function.

Coercivity of remanence (B_{cr}): It is defined as the DC field that must be applied in the opposed direction to cancel the saturation remanent magnetization M_{rs}, obtaining a zero remanent magnetization. In FORC measurements, it coincides with the reversal field B_r of the curve that yields $M(B_r, B) = 0$ in $B = 0$. It is a measure of the "magnetic hardness" of the remanent magnetization of a specimen, having thus a similar meaning as B_c. The so-called coercivity ratio B_{cr}/B_c is always ≥ 1 if FORC curves are limited by the major hysteresis loop, as usually observed in geologic materials. The coercivity ratio of particle assemblages depends on the domain state of particles, with values close to 1 for SD assemblages and values $\gg 1$ for MD particles.

Coercivity or switching field distribution: The strict definition refers to the statistical distribution $f(B_{sw})$ of switching fields in systems possessing large numbers of magnetic states (e.g., those of individual particles. Because switching fields are rarely directly measurable, a coercivity or switching field distribution is empirically identified with any field-dependent function $f(B)$, whose integral coincides with a certain type of total magnetization (for instance the saturation remanent magnetization), or total magnetization of a subset of particles in a specimen. Vice versa, coercivity distributions can be defined as the first derivative of a so-called magnetization curve $M(B)$ obtained with a specific measurement protocol. In these cases, the argument of the function, B, is a generic field that is not strictly identifiable with the coercive field of hysteresis, nor with a switching field. Coercivity distributions commonly used in the literature include those associated with IRM acquisition, DC demagnetization, AF demagnetization of IRM or ARM, and the central ridge.

Coercivity distribution (AF demagnetization, f_{af}): The derivative $-\partial M/\partial B_{af}$ of the AF demagnetization curve of a remanent magnetization (ARM or IRM), obtained in successive demagnetization steps with maximum AF amplitude B_{af}. The integral of f_{af} yields the initial remanent magnetization. Therefore, f_{af} can be identified with the coercivity distribution of magnetic minerals carrying the specific type of remanent magnetization being demagnetized.

Coercivity distribution (DC demagnetization, f_{dcd}): The derivative $½\,\partial M/\partial B_r$ of the DCD demagnetization curve $M(B_r)$ with respect to the DC demagnetization field B_r. The integral of f_{dcd} yields the saturation remanent magnetization M_{rs}. Therefore, f_{dcd} can be identified with the coercivity distribution of magnetic minerals carrying a saturation remanent magnetization. It is obtained from FORC measurements by $f_{dcd}(x) = -½\partial M(-x, B=0)/\partial x$.

Coercivity distribution (IRM acquisition, f_{irm}): The derivative $\partial M/\partial B$ of the IRM acquisition curve $M(B)$ with respect to the IRM acquisition field B_r. The integral of f_{irm} yields the saturation remanent magnetization M_{rs}. Therefore, f_{irm} can be identified with the coercivity distribution of magnetic minerals carrying a saturation remanent magnetization. It is similar, but not identical, to the coercivity distribution obtained from DC demagnetization, the difference being that IRM acquisition starts from a fully demagnetized state.

Coercivity distribution (irreversible hysteresis, f_{irr}): The derivative $½\,\partial M_{irr}/\partial B$ of the irreversible component M_{irr} of the ascending branch of the major hysteresis loop with respect to the applied field B. The integral of f_{irr} is $\le M_s$, where M_s is the saturation magnetization. Unlike other coercivity distributions, f_{irr} is defined for positive and negative arguments, as the major hysteresis loop exists for positive and negative fields, whereby $f_{irr}(B<0)$ represents the irreversible contributions from magnetic states that do not exist in a null field. Non-interacting SD particles are characterized by $f_{irr}(B<0)=0$. This coercivity is obtained from FORC measurements by $f_{dcd}(x) = -½\partial M(B_r, B)/\partial B_r$ with $B_r = B = x$.

Coercivity distribution (central ridge, f_{cr}): It is obtained by integrating the central ridge contribution $\rho_{cr}(B_c, B_u)$ to the FORC function over B_u. The integral of f_{cr} over B_c yields the total contribution of irreversible magnetization processes to the central ridge. This is a fraction of all irreversible contributions to the hysteresis loop, so that $f_{cr} \le f_{irr}$ for any positive field, with $f_{cr} = f_{irr}$ for non-interacting uniaxial SD particles.

Direct current demagnetization (DCD): The procedure used to null the saturation remanence M_{rs} of a specimen, consisting in the application and removal of a field B_{cr}, called coercivity of remanence, in the opposed direction to M_{rs}. Because B_{cr} is not known a priori, increasingly large 'reversal' fields B_r are applied and removed, and the resulting remanent magnetization $M_r(B_r)$ is measured after each step. In doing so, M_r goes from $+M_{rs}$ for $B_r = 0$, to $-M_{rs}$ when B_r has reached the saturation field, passing through $M_r = 0$ for $B_r = B_{cr}$. The resulting remanent magnetization curve is called DC demagnetization curve. It is formally equivalent to the subset $M(B_r, B=0)$ of FORC measurements.

Domain state: Describes the magnetization geometry inside a ferrimagnetic crystal, depending on whether it contains a single homogeneously magnetized domain (single domain, SD) or several such domains (multidomain, MD). Materials with small magnetocrystalline anisotropy feature intermediate states consisting of magnetic vortices or other magnetization patterns that tend to cancel the stray field (flux closure). Magnetic properties are strongly influenced by the domain state, which in turn depends on the size, shape, and arrangement of particles, as well as time and temperature.

Energy barrier (ΔE): The minimum increase in the free energy E of a magnetic system required for a transition between two magnetic states while external conditions (e.g., temperature, applied field) are kept constant. If ΔE is sufficiently small, typically ~20 times the energy $k_B T$ of thermal fluctuations (see Boltzmann factor), the transition occurs spontaneously within a certain time.

Ferrimagnetism: Describes a class of magnetic materials that includes ferromagnetism (parallel atomic spins), and strict ferrimagnetism (two antiparallel spin lattices with unequal magnetic moments). The common characteristics of these materials is that they possess a spontaneous magnetization, defined as the magnetization of any microscopic (sub-domain) volume of the material. The saturation magnetization of ferrimagnetic particles is equal to the spontaneous magnetization of the material of which they are made, up to fields that are sufficiently large to overcome the given ferrimagnetic order.

First-order reversal curves (FORCs): Series of partial hysteresis curves directly originating (order 1) from the major hysteresis (order 0). In the most commonly used measurement protocol, a FORC curve is measured as follows: (1) a positive saturation field B_s is applied, which resets the specimen to a positive saturation state, (2) the field is decreased until a predefined, so-called reversal field $B_r < B_s$ is reached, (3) the field is paused for a predefined time at B_r (typically for ~1-2 s), (4) measurements are taken at regular field intervals while the applied field is increased from B_r to positive saturation. The set of curves $M(B_r, B)$ obtained for a sequence of B_r values established by the protocol is a scan of the space enclosed by the major hysteresis loop. It defines the FORC function $\rho = -½\,\partial^2 M/\partial B_r \partial B$. Each value of ρ in the transformed coordinates $B_c = (B - B_r)/2$ and $B_u = (B + B_r)/2$ represents the contribution of a fictive rectangular hysteresis loop (called hysteron) with coercive field B_c and subjected to a fixed bias field B_u. In the case of ensembles of interacting SD particle assemblages, B_c and B_u are identifiable with the coercivity and the interaction field of each particle. The central ridge is the limit case with no interactions, in which case ρ is condensed along $B_u = 0$. In other systems, ρ reflects reversible and irreversible magnetization processes responsible for the differences between consecutive FORCs, being no longer associable with the coercivity and interaction field distribution of discrete particles.

Fluctuation field (B_f): A fictive field defined as the difference between the fields required to trigger a given transition between magnetic states without and with the effect of thermal fluctuations (random excursions of the magnetization caused by thermal agitation). These fluctuations are then equivalent to a fluctuating external field with amplitude B_f. Magnetic particles become effectively superparamagnetic when B_f is as large as the field required for the transition without fluctuations.

Flux closure (FC): Magnetic configuration of a single particle, a group of particles, or any other ferrimagnetic system, which produces a closed internal magnetic flux $B_i = H + M$. FC states tend to minimize or nullify the net magnetic moment of the system. The vortex state (VO) of single particles is an example of flux closure configuration. Magnetic domains in soft materials also tend to form flux closure configurations in sufficiently small external fields.

FORC diagram: The graphical representation of the FORC function $\rho = -½\,\partial^2 M/\partial B_r \partial B$ obtained from FORC measurements. It is usually represented using the transformed coordinates $B_c = (B - B_r)/2$ (coercive field) and $B_u = (B + B_r)/2$ (bias field) on the horizontal and vertical axes, respectively.

Hysteresis loop: The in-field magnetization $M(B)$ of a specimen measured while decreasing the field from a maximum value B_{max} to a minimum value $B_{min} < B_{max}$, which gives the descending branch $M_+(B)$ of the loop, and then increasing the field from B_{min} to B_{max}, which gives the ascending branch $M_-(B)$. All non-SP ferrimagnetic materials are characterized by $M_+ \neq M_-$ if $-B_s < B_{min} < B_{max} < +B_s$, where B_s is the saturation field. The hysteresis loop is called major if $B_{min} \leq -B_s$ and $B_{max} \geq +B_s$.

Hysteresis squareness or remanence ratio (M_{rs}/M_s): Describes the vertical opening of the major hysteresis loop and is defined as the ratio between saturation remanent magnetization and saturation magnetization. It is called squareness because hysteresis loops characterized by $M_{rs}/M_s \rightarrow 1$ tend to a rectangular shape. The remanence ratio depends on the specimen anisotropy and on the domain state. Fully aligned SD particles are characterized by $M_{rs}/M_s = 1$, while non-interacting, uniaxial SD particles are characterized by $M_{rs}/M_s = 0.5$. Large MD particles with unpinned domain walls are characterized by $M_{rs}/M_s \rightarrow 0$, while intermediate values are obtained with VO particles. The hysteresis loop of SP particles is closed, and thus $M_{rs}/M_s = 0$.

Irreversible hysteresis: Fictive major hysteresis loop obtained by selecting only the irreversible magnetization changes (see magnetic irreversibility) along the major hysteresis loop.

Isothermal remanent magnetization (IRM): Remanent magnetization obtained by applying and subsequently removing a direct current (DC) field to a previously demagnetized specimen, while the temperature is kept constant. Only magnetic states that are irreversibly changed by the applied field contribute to the IRM, while the other states maintain the randomly oriented magnetic moments of the original demagnetized state. The saturation remanent magnetization M_{rs} is a particular IRM obtained with a saturating field, that his, a field that is sufficiently strong to irreversibly change all magnetic states.

Magnetic hysteresis: The phenomenon by which the magnetization of a specimen cycled between two fields B_1 and B_2 depends on the direction of the field sweep (from B_1 to B_2 or vice-versa). Magnetic hysteresis arises from irreversible changes of magnetic states in the specimen. The major hysteresis loop is obtained with $B_1 = -B_2 \rightarrow \infty$.

Magnetic reversibility and irreversibility: Ferrimagnetic materials can respond in a reversible or an irreversible manner to changes of the external conditions (e.g., the applied field). A small change of the external conditions produces an irreversible magnetization change if the magnetization does not revert to the original configuration after restoring the original conditions. Total magnetization changes are usually a mixture of reversible and irreversible processes. Magnetic hysteresis is a well-known example of irreversibility: sweeping the applied field between two extreme values produces magnetization changes along two distinct paths, called the ascending and descending branches of the hysteresis loop. Magnetization changes are always reversible in field amplitudes larger than the saturation field. FORC diagrams are two-dimensional representations of mixed reversible and irreversible magnetization changes. Purely reversible systems, such as above the saturation field or in SP particle assemblages, do not contribute to the FORC function.

Magnetic shape anisotropy: The tendency of the spontaneous magnetization of a ferrimagnetic particle to align with the long axis (e.g., in an iron needle).

Magnetic state (or configuration): Describes a specific magnetization configuration inside a ferrimagnetic particle, a group of ferrimagnetic particles, or any other ferrimagnetic system, which is stable against small field changes: application and removal of a small additional external field brings the system back to its original configuration. A transition to another magnetic state occurs when the magnetization change induced by external conditions (e.g., the applied field) becomes irreversible: in this case, restoring the original conditions does not resume the original magnetic state. Examples of magnetic state transitions are those between the two single-domain configurations (parallel and antiparallel) of a uniaxial particle, and between single-domain and vortex configurations. Magnetic state transitions are usually, but not always, characterized by a sudden change of the net magnetic moment. The appearance and disappearance of a magnetic state are often called nucleation and annihilation, respectively.

Magnetic viscosity or thermal relaxation: Describes the delayed response of a magnetic system to any change of the external conditions (typically the applied field). Magnetic viscosity is mainly observed in small SD particles close to the limit where they become superparamagnetic, and, sometimes, in MD particles. The manifestation of viscosity depends on the timescale: the same particles can be stable during laboratory measurements, and viscous over geologic times.

Magnetic vortex (VO): Describes a magnetization whose direction is described by a vortex vector field. In the ideal vortex state, atomic spins are parallel to all free surfaces of the enclosing volume, so that no stray field is produced, and the net magnetic moment is carried only by spins close to the vortex axis (the so-called vortex tube). Vortex states occur in material with weak magnetocrystalline anisotropy, such as magnetite, over a size range between the upper SD stability limit and the lower limit for the nucleation of domain walls (MD).

Magnetization (M): In a magnetic material, it is defined as the total magnetic moment of atomic or molecular spins in an infinitesimal volume element, divided by volume. In the context of magnetic measurements, it is defined as the net magnetic moment of the specimen, normalized by the specimen volume, mass, or area.

Magnetocrystalline anisotropy: The tendency of the spontaneous magnetization of a ferrimagnetic material to align with preferred crystalline axes, called easy axes of magnetization.

Magnetostatic interaction: The influence of the stray field produced by one magnetic particle on another magnetic particle. Interacting magnetic particles are subjected to an internal, so-called interaction field produced by the particles themselves, in addition to the external field applied, for instance, during FORC measurements. Weak magnetostatic interactions, typically occurring between particles separated by several diameters, introduce a small bias to the fields required to promote transitions between magnetic states. In case of random particle assemblages, the internal biasing field is a locally defined random variate that produces a random vertical offset of single particle contributions to the FORC diagram. Therefore, the FORC diagram of weakly interacting particles is a vertically blurred version of the FORC diagram of the same isolated particles without interactions. Strong magnetostatic interactions between closely spaced particles, such as those occurring between magnetosomes in a chain, can produce a strong magnetic coupling that replaces individual magnetic states with a collective state. For instance, isolated magnetosomes possess individual SD behaviors with different switching fields (controlled mainly by crystal elongation), while magnetosomes in a linear chain display a collective SD behavior with a single switching field.

Multi-domain (MD): Domain state used to describe magnetic crystals containing several discrete, homogeneously magnetized domains separated by domain walls. In the absence of external fields, magnetic domains tend to be magnetized in a manner that cancels all stray fields, compatibly with size, shape, and magnetocrystalline anisotropy. In so-called magnetically soft materials, such as magnetite, domain walls can easily move when a small external field is applied. Therefore, the magnetization of magnetically soft MD crystals is typically much less stable than that of SD or VO particles and depends essentially on pinning by crystal defects. The lower MD size limit for magnetite is a few microns, depending on shape and other factors.

Remanent magnetization (M_r): The magnetization of a specimen in a null external field. All ferrimagnetic materials except SP particles can hold a non-zero remanent magnetization that depends on their previous hystory.

Reversal field (B_r): The field at which a FORC measurement starts. Application of B_r to a positively saturated specimen brings the specimen in a mixed magnetic state that reverts to positive saturation while a FORC is measured.

Saturation field (B_s): The field amplitude above which the major hysteresis loop becomes completely closed. Application of B_s converts all magnetic states into a SD-like configuration. It is therefore applied before each FORC measurement to reset the specimen to the same initial magnetic state.

Saturation magnetization (M_s): The magnetization acquired in a magnetic field that is sufficiently large to align all atomic or molecular spin moments. In a ferrimagnetic material, M_s approaches asymptotically the spontaneous magnetization of the material. It is the only type of specimen magnetization that is simply proportional to the amount of magnetic material that is contained in it and is therefore a good indicator of magnetic concentration.

Saturation remanent magnetization (M_{rs}): It is defined as the specimen magnetization in a null external field, which remains after removing a saturation field. It coincides with the zero-field magnetization of the upper and lower branches of the major hysteresis loop. It is usually also the largest remanent magnetization of a specimen in a null field. The ratio M_{rs}/M_s depends on the domain state of particles and the magnetic interactions existing between them. Assemblages of non-interacting, randomly oriented, uniaxial SD particles are characterized by $M_{rs}/M_s = 0.5$. Smaller values are obtained for vortex particles, and $M_{rs}/M_s \rightarrow 0$ in large MD particles containning unpinned domain walls.

Single domain (SD): Domain state describes crystals that are sufficiently small to contain only one magnetic domain. SD particles possess the maximum possible magnetic moment, equal to the product of the spontaneous magnetization and the particle volume. Every particle becomes SD in a saturating external field, regardless of size. Therefore, the domain state of a particle is usually referred to its remanent magnetization.

Stable single domain (SD): Describes SD particles whose magnetization is stable over the experimental time range (seconds to hours for magnetic measurements, millions of years in paleomagnetism). The definition is extended to all systems with same magnetic behavior as SD particles, such as magnetosome chains.

Superparamagnetism (SP): Describes SD particles whose magnetic anisotropy energy is small enough for thermal fluctuations to overcome the energy barrier required to rotate the magnetization between stable orientations (two for a uniaxial particle). Accordingly, superparamagnetic particles do not possess a stable magnetization and do not exhibit an open hysteresis loop. The transition from stable SD to SP depends on temperature and measurement time. The lower stability limit for SD magnetite particles is ~20 nm at room temperature.

Switching field (B_{sw}): The field that is required to nucleate a transition between magnetic states. It is often referred to uniaxial SD particles; in this case, it is the field required to switch between two opposed SD states (parallel and antiparallel to the easy magnetic axis).

Uniaxial Non-interacting single domain (UNISD): UNISD particles represent the simplest model of SD magnetic particles, which has been used to explain the central ridge signature of FORC diagram, as well as other magnetic properties of magnetofossil-rich sediments.

Supporting information: AMB-1 mutant

Deletion plasmid construction and generation of the AMB-1 mutant ΔmamJΔlimJ ΔMIS The MIS deletion construct was created by Gibson assembly. The upstream (AB recombination fragment, 904 bp) and downstream (CD recombination fragment, 842 bp) regions of the islet were amplified by PCR from the AMB-1 genomic DNA with the following primer sets: jwP1A (5'-gaattcctgcagcccgggggatccACTAGTgttcccctccatcacctatac-3') and jwP1B (5'- CCCATCCACTAAATTTAAATAagcggcggtatagcccatg-3') for the AB recombination fragment, jwP1C (5'-TATTTAAATTTAGTGGATGGGctattcgaaccgcct gctc-3') and jwP1D (5'-caccgcggtggcggccgctctagaACTAGTgatagcgagaaccgtcatac-3') for the CD recombination fragment. The Gibson assembly was used to clone AB and CD fragments into the SpeI site (underlined in the above primers) of pAK0 (Komeili et al., 2006), which is a suicide plasmid carrying a kanamycin resistance cassette and a counterselectable *sacB* gene, to generate pAK1121. A spacer (highlighted bold in the above primers) at the end of AB and the beginning of CD fragments was used to connect the AB and CD fragments during Gibson assembly. Δ*mamJ*Δ*limJ*ΔMIS was generated by a two-step recombination method (Murat et al., 2010). The deletion plasmid pAK1121 was conjugated into Δ*mamJ*Δ*limJ* AMB-1 strain (Draper et al., 2011) by using WM3064 as the donor strain. For the first recombination step, the colonies were selected on agar plates containing kanamycin (medium composition given by the ATCC under AMB-1 reference), to obtain the recombinants that have the deletion plasmid integrated into the genomic DNA. For the second recombination step, cells were grown in 10 ml of AMB-1 growth medium without kanamycin, and then counterselected on agar plates containing 2% sucrose, to obtain recombinants that lost the integrated plasmid. These sucrose-resistant colonies were then screened for the absence of MIS by culture PCR with the following primers: jwP2F (5'-catcaccatgaccctgaccg-3') and jwP2R (5'-gacgttttgaa-ggggctggac-3').

Supporting information: micromagnetic calculations

This section explains in detail how synthetic magnetosome chains are constructed, and the methods used for simulating thermally activated transitions between magnetic states and whole FORC measurements. Mathematical symbols used for simulation parameters and other parameters are listed in Table S2 and Table S3 at the end, respectively. Parameter values used for the simulations are listed in Table S1.

1. Magnetosome properties

Micromagnetic simulation of magnetofossils are controlled by two groups of parameters describing the properties of individual crystals on the one hand, and their arrangement in intact or collapsed chains on the other. Relevant crystal properties include size, shape, and, to a minor extent, crystallographic orientation with respect to shape.

1.1 Sizes and shapes

Magnetosomes occur in a variety of complex, strain-specific crystal shapes (Mann et al., 1984, 1987; Sparks et al., 1990; Meldrum et al., 1993a,b; Lefèvre et al., 2011; Clemett et al., 2002). Because magnetic properties of single-domain (SD) magnetite particles are mainly controlled by their elongation, magnetofossils can be subdivided into three main categories for magnetic modeling purposes: (1) equant or isometric crystals, such as cuboctahedra and octahedra, which do not possess a systematic elongation, (2) prismatic crystals, which are moderately elongated, and (3) tooth- or bullet-shaped crystals, which are highly elongated in their mature stage. These three categories form three distinct clusters in a so-called Butler-Banerjee diagram of short-to-long axis ratio vs. length (Fig. S1a). The size and shape distribution within each cluster is determined by different bacterial strains and natural variations within each strain, related to the growth stage or environmental conditions. The apparent elongation of cuboctahedral and octahedral crystals is due in part to projection effects of the TEM micrographs from which shape and size data are retrieved, and in part to random deviation from a perfectly isometric shape.

Magnetofossil sizes are mostly comprised within the limits required for intact magnetosome chain to possess a stable SD-like remanent magnetization, with the magnetic moment of each crystal being parallel to the chain axes and not switchable by thermal activations at room temperature (Muxworthy and Williams, 2006; Newell, 2009). The stability range for isolated crystals is much smaller (Butler and Banerjee, 1975), with a non-negligible proportion of isometric crystals being either too small or too large to carry a stable single-domain remanence without the stabilizing effect of magnetostatic interactions within a chain. Unlike the crystal length, widths within each shape category are almost independent of elongation. Therefore, individual crystal shapes are effectively described by independent width and axis ratio distributions (Fig. S1b-d). These distributions are well approximated by a rescaled versions of the Beta function

$$\mathcal{B}(x;\beta_1,\beta_2)=\frac{\Gamma(\beta_1+\beta_2)}{\Gamma(\beta_1)\Gamma(\beta_2)}x^{\beta_1-1}(1-x)^{\beta_2-1}, \tag{1}$$

where Γ is the Gamma function and $\beta_{1,2}$ two shape parameters.

1.2 Shape axes

The shape of simulated magnetosomes is controlled by three orthogonal axes $(\mathbf{a}_1,\mathbf{a}_2,\mathbf{a}_3)$ with lengths (a_1,a_2,a_3) where $\mathbf{a}_3$ is parallel to the chain axis, up to random deviations from this ideal orientation (Fig. S9). Equidimensional magnetosomes are ideally represented by $a_1=a_2=a_3=s$, where s is the magnetosome width, while ideal elongated magnetosomes are characterized by $a_1=a_2=s$ and $a_3=a_1\lambda$, where $\lambda\geq 1$ is the crystal elongation. An ideal chain is assumed to contain mature magnetosomes with identical, cell-specific widths s_0 and elongations λ_0.

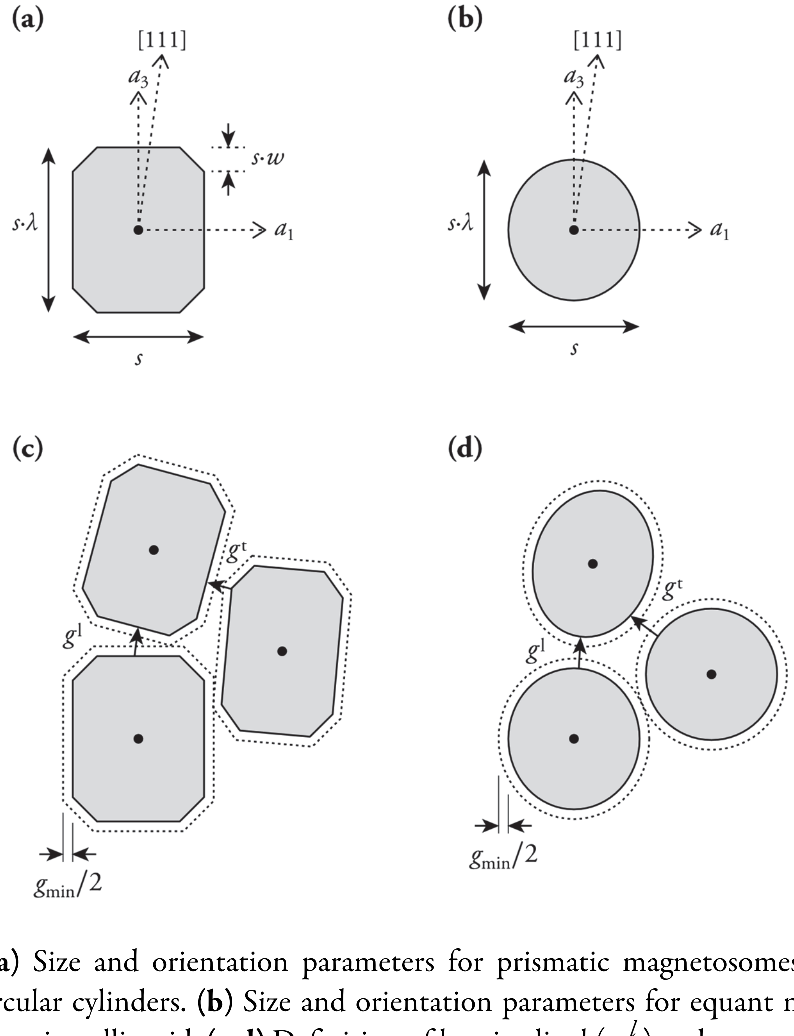

Figure S9: **(a)** Size and orientation parameters for prismatic magnetosomes, modelled as chamfered circular cylinders. **(b)** Size and orientation parameters for equant magnetosomes, modelled as rotation ellipsoid. **(c,d)** Definition of longitudinal (g^l) and transversal (g^t) gaps between prismatic magnetosomes. The dotted line represents the minimum half-gap $g_{min}/2$ between crystals. Longitudinal and transversal gaps are defined as the mean distance between corresponding faces in (c), and as smallest distances in (d).

Magnetosomes in real chains feature small, random variations of their axis orientation and length, relative to the ideal case. In order to reproduce such variations, the orientation of the $\mathbf{a}_3$ -axes is generated with a Fisher-Von Mises distribution

$$\mathcal{F}(\theta;\varkappa_s)=\frac{\varkappa_s}{4\pi\sinh\varkappa_s}\exp(\varkappa_s\cos\theta) \tag{2}$$

where θ is the angle between $\mathbf{a}_3$ and the chain axis, and $\varkappa_s$ a so-called concentration parameter governing the dispersion of θ around the chain axis. Large values of $\varkappa_s$ correspond to small deviations of $\mathbf{a}_3$ from the chain axis, with the standard deviation of θ being proportional to $\varkappa_s^{-1}$ for $\varkappa_s \gg 1$. The other two axes are oriented randomly.

The axes lengths of the n-th magnetosome are given by

$$\begin{aligned} a_{n,1} &= a_{n,2} = (1+\delta x_n)s_0\,t(n) \\ a_{n,3} &= (1+\delta z_n)s_0\,t(n) \end{aligned} \tag{3}$$

where δx_n, δz_n are random Gaussian variates with standard deviation $\sigma_s \ll 1$, limited to ±0.8, and $0 < t(n) \leq 1$ is a size tapering function, which depends on the position of the n-th magnetosome with respect to the closest chain extremity. Cylindrical symmetry is imposed through $a_{i,1} = a_{i,2}$, since the axes are used merely to simulate a uniaxial magnetic anisotropy due to the crystal elongation. The magnetosome shapes are thus controlled by the following set of chain-specific parameters: the crystal width s_0 and elongation e_0 of mature crystals, the dispersion $\varkappa_s$ of long axes orientations with respect to the chain axis, the standard deviation σ_s of random size variations, and the tapering function t.

Shape axes are constructed by applying the following rules to each crystal:

(1) A set of orthogonal axes $(\hat{\mathbf{x}},\hat{\mathbf{y}},\hat{\mathbf{z}})$ is generated, with $\hat{\mathbf{z}}$ being parallel to the chain axis.

(2) The axes are rotated about $\hat{\mathbf{z}}$ by a random angle with uniform distribution between 0 and 2π.

(3) The axes are rotated about a random axis perpendicular to $\hat{\mathbf{z}}$, by an angle generated by a random realization of the Fisher-Von Mises distribution in eq. (2).

(4) The axes lengths are calculated according to eq. (3).

1.3 Crystallographic orientations

Crystal orientation is identified with the [100] magnetite axes. In the case of ideal cuboctahedral or prismatic magnetosomes, it is assumed that one of the [111] axes is parallel to the chain axis, since this is the typical orientation observed for these shapes (Simpson et al., 2005; Pósfai et al., 2013; Körnig et al., 2014). The other [111] axes appear to be randomly oriented (Simpson et al., 2005; Orue et al., 2018). The crystal orientation of simulated magnetosomes is a randomized version of the ideal case, with the [111] axis closest to the chain axis being

governed by a Fisher-Von Mises distribution (eq. 2) with concentration parameter $\varkappa_c$. The crystal axes are generated with the following steps:

(1) A set of orthogonal [100] axes $(\hat{\mathbf{x}},\hat{\mathbf{y}},\hat{\mathbf{z}})$ is generated, with $\hat{\mathbf{x}}=[2^{-1/2},6^{-1/2},3^{-1/2}]$, $\hat{\mathbf{y}}=[-2^{-1/2},6^{-1/2},3^{-1/2}]$, and $\hat{\mathbf{z}}=[0,-(2/3)^{-1/2},3^{-1/2}]$. The principal diagonal of the cube with edges given by $(\hat{\mathbf{x}},\hat{\mathbf{y}},\hat{\mathbf{z}})$ is the [111] axis parallel to $\hat{\mathbf{z}}$.

(2) The axes are rotated about $\hat{\mathbf{z}}$ by a random angle with uniform distribution between 0 and 2π.

(3) In case of elongated magnetosomes, the axes are rotated about a random axis perpendicular to $\hat{\mathbf{z}}$ in the exact same manner as for the shape axes (i.e., using the same rotation axis and rotation angle). This rotation generates the whole crystal misalignment with respect to the chain axis.

(4) The axes are rotated about a random axis perpendicular to $\hat{\mathbf{z}}$, by an angle generated by a random realization of a Fisher-Von Mises distribution with chain-specific concentration parameter $\varkappa_c$. This rotation generates an additional misalignment of the crystalline axes with respect to the shape axes.

1.4 Magnetosome shapes

Magnetosome shape determines the uniaxial magnetic anisotropy used in micromagnetic simulations. This anisotropy is expressed by the so-called demagnetizing tensor $\mathbf{D}$. The calculation of $\mathbf{D}$ is performed through an intermediate step where the real magnetosome shape is approximated by an idealized shape. The idealized shape of equidimensional (cuboctahedral) magnetosomes is a rotation ellipsoid with axes lengths $a_1=a_2$ and a_3. Elongated prismatic magnetosomes are represented by chamfered circular cylinders with diameter $a_1=a_2$ and length a_3. The circular cylinder approximates a hexagonal prism with {110} side faces and {111} top/bottom faces, as typically seen in MV-1 magnetosomes (Weyland et al., 2006). Additional {100} and {111} faces eliminating right angles between side and top/bottom faces (Clemett et al., 2002; Pósfai et al., 2013) are simulated by 45° chamfers of width $s_0 w$, such that the diameter of top/bottom faces is reduced by $2s_0 w$ (Fig. S9). The chamfer width parameter w is subjected to the same randomization as the shape axes length, so that actual chamfer widths are given by $(1+\delta x)s_0 w_0$, where δx is a normally distributed random variate with standard deviation $\sigma_s \ll 1$, limited to ±0.8, and w_0 is the chain-specific mean relative chamfer width.

2. Construction of single-stranded chains

The construction of a single-stranded chain with N magnetosomes begins with the definition of chain-specific parameters that control the characteristics of individual crystals and their arrangement in space (Table S2). Chain-specific parameters for individual crystals are:

the width s_0, the elongation e_0, and the relative chamfer width w_0 (prismatic crystals only) of ideal mature magnetosomes, the concentration parameters $\varkappa_s$ and $\varkappa_c$ of the Fisher-Von Mises distributions used to model shape and crystalline axes orientations with respect to the chain axis, and the standard deviation σ_s of relative variations of axis lengths and chamfer widths. Other parameters control the chain geometry: the chain length, through the number N of magnetosomes and the gaps between them, the off axis magnetosome offsets, the crystal size tapering towards the chain extremities, and the chain bending.

The size tapering of the n-th magnetosome

$$t(n) = s_t + (1 - s_t)\min\left[1, \frac{n-1}{n_t}, \frac{N-n}{n_t}\right] \tag{4}$$

describes a linear increase of the mean crystal width $s_0 t$ from $s_0 s_t$ for the first ($n = 1$) and last ($n = N$) magnetosomes, to s_0 for the $n = n_t + 1$ and $n = N - n_t$ magnetosomes, respectively (Fig. S10).

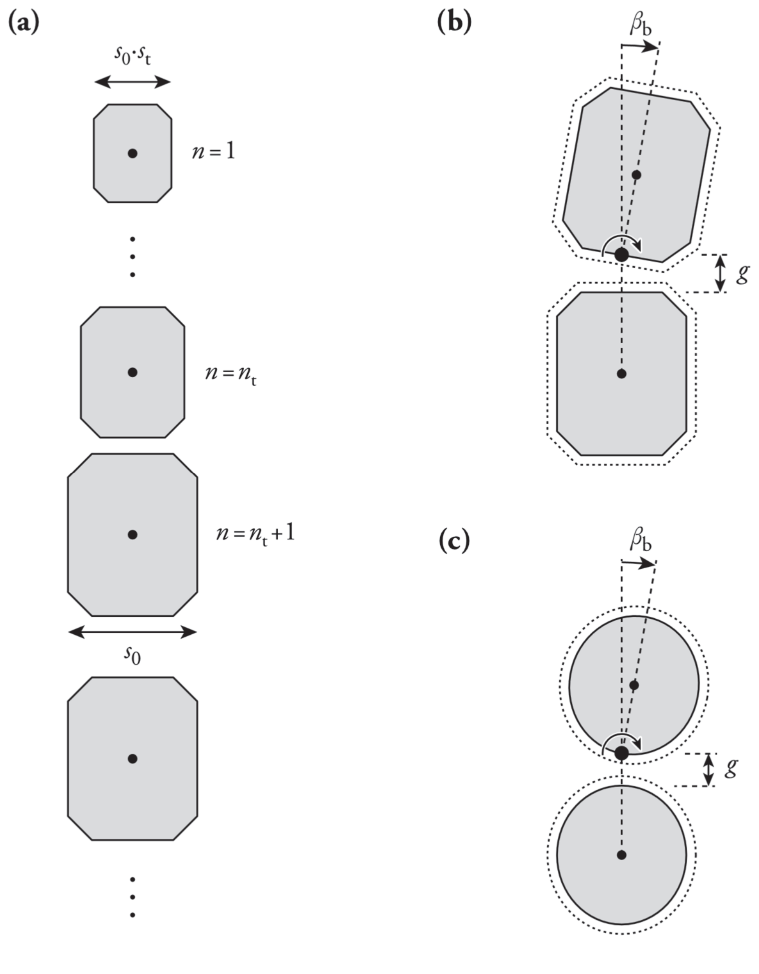

Figure S10: **(a)** Chain tapering geometry. The magnetosome size increases linearly from $s_0 s_t$ for the $n = 1$ magnetosome to s_0 for the $n = n_t + 1$ magnetosome. **(b,c)** Chain bending for prismatic and equant magnetosomes, obtained by rotating the top crystal about the axis marked by the large dot. The bending angle is β_b.

The tapering function is thus controlled by the relative size s_t of the end magnetosomes and the number $n_t \le N/2$ of end magnetosomes affected by tapering.

The arrangement of magnetosomes in a straight chain is controlled by the mean gap g_0 between neighbor crystals, and by the longitudinal and transversal randomization of their positions. Longitudinal gaps are defined as the mean distance between facing cylinder surfaces used to model prismatic crystals (Fig. S9c), or the shortest distance between the ellipsoidal surfaces used to model equant crystals (Fig. S9d). The longitudinal randomization of magnetosome positions causes individual gaps to differ from the expected value g_0, and is realized by defining these gaps as $g=(1+\delta g)g_0$, where δg is a normally distributed random variate with standard deviation $\sigma_z \ll 1$, limited to ± 1. Furthermore, g must be sufficiently large to guarantee a predefined minimum distance $g_{\min} \ge 0$ for the facing surfaces. In intact native chains, the minimum distance is imposed by the thickness of the magnetosome membrane, which has been reported to vary between ~3 and ~7 nm (Gorby et al., 1988; Werckmann et al., 2017; Mickoleit et al., 2018), yielding $g_{\min}$ values comprised between ~6 and ~14 nm, depending on magnetosome size. It is not clear whether the original gap is preserved after cell dissolution, until what remains of the original chains is fixed in the sediment matrix, for instance by adhesion on clay platelets (Galindo-González et al., 2009).

Transversal randomization of the magnetosome positions with respect to the original axial alignment along the z-direction of a cartesian coordinate system is described by distance vectors $\mathbf{d}=(\delta x,\delta y)s_0$ from the chain axis, where δx and δy are normally distributed random variates with standard deviation σ_x.

The straight shape of ideal chains can be altered in several ways, which include bending, kinking, and various forms of collapse that might occur after cell dissolution. Chain bending is the elastic response to small external loads and is commonly observed in intact MTB cells (Shcherbakov et al., 1997; Orue et al., 2018). The maximum elastic bending angle, defined as the angle between segments connecting consecutive crystal centers, is of the order of $g_0/s_0 \approx$ 0.1 (Shcherbakov et al., 1997). Above this limit, kinking occurs, until the chain extremities become close enough to form collapsed structures with magnetic flux closure, such as folded chains (e.g., Fig. 2b in Lins and Farina, 2004), rings, and handles (Kiani et al., 2018). Chain bending is therefore the only deviation of non-collapsed single chains from the ideal straight shape. The typical length scale over which a single magnetosome chain remains straight under the influence of thermal activation is >30 times larger than magnetosome size (Kiani et al., 2015), which means that the maximum value of the bending angle α_b between consecutive magnetosomes is of the order of few degrees. Chain bending is realized by rotating each magnetosome by the bending angle β_b with respect to the previous one, about a rotation axis that intersects the original chain axis while being perpendicular to it and tangential to the crystal surface (Fig. S10b-c). As a result, the mean gap between crystals does not change, as long as the bending angle is sufficiently small to maintain the minimum distance $g_{\min}$ between crystals. Otherwise, the longitudinal gap is increased until this condition is met.

In practice, chain construction is based on the following steps:

(1) Build N magnetosomes as described in Section 1.2, with predefined crystal orientations with respect to a straight chain axis parallel to z.

(2) Define $N-1$ longitudinal gaps using the parameters g_0, $g_{\min}$, σ_z, and taking the magnetosome orientations into account.

(3) Construct a straight chain by placing the center of the first magnetosome at the origin, and subsequently adding the remaining magnetosomes with their centers on the z-axis, and distances along z defined by the $N-1$ gaps calculated in (2). At this stage, the chain axis coincides with the z-axis of the coordinate system and all magnetosome centers lie exactly on the chain axis. Gaps are random realizations of a normal distribution with mean g_0 and standard deviation σ_z, with upper limit $2g_0$ and lower limit set by the minimum distance $g_{\min}$.

(4) Transversal magnetosome positions are randomized by adding $(\delta x, \delta y, 0)$ to the center coordinates of each magnetosome, where δx and δy are normally distributed random variates with standard deviation σ_x.

(6) Chain bending is applied. For this purpose, the straight chain is first rotated randomly about the z-axis. Then, all $n \geq 2$ magnetosomes are rotated by β_b about the y-axis. Next, all $n \geq 3$ magnetosomes are rotated using the same procedure, and so on, until the last magnetosome has been rotated. The bended chain is rotated back about the z-axis by the same azimuthal angle used to prepare chain bending, so that the bending axis orientation is random within the xy-plane.

3. Construction of double-stranded chains

Several types of MTB produce strands of two or more closely arranged magnetosome chains made of equidimensional, prismatic, or tooth-shaped magnetosomes (Spring et al., 1994; Pósfai et al., 2006; Isambert et al., 2007; Faivre and Schüler, 2008; Zhang et al., 2017). In these double- or multi-stranded chains, the arrangement of magnetosomes is staggered, such that crystals of one chain face the gap between consecutive crystals in the other chain. This arrangement is energetically most favorable (Hanzlik et al., 2002; Ruder et al., 2012) to the formation of chain bundles with a consistent magnetic polarity (Simpson et al., 2005). Double-stranded chains can also form after cell death if a single chain is bent beyond the elastic limit: in this case the magnetostatic attraction of the two extremities causes the chain to fold around a kink point (Pósfai et al., 2006). If this type of collapse occurs in a weak field, complete folding produces two parallel chain fragments with opposed magnetic moments that form a closed magnetic loop. Strands with opposed magnetic moments tend to produce a side-by-side arrangement of the crystals that maximizes lateral attractive forces (Varón et al., 2013), as seen on attracted segments of extracted chains (Zhu et al., 2016). Side-by-side arrangement and

size tapering only at one extremity (e.g., Fig. 2b in Kiani et al., 2015) are distinctive features of fold-collapsed chains, which contrasts with those of native double-stranded chains.

The construction of a double-stranded chain of $N = N_1 + N_2$ magnetosomes, with two parallel strands of N_1 and N_2 magnetosomes each, begins with the definition of the magnetosome properties of the two strands and the corresponding longitudinal gaps g_l as in section 1.2. In addition, transversal gaps g_t are used to set the distance between the two sub-chains. Transversal gaps are defined in the same manner as longitudinal gaps, using the parameters g_0, g_{min}, and σ_z. The overall shape of double-stranded chains is governed by two degrees of freedom: bending, as with single chains, and twisting. Twisting consists in the rotation of the two sub-chain axes around a common axis of the double chain. Details of the double chain construction depend on its origin (intact or collapsed), as specified in the following.

3.1 Intact double-stranded chains

Ideal native double-stranded chains consist of two staggered and identically tapered sub-chains of similar length, whose magnetosomes face a common axis at a constant distance nearly equal to that of the longitudinal gaps (Fig. S11a). The longitudinal position of the second strand relative to the first one is defined by the integer lag number l, which describes the lag between the end magnetosomes at one extremity of the double chain in units of magnetosome length. Accordingly, the difference between the z-coordinates of these end magnetosomes is given by $\Delta z = (l + 1/2)(s_0\lambda_0 + g_0)$, where the half-integer factor $l + 1/2$ accounts for the staggered position of magnetosomes in strand 2 facing the gaps between magnetosomes in strand 1. Double-stranded chains can be twisted (Fig. S11b) and bended (Fig. S11c) by rotating and rearranging individual magnetosomes so to maintain, on average, similar distances between crystals. Deviations from the ideal double-stranded chain structure include random variations of magnetosome shape and position, length of the two strands, and longitudinal lag of one strand with respect to the other.

Twisting converts the two straight and parallel strand axes (Fig. S11a) into a double helix whose pitch $2\pi(s_0\lambda_0 + g_0)/\beta_t$ is controlled by the pitch angle β_t, defined as the angle by which the distance vector between the strand axes rotates over the length occupied by a single magnetosome. The helix pitch is much longer than the size of individual magnetosomes, so that a half-turn is typically completed over ~10 magnetosomes or more (Fig. S11b). Twisting requires to rotate the magnetosome centers about the common chain axis, and the magnetosomes about the distance vectors connecting their centers with the chain axis. As with single-stranded chains, magnetosome rotations are performed so to maintain the predefined gaps, compatibly with the given minimum gap g_{min}. Bending of double-stranded chains is more complicated than the single-stranded chain case, because the bending axis might be in-plane with the two sub-chains: in this case, the gap between consecutive magnetosomes lying further

away from the bending axis needs to be increased to maintain the original staggered arrangement (Fig. S11c).

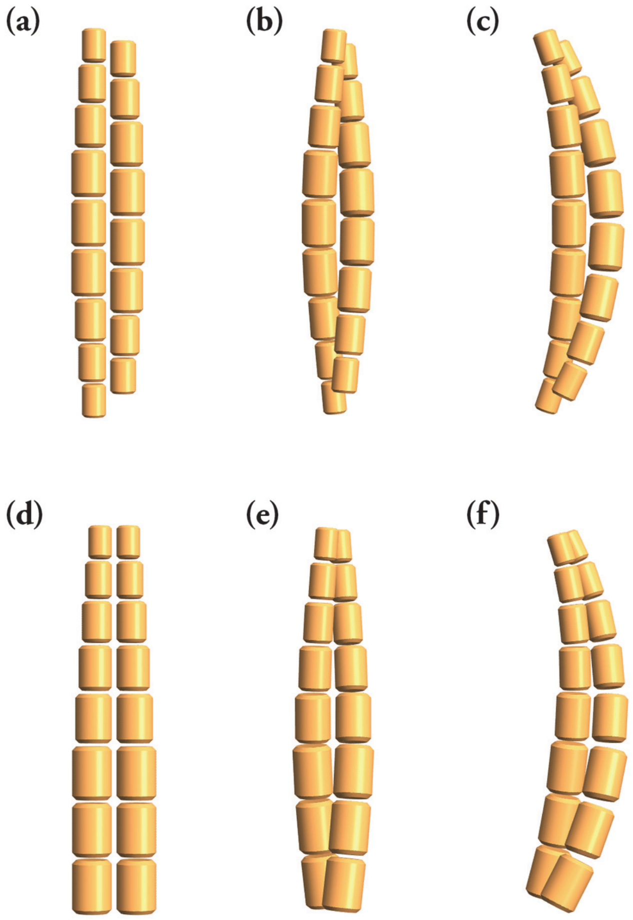

Figure S11: Construction of ideal double-stranded chains by twisting and bending of an initial straight configuration. In these examples, prismatic magnetosomes with elongation e_0 are modeled by chamfered cylinders. **(a)** Straight double-stranded chain of staggered magnetosomes with size tapering at both ends. This example with $N_1 = 9$, $N_2 = 8$, and $l = 0$ is similar to the double chain shown in Fig. 3 of (Simpson et al., 2005). **(b)** Same as (a), after twisting the double chain plane by a half-turn ($\beta_t = 20°$). **(c)** Same as (b), after bending the twisted chain with a bending angle $\beta_b = 6°$, which is close to the elasticity limit calculated in (Shcherbakov et al., 1997). Note the larger gaps on the external bending side (right). **(d-f)** Same as (a-c) for a double-stranded chain obtained by folding a single-stranded, tapered chain of 16 magnetosomes. This example with $N_1 = N_2 = 8$, $N_2 = 8$ is similar to the double-stranded chain shown in Fig. 2 of Lins and Farina (2004).

The construction of staggered double-stranded chains is based on the following steps:

(1) Build N_1 magnetosomes for strand 1 and N_2 magnetosomes for strand 2 as described in Section S1.2, with predefined crystal orientations with respect to a straight chain axis parallel to z and sizes determined by a common tapering function.

(2) Construct N_1- and N_2-vectors of the form $(\delta x, \delta y, 0)$ defining the transversal randomization of the magnetosome positions in the two strands, with δx and δy being normally distributed random variates with standard deviation σ_x.

(3) Define $N_1 - 1$ and $N_2 - 1$ longitudinal gaps using the parameters g_0, $g_{\min}$, σ_z, and taking the magnetosome orientations into account. Define N_1 and N_2 transversal half-gaps with parameters g_0, $g_{\min}$, σ_z, which will be used to set the minimum distance between the two strands.

(4) Construct the first strand using the longitudinal gaps defined in (2). Unlike single-stranded chains, the right crystal edges are aligned with the chain axis, instead of the magnetosome centers through $x_k + (s_k + g_k^{\mathrm{t}})/2 + \delta x_k = 0$, where x_k is the *x*-coordinate of the center of the *k*-th magnetosome in strand 1, δx_k the corresponding transversal randomization, s_k the crystal width, and $g_k^{\mathrm{t}}/2$ the *k*-th transversal half-gap. Magnetosome center coordinates along *y* are defined by $y_k = \delta y_k$ as for single-stranded chains.

(5) Construct the second strand using the longitudinal gaps defined in (2). The left crystal edges are aligned with the chain axis through $x_k - (s_k + g_k^{\mathrm{t}})/2 + \delta x_k = 0$, where x_k is the *x*-coordinate of the center of the *k*-th magnetosome in strand 2, δx_k the corresponding transversal randomization, s_k the crystal width, and $g_k^{\mathrm{t}}/2$ the *k*-th transversal half-gap. Magnetosome center coordinates along *y* are defined by $y_k = \delta y_k$ as for single-stranded chains. The *z*-coordinate of the first magnetosome of strand 2 is identified with that of the midpoint of the gap between the $(l+1)$-th and the $(l+2)$-th magnetosome of strand 1, where the integer $l \geq 0$ is the lag between the two strands.

(6) Because of random variations in magnetosome dimension and gap sizes, magnetosomes are not perfectly staggered. The best possible staggering is obtained by moving strand 2 along *z* until the sum of the squared differences $[z_{2,k} - (z_{1,k+l} + z_{1,k+l+1})/2]^2$ between the longitudinal position of the magnetosomes in strand 2 and the corresponding gap midpoint in strand 1 is minimized. Finally, the overall separation between the two strands is adjusted by adding or subtracting the same offset along *x*, until transversal gaps between corresponding magnetosomes are as small as possible, but larger than the minimum gaps prescribed by the transversal half-gaps $g_k^{\mathrm{t}}/2$ defined in (5).

(7) Chain twisting is applied. The starting point is a double-stranded chain with a straight axis along *z* and magnetosome centers lying in the *xz*-plane, up to small random displacements along *y*. Twisting consists in rotating the center coordinates of all magnetosomes about the *z*-axis by an angle $\beta_{\mathrm{t}} z_{i,k}/(s_0\lambda_0 + g_0)$, where β_{t} is the twisting angle, z_k^i the *z*-coordinate of the *k*-th magnetosome in strand *i*, and $s_0\lambda_0 + g_0$ the mean longitudinal distance between magnetosome centers.

(8) Chain bending is applied. For this purpose, the twisted double-stranded chain is first rotated randomly about the *z*-axis. Then, all $n \geq 2$ magnetosomes in strand 1 and all $n \geq 1 + l$ magnetosomes in strand 2 are rotated by β_{b} about the *y*-axis. The rotation axis is

fixed with respect to the second magnetosome of strand 1 or the $(l+1)$-th magnetosome of strand 2, depending on which of the two is closer to the bending axis. Next, all $n \geq 3$ magnetosomes in strand 1 and all $n \geq 2+l$ magnetosomes in strand 2 are rotated using the same procedure, and so on, until the last magnetosome has been rotated. Finally, the bended chain is rotated back about the z-axis by the same angle used to prepare chain bending, so that the bending axis orientation is random within the xy-plane.

3.2 Fold-collapsed double-stranded chains

The ideal fold-collapsed chain consists of two parallel strands obtained by complete folding of a tapered single-stranded chain (Fig. S11d). Magnetosomes in the two strands are facing each other, whereby this condition is exactly met at the folded side. Tapering occurs only at the other side of the chain, which correspond to the two extremities of the original single-stranded chain. In the ideal case, folding occurs in the middle, so that the number of magnetosomes in the two strands differs at most by 1. The remaining properties of fold-collapsed chains, including twisting, bending, and random deviations from the ideal shape, are defined in the same manner as for native double-stranded chains (section 3.1).

The construction of fold-collapsed chains is based on the following steps:

(1-4) Same as the corresponding steps for staggered double-stranded chains.

(5) Construct the second strand using the longitudinal gaps defined in (2). The left crystal edges are aligned with the chain axis through $x_k - (s_k + g_k^{\mathrm{t}})/2 + \delta x_k = 0$, where x_k is the x-coordinate of the center of the k-th magnetosome in strand 2, δx_k the corresponding transversal randomization, s_k the crystal width, and $g_k^{\mathrm{t}}/2$ the k-th transversal half-gap. Magnetosome center coordinates along y are defined by $y_k = \delta y_k$ as for single-stranded chains. The z-coordinate of the first magnetosome in strand 2 is the same as that of the first magnetosome in strand 1.

(6-7) Chain twisting and bending is applied in the same manner as for staggered double-stranded chains.

4. Energy barrier calculations

The role of chain geometry for the stabilization of high-moment states is investigated by considering idealized chain geometries made of identical, equidimensional magnetosomes with the spontaneous magnetization of magnetite ($\mu_{\mathrm{s}} = 480$ kA/m) and no magnetocrystalline anisotropy. In this case, the total free magnetic energy F in a null field is entirely controlled by point dipole interactions between crystals (Hanzlik et al., 2002). For a chain of N crystals

$$F = \frac{\mu_0}{4\pi} \sum_{k<i} \frac{\mathbf{m}_k \cdot \mathbf{m}_i - 3(\mathbf{m}_k \cdot \hat{\mathbf{r}}_{ki})(\mathbf{m}_i \cdot \hat{\mathbf{r}}_{ki})}{r_{ki}^3}, \tag{5}$$

where $\mathbf{m}_k$ is the magnetic moment vector of the *k*-th magnetosome, r_{ki} the center-to-center distance between magnetosomes *k* and *i*, and $\hat{\mathbf{r}}_{ki}$ the unit vector parallel to the distance vector. In the context of energy barrier calculations, it is convenient to normalize eq. (5) by the energy $k_B T$ of thermal fluctuations at the absolute temperature T. In this case, and for spherical magnetosomes of diameter D separated by a gap gD, eq. (5) becomes

$$\frac{F}{k_B T} = \beta_0 \sum_{k<i} \frac{\hat{\mathbf{m}}_k \cdot \hat{\mathbf{m}}_i - 3(\hat{\mathbf{m}}_k \cdot \hat{\mathbf{r}}_{ki})(\hat{\mathbf{m}}_i \cdot \hat{\mathbf{r}}_{ki})}{(r_{kj}/D)^3}, \tag{6}$$

with $\hat{\mathbf{m}}_k = \mathbf{m}_k / m_k$ being the unit vector indicating the direction of the magnetic moment of the *k*-th magnetosome, and

$$\beta_0 = \frac{\mu_0 \mu_s^2}{4\pi k_B T} \frac{\pi^2 D^3}{36}. \tag{7}$$

In case of magnetite at room temperature ($T = 293$ K), $\beta_0 = 0.0015614 D^3$ if D is expressed in nm.

The thermally activated transition between two stable magnetic states, defined by local minima of eq. (6), is represented by a continuous path Γ connecting the two local minima in the $2N$-dimensional parameter space $\mathbf{x} = (\theta_1, \ldots, \theta_N, \varphi_1, \ldots, \varphi_N)$ formed by the polar angles θ_k and azimuthal angles φ_k of the N magnetic moment vectors. This path is a function of the fractional path length s, with $\Gamma(s=0)$ and $\Gamma(s=1)$ yielding the coordinates of the two local minima. The energy required for a transition along Γ is given by $\max_s \beta(\Gamma(s)) - \beta(\Gamma(0))$. Because the transition probability is strongly dependent on the energy, transitions will occur along paths that minimize the energy increase. These paths are characterized by an energy barrier $\Delta\beta$ that minimizes the energy increase. Finding $\Delta\beta$ requires is equivalent to the search of a global minimum for $\max_s \beta(\Gamma(s)) - \beta(\Gamma(0))$ with respect to all possible paths with same starting and ending points in a $2N$ parameter space. This problem is solved by minimizing the geometric action [(64)]

$$S = \int_0^1 \left\| \dot{\mathbf{x}} + \nabla\beta \right\|^2 \mathrm{d}s \tag{8}$$

of Γ, where $\dot{\mathbf{x}} = \mathrm{d}\mathbf{x}/\mathrm{d}s$ is an infinitesimal change along Γ, and $\nabla\beta$ is the gradient of the normalized free energy given in eq. (6). In practice, eq. (8) is solved numerically starting from an initial path $\Gamma_0 = \mathbf{x}_0$ that is close enough to the final solution and updating this path iteratively with an updating scheme that decreases S. Here, we use the updating scheme

$$\mathbf{x}_{i+1}(s) = \mathbf{x}_i(s) - \alpha_i \left[\nabla\beta(\mathbf{x}_i(s)) - (\nabla\beta(\mathbf{x}_i(s)) \cdot \mathbf{t}_i) \mathbf{t}_i \right], \tag{9}$$

proposed by Fabian and Shcherbakov (2018), where $\mathbf{t}_i(s)$ is the tangential vector of $\mathbf{x}_i$ at the position s along the path, and α_k an empirical step size that is updated at every iteration on the basis of the observed change of $S(\mathbf{x}_i)$. In practice, eq. (9) moves Γ laterally (i.e., perpendicular to $\dot{\mathbf{x}}$) along the direction that decreases the total energy, until $\mathbf{t}_i$ becomes parallel

to the energy gradient. This ensures that the final path contains one or more saddle points that define the height of the energy barrier.

The choice of the initial path is crucial for the convergence of eq. (9) to a global minimum of the energy increase along Γ. Short linear chains of 2-4 spheres reverse by a fanning mechanism (Jacobs and Bean, 1955) given by $\theta_k = (-1)^k \pi s$ and $\varphi_k = 0$, where even and odd magnetic moments rotate clockwise and counterclockwise, respectively, by 180°. In this case, the energy barrier is given by the difference between the interaction energy of dipoles arranged side-by-side and head-to-tail, respectively. This reversal mode yields, to a first approximation,

$$\Delta\beta \approx \beta_0 \frac{3(N-1)}{1+g} \tag{10}$$

if only nearest-neighbor interactions are considered. The energy barrier associated with symmetric fanning grows linearly with chain length and is replaced by more favorable reversal modes for chains containing more than four particles. Newell (2009) found analytically that long single-stranded chains reverse through the nucleation of a reversed domain at one extremity. The reversed domain grows with little additional energy, pushing the boundary between normal and reversed magnetic moments towards the other chain extremity, until the original domain is denucleated. In this case, the energy barrier is mainly controlled by the domain nucleation process and is only marginally affected by the number of magnetosomes when $N > 5$. With these considerations in mind, the initial reversing path for a single-stranded chain is constructed from a two-domain configuration obtained by numerical minimization of eq. (6) with starting parameters $\theta_k = 0$ for $k < n$, $\theta_k = \pi$ for $k > n$, $\theta_n = \pi/2$, and $\varphi_k = 0$, where n denotes the middle magnetosome (Fig. S7a). If necessary (e.g., for $n \neq (N+1)/2$), the two-domain configuration is stabilized by fixing $\theta_n = \pi/2$. The energy associated with this configuration is larger than that of stable states where all magnetic moments are characterized by $\theta_k = 0$ or $\theta_k = \pi$. Relaxation to one of the two stable states is thus obtained by relaxing the two-domain configuration after introducing a little modification of the dipole interaction strength that favor the propagation of the transition zone between the two domains to one of the two extremities. For instance, relaxation to $\theta_k = 0$ can be obtained by multiplying all terms with $k < n$ in eq. (6) by a fixed factor $\eta < 1$. The initial transition path Γ_0 is then obtained by joining the reversed path for the relaxation to $\theta_k = 0$ with the path for the relaxation to $\theta_k = \pi$, realizing a continuous transition from an initial state with $\theta_k = 0$ to a final state with $\theta_k = \pi$ through the previously defined two-domain configuration. Because the relaxation of a two-domain configuration is readily triggered by values of η very close to unit, Γ_0 represents already a good approximation of the true transition path, and convergence of eq. (9) to a global minimum can be expected.

The energy barrier of magnetosome rings and double-stranded chains is calculated in a similar manner. The high-moment state of a large ring of spherical particles, obtained by decreasing a saturating magnetic field to zero, is a two-domain state that divides the ring into two halves with tangential magnetic moments arranged clockwise and counterclockwise,

respectively, separated by two magnetic moments pointing to the same direction along the ring diameter (Fig. S7b). In case of large rings ($N \gg 1$) the domain boundaries can be moved along the circumference with little additional energy. Therefore, the transition to a zero-moment state with clockwise or counterclockwise tangential moments is obtained by reducing the extension of one of the two domains until it is fully denucleated. Double-stranded chains and their magnetic configurations are topologically equivalent to those of a ring of particles "squeezed" by transforming it into an ellipse with unit eccentricity, whose sides coincide with the two strands. Accordingly, the high- and low-moment states of a double-stranded chain are obtained when the two strands contain axial magnetic moment with same and opposite polarity, respectively (Fig. S7c). Magnetic moments at the extremities deviate from the chain axis, due to the stray field produced by the dipolar interactions between the two strands. Migration of one domain boundary in a ring structure away from the symmetric position of the high-moment state is equivalent to the nucleation of a domain boundary in one of the two strands of a double-stranded chain, and subsequent displacement along the chain axis. Therefore, the transition between low- and high-moment states of a double-stranded chains is calculated by relaxation of an intermediate configuration where one of the two stains is divided into two domains as it was the case for a single-stranded chain.

In summary, a similar procedure is used to calculate the zero-field transition between two stable states for all four chain structures considered in this article: single-stranded chains, rings, double-stranded chains with staggered magnetosomes, and fold-collapsed chains. This procedure consists of the following steps:

(1) For all non-ring configurations: construct a transition configuration in which one strand of the chain is divided into two domains by setting $\theta = \pi/2$ for the middle magnetosome and relaxing the remaining magnetic moments.

(2) Calculate the initial transition path by making one of the two domains grow at the cost of the other. This is accomplished by decreasing the strength of dipole interactions within the shrinking domain. Depending on the choice of the shrinking domain, denucleation of this domain yields the high- and the low-moment state.

(3) Extend the transition path calculated in (2) by relaxing the path ends to the final high- and the low-moment configurations after resetting all dipole interaction strengths to their original strength according to eq. (6).

(4) Iteratively update the initial transition path obtained in (3) using eq. (9) until the final transition path is obtained. The corresponding energy barriers for high-to-low and low-to-high moment state transitions are given by $\max_s \beta(\Gamma(s)) - \beta(\Gamma(0))$ and $\max_s \beta(\Gamma(s)) - \beta(\Gamma(1))$, respectively.

5 Micromagnetic FORC simulations

Micromagnetic calculations are needed to obtain the component of the total chain magnetic moment along the direction of the applied field while the applied field changes according to a predefined FORC measurement protocol. These calculations simulate VSM measurements, where the specimen magnetic moment parallel to the applied field direction is measured. Full micromagnetic simulations require each magnetosome to be divided into volume elements whose size does not exceed the exchange length $l_{ex} = (2A_{ex}/\mu_0\mu_s^2)^{0.5}$ (Abo et al., 2013), where A_{ex} and μ_s are the exchange constant and the spontaneous magnetization of the magnetic mineral ($l_{ex} \approx 10$ nm for magnetite). The minimum size for the nucleation of vortex states is $\sim 6 l_{ex}$, which corresponds to ~60 nm in equant magnetite particles (Enkin and Williams, 1994; Newell and Merrill, 2000). The critical size of magnetosomes is 20-50% larger (Fig. S1), due to the stabilizing effect of magnetostatic interactions (Muxworthy and Williams, 2006). Accordingly, vortex states are not nucleated by FORC measurements if the magnetosome width does not exceed 70 nm. In this case, the magnetization of individual crystals is nearly homogeneous, with some deviations resembling a flower bundle appearing between 50 and 70 nm (Berndt et al., 2020).

Regular magnetofossil sizes fall within the stable single-domain limits (Fig. S1), so that their magnetization can be effectively considered homogeneous. In this case, the magnetization of a whole magnetosome is fully described by the bulk magnetic moment vector $\mathbf{m} = \mu_s V \mathbf{u}$, where V is the crystal volume and $\mathbf{u}$ a unit vector representing the direction of $\mathbf{m}$. This simplification greatly accelerates micromagnetic simulations, enabling the calculation of high-resolution FORC diagrams for thousands of magnetosome chains.

5.1 Energy minimization

The magnetic state of a single or double chain containing N magnetosomes is completely specified by the vectors $\boldsymbol{\theta} = (\theta_1, \theta_2, \ldots, \theta_N)$ and $\boldsymbol{\varphi} = (\varphi_1, \varphi_2, \ldots, \varphi_N)$ of polar and azimuthal angles of the magnetic moment orientations $\mathbf{u}_1, \mathbf{u}_2, \ldots, \mathbf{u}_N$, with $\mathbf{u}_i = (\cos\varphi_i \sin\theta_i, \sin\varphi_i \sin\theta_i, \cos\theta_i)$ for $i = 1 \ldots N$. Starting from a well-defined initial state, such as positive saturation at the beginning of each FORC, simulated measurements in the external field $\mathbf{H}$ dictated by the FORC protocol are obtained by successive minimizations of the total free magnetic energy of the chain. Here a distinction is made between the magnetic field $\mathbf{H}$ (SI unit: A/m) and the magnetic induction $\mathbf{B}$ (SI unit: T) improperly identified with the field applied during magnetic measurements through the fixed proportionality $\mathbf{B} = \mu_0 \mathbf{H}$ between the two quantities in air. The free magnetic energy is conveniently normalized by $\mu_0 V_0 \mu_s^2$, where V_0 is the mean volume of mature magnetosomes. The total normalized free energy

$$F = \sum_{i=1}^{N} F_i^{c} + F_i^{s} + F_i^{z} + \sum_{i=1, j>i}^{N} F_{ij}^{int} \tag{11}$$

contains the contributions from magnetocrystalline anisotropy (F_i^{c}), shape anisotropy (F_i^{s}), and Zeeman energy (F_i^{z}) of individual magnetosomes, as well as magnetostatic interaction energies (F_i^{int}) between pairs of crystals. The normalized Zeeman energy in the external field $\mathbf{H} = H\,\mathbf{h}$, where $\mathbf{h}$ is the unit vector representing the field direction, is given by

$$F^{\mathrm{z}} = -\frac{H}{\mu_{\mathrm{s}}}\mathbf{u}\cdot\mathbf{h}\,. \tag{12}$$

The field direction is specified by $\mathbf{h} = (\cos\phi\sin\vartheta, \sin\phi\sin\vartheta, \cos\vartheta)$, where ϑ is the polar angle between the tangent of the chain axis at its midpoint and the field direction, and ϕ the azimuthal angle of the field direction with respect to the chain plane.

The normalized free energy of cubic magnetocrystalline anisotropy is given by

$$F^{\mathrm{c}} = \frac{K_1}{\mu_0\mu_{\mathrm{s}}^2}(\alpha_1^2\alpha_2^2 + \alpha_2^2\alpha_3^2 + \alpha_3^2\alpha_1^2) + \frac{K_2}{\mu_0\mu_{\mathrm{s}}^2}\alpha_1^2\alpha_2^2\alpha_3^2\,, \tag{13}$$

where $\alpha_k = \mathbf{c}_k\cdot\mathbf{u}$ is the angle between the magnetization direction $\mathbf{u}$ and the k-th (100) axis $\mathbf{c}_k$, of the crystal, and K_1, K_2 are the cubic anisotropy constants: $K_1 = -13.5\ \mathrm{kJ/m^3}$ and $K_2 = -2.8\ \mathrm{kJ/m^3}$ for magnetite at room temperature (Kakol and Honig, 1989; Aragón, 1992). Magnetostriction is negligible for the size range of magnetosomes (Fabian and Heider, 1996) and is therefore ignored.

The normalized free energy of shape anisotropy, or demagnetizing energy, is given by

$$F^{\mathrm{s}} = \mathbf{u}\cdot\mathbf{D}\cdot\mathbf{u}\,, \tag{14}$$

where $\mathbf{D}$ is the magnetometric (i.e., volume-averaged) demagnetizing tensor (Fukuma and Dunlop, 2006) of a homogeneously magnetized body with given shape. The demagnetizing tensor is defined so, that $\mathbf{D}\cdot\mathbf{M}$ is the volume-averaged internal field caused by the homogeneous magnetization $\mathbf{M}$. Analytical solutions exist for ellipsoids and cylinders. In case of a triaxial ellipsoid with principal axes $a_3 \ge a_2 \ge a_1$, the internal field is homogeneous, and the demagnetizing tensor has the principal components (Osborn, 1945)

$$\begin{aligned}
D_1 &= \frac{a_3a_2a_1}{(a_3^2 - a_2^2)\sqrt{a_3^2 - a_1^2}}\left[F(\gamma,k) - E(\gamma,k)\right] \\
D_2 &= \frac{a_3^2a_2a_1}{(a_3^2 - a_2^2)\sqrt{a_3^2 - a_1^2}}\left[\frac{a^2 - c^2}{b^2 - c^2}E(\gamma,k) - F(\gamma,k)\right] - \frac{a_1^2}{a_2^2 - a_1^2}\,, \\
D_3 &= \frac{a_2}{a_2^2 - a_1^2}\left[a_2 - \frac{a_3a_1}{\sqrt{a_3^2 - a_1^2}}E(\gamma,k)\right]
\end{aligned} \tag{15}$$

with $D_1 + D_2 + D_3 = 1$ in SI units, where

$$F(\gamma,k)=\int_0^{\gamma}\frac{\mathrm{d}u}{\sqrt{1-k^2\sin^2 u}}$$

$$E(\gamma,k)=\int_0^{\gamma}\sqrt{1-k^2\sin^2 u}\,\mathrm{d}u \tag{16}$$

are the incomplete elliptic functions of the first and second kind, respectively, and

$$\gamma=\arccos\frac{a_1}{a_3},\quad k=\sqrt{\frac{a_3^2-a_2^2}{a_3^2-a_1^2}} \tag{17}$$

In the particular case of prolate rotation ellipsoids, which are used to model equidimensional magnetosomes, the limit of eq. (15) for $a_2-a_1\to 0$ yields

$$D_3=\frac{1}{\lambda^2-1}\left[\frac{\lambda}{2\sqrt{\lambda^2-1}}\ln\frac{\lambda+\sqrt{\lambda^2-1}}{\lambda-\sqrt{\lambda^2-1}}-1\right] \tag{18}$$

and $D_1=(1-D_3)/2$ with $\lambda=a_3/a_1$.

The demagnetizing tensor of a circular cylinder has two identical principal components perpendicular to the cylinder axis, and a third principal component (Joseph, 1966; Chen and Brug, 1991)

$$D_3=\frac{1}{3}-\frac{2}{3}\left[\frac{2}{\pi}\left[(1+\lambda^2)K(-1/\lambda^2)+(1-\lambda^2)E(-1/\lambda^2)-1/\lambda\right]-1\right] \tag{19}$$

parallel to the cylinder axis, with $K=F(\pi/2,\cdot)$ and $E=E(\pi/2,\cdot)$ being the complete elliptic integrals of the first and second kind, respectively, and λ the length-to-diameter ratio ‡. The demagnetizing tensor of chamfered cylinders cannot be resolved analytically; however, the effect of chamfering on D_3 does not exceed 10% for the usual geometries required to approximate prismatic magnetosomes. Therefore, excellent numerical approximations can be obtained using the following empirical correction factor:

$$\frac{D_3(\lambda,w)}{D_3(\lambda,0)}=(1+2.534w^{2.798}-0.029w)(1+1.435w^2 f_1 f_2)$$

$$f_1=\tanh\left\{\left[2.5\tanh^4(24w)+(80w)^4 e^{-59.5w}\right]^2\left[\lambda-(0.452+2.568w^{1.683})^2\right]\right\}, \tag{20}$$

$$f_2=\exp\left[-(0.779+0.43e^{-8.632w})^2(\lambda-1)\right]-1$$

‡ The original result reported in eq. (11) of Joseph (1966) is incorrect. It has been recalculated using the integral definition in eq. (9) of the same reference, and the corrected result has been verified by comparison with numerical results from Table 1 in Chen and Brug (1991).

where $D_3(\lambda,w)$ is the longitudinal demagnetizing factor of a cylinder with length-to-diameter ratio λ and normalized chamfering width w, and $D_3(\lambda,0)$ longitudinal demagnetizing factor of the same cylinder without chamfering, as given by eq. (19) (Fig. S12). The maximum error of this approximation is <1% for the typical ranges of λ and w that characterize prismatic magnetosomes.

The interaction energy between two magnetized particles 1 and 2 is equivalent to the sum of the potential energies

$$E_{12} = \frac{\mu_0}{4\pi} \frac{q_1(\mathbf{r}_1) q_2(\mathbf{r}_2)}{|\mathbf{r}_2 - \mathbf{r}_1|} \tag{21}$$

of all pairs of magnetic charges (q_1, q_2) carried by surface elements of the two particles, localized by the position vectors $\mathbf{r}_1$ and $\mathbf{r}_2$, respectively. For particle i with magnetization $\mathbf{M}_i$, the surface charge carried by a surface element $\mathrm{d}S_i$ with normal vector $\mathbf{n}_i$ is given by $q_i = \mathbf{M}_i \cdot \mathbf{n}_i \, \mathrm{d}S_i$. The normalized energy of two homogeneously magnetized particles with same spontaneous magnetization μ_s and magnetic moments parallel to $\mathbf{u}_1$ and $\mathbf{u}_2$, respectively, is then given by

$$F_{12}^{\mathrm{int}}(\mathbf{u}_1, \mathbf{u}_2) = \frac{1}{4\pi V_0} \oiint_{S_1, S_2} \frac{(\mathbf{u}_1 \cdot \mathbf{n}_1)(\mathbf{u}_2 \cdot \mathbf{n}_2)}{|\mathbf{r}_2 - \mathbf{r}_1|} \mathrm{d}S_1 \mathrm{d}S_2 \,. \tag{22}$$

Using $\mathbf{u}_i = u_{i1}\mathbf{e}_1 + u_{i2}\mathbf{e}_2 + u_{i3}\mathbf{e}_3$ with respect to a common coordinate system with basis vectors $(\mathbf{e}_1, \mathbf{e}_2, \mathbf{e}_3)$, eq. (22) can be expressed as

$$F_{12}^{\mathrm{int}}(\mathbf{u}_1, \mathbf{u}_2) = \sum_{i,j=1}^{3} F_{ij} (\mathbf{u}_1 \cdot \mathbf{e}_i)(\mathbf{u}_2 \cdot \mathbf{e}_j) \tag{23}$$

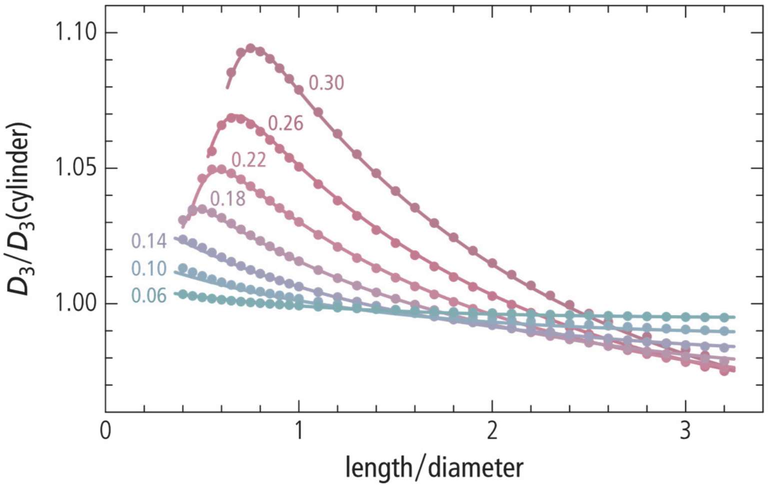

Figure S12: Ratio between the demagnetizing factors of chamfered and regular cylinders of given length-to-diameter ratio along the cylinder axis (dots). Numbers indicate the normalized chamfer width w. Lines represent the empirical correction factor given by eq. (20).

with coefficients

$$F_{ij} = \frac{1}{4\pi V_0} \oiint_{S_1,S_2} \frac{(\mathbf{e}_i \cdot \mathbf{n}_1)(\mathbf{e}_j \cdot \mathbf{n}_2)}{|\mathbf{r}_2 - \mathbf{r}_1|} \mathrm{d}S_1 \mathrm{d}S_2 \tag{24}$$

of the interaction matrix $\mathbf{F}_{1,2}$.

In case of equidimensional magnetosomes, each particle is represented by a rotation ellipsoid with diameter s_i and elongation λ_i, whose stray field coincides with that of a centered point dipole with same dipole moment vector. In this case, eq. (22) yields the well-known expression for point dipole interactions, that is

$$F_{12}^{\text{int}} = \frac{1}{24}\left(\frac{s_1 s_2}{s_0}\right)^3 \frac{\lambda_1 \lambda_2}{\lambda_0} \frac{\mathbf{u}_1 \cdot \mathbf{u}_2 - 3(\mathbf{u}_1 \cdot \mathbf{r}_{12})(\mathbf{u}_2 \cdot \mathbf{r}_{12})/r_{12}^2}{r_{12}^3} \tag{25}$$

with distance vector $\mathbf{r}_{12}$. The case of prismatic magnetosomes is more complicated, requiring the explicit evaluation of eq. (24) on the chamfered cylinder surfaces. Each magnetosome pair is represented by two circular cylinders with radii R_i, lengths L_i, and chamfers of widths $W_i = 2R_i w_i$ in a coordinate system $(\hat{\mathbf{x}},\hat{\mathbf{y}},\hat{\mathbf{z}})$ centered on cylinder 1, such that the center of cylinder 2 is given by $\mathbf{C}_2 = (x_2, 0, z_2)$. Furthermore, let $(\mathbf{a},\mathbf{b},\mathbf{c})$ be the unit vectors representing the principal axes of the second cylinder in the $(\hat{\mathbf{x}},\hat{\mathbf{y}},\hat{\mathbf{z}})$ coordinate system. The flat faces of the two cylinders, together with their chamfers, are defined by

$$\begin{aligned}
\mathfrak{F}_1(r_1,\varphi_1) &= \begin{cases} r_1\hat{\mathbf{x}}\cos\varphi_1 + r_1\hat{\mathbf{y}}\sin\varphi_1 \pm \dfrac{L_1}{2}\hat{\mathbf{z}}, & 0 \le r_1 \le R_1 - w_1 \\ r_1\hat{\mathbf{x}}\cos\varphi_1 + r_1\hat{\mathbf{y}}\sin\varphi_1 \pm \left(\dfrac{L_1}{2} + R_1 - W_1 - r_1\right)\hat{\mathbf{z}}, & R_1 - W_1 \le r_1 \le R_1 \end{cases} \\
\mathfrak{F}_2(r_2,\varphi_2) &= \begin{cases} \mathbf{C}_2 + r_2\mathbf{a}\cos\varphi_2 + r_2\mathbf{b}\sin\varphi_2 \pm \dfrac{L_2}{2}\mathbf{c}, & 0 \le r_2 \le R_2 - w_2 \\ \mathbf{C}_2 + r_2\mathbf{a}\cos\varphi_2 + r_2\mathbf{b}\sin\varphi_2 \pm \left(\dfrac{L_2}{2} + R_2 - W_2 - r_2\right)\mathbf{c}, & R_2 - W_2 \le r_2 \le R_2 \end{cases}
\end{aligned}, \tag{26}$$

where positive and negative signs apply to the top and bottom faces, respectively, and the lateral surfaces by

$$\begin{aligned}
\mathfrak{C}_1(\varphi_1,\zeta_1) &= R_1\hat{\mathbf{x}}\cos\varphi_1 + R_1\hat{\mathbf{y}}\sin\varphi_1 + \zeta_1\hat{\mathbf{z}}, & W_1 - \frac{L_1}{2} \le \zeta_1 \le \frac{L_1}{2} - W_1 \\
\mathfrak{C}_2(\varphi_2,\zeta_2) &= \mathbf{C}_2 + R_2\mathbf{a}\cos\varphi_2 + R_2\mathbf{b}\sin\varphi_2 + \zeta_2\mathbf{c}, & W_2 - \frac{L_2}{2} \le \zeta_2 \le \frac{L_2}{2} - W_2
\end{aligned}. \tag{27}$$

Using this description of the cylinder surfaces, the interaction matrix is given by

$$\mathbf{F}=\mathbf{F}(\mathfrak{F}_1^+,\mathfrak{F}_2^+)+\mathbf{F}(\mathfrak{F}_1^+,\mathfrak{F}_2^-)+\mathbf{F}(\mathfrak{F}_1^+,\mathfrak{C}_2)+$$
$$\mathbf{F}(\mathfrak{F}_1^-,\mathfrak{F}_2^+)+\mathbf{F}(\mathfrak{F}_1^-,\mathfrak{F}_2^-)+\mathbf{F}(\mathfrak{F}_1^-,\mathfrak{C}_2)+, \quad (28)$$
$$\mathbf{F}(\mathfrak{C}_1,\mathfrak{F}_2^+)+\mathbf{F}(\mathfrak{C}_1,\mathfrak{F}_2^-)+\mathbf{F}(\mathfrak{C}_1,\mathfrak{C}_2)$$

where positive and negative signs apply to the top and bottom faces of $\mathfrak{F}_{1,2}$, respectively, and with matrix coefficients

$$F_{ij}(S_1,S_2)=\frac{1}{\pi^2\mu_0\mu_s^2V_0\lambda_0}\iint_{\mathbf{r}_k\in S_k}\frac{(\mathbf{e}_i\cdot\mathbf{n}_1)(\mathbf{e}_j\cdot\mathbf{n}_2)}{|\mathbf{r}_2-\mathbf{r}_1|}\mathrm{d}S_1\mathrm{d}S_2 \quad (29)$$

where $S_{1,2}$ are the surfaces specified in eq. (26-27). The matrix coefficients in eq. (29) need to be calculated only once for a complete FORC simulation of a given chain configuration, since they depend only on geometric factors. Interaction coefficients are very sensitive to the geometry, relative position and orientation of nearest neighbor magnetosomes, while converging to the dipolar approximation of eq. (25) over longer ranges.

5.2 FORC simulations

FORC simulations of individual magnetosome chains are made of few curves (Fig. S13) from which the whole set of measurements corresponding to a complete FORC protocol is reconstructed. This simplification is possible because isolated chains possess only few magnetic states. Each simulated FORC starts in a positive saturating field H_{sat} that is sufficiently large for all chains to possess only one stable magnetic state. Next, the field is decreased in small regular steps δH corresponding to the resolution of the FORC simulation. At each step, a new state is calculated using the $\boldsymbol{\theta}=(\theta_1,\theta_2,\ldots,\theta_N)$ and $\boldsymbol{\varphi}=(\varphi_1,\varphi_2,\ldots,\varphi_N)$ values of the previous state as starting parameters for the energy minimization. This operation is repeated until the minimum reversal field $H_{\mathrm{r}}^{\min}$ specified by the FORC protocol is reached. For a complete FORC simulation, the amplitude of $H_{\mathrm{r}}^{\min}$ needs to exceed the field $H_{\mathrm{r,max}}$ above which the major hysteresis loop closes (Fig. S13). The magnetic state calculated for each applied field step defines the component

$$m_{\|}(H)=\mu_s\mathbf{h}\cdot\sum_{i=1}^{N}(\sin\theta_i\cos\varphi_i,\sin\theta_i\sin\varphi_i,\cos\theta_i)V_i \quad (30)$$

of the magnetic moment parallel to the positive applied field direction, which simulates the contribution of a single chain to the bulk measurement.

Because calculations start at a positive saturating field, the pairs $(H_i,m_{\|}(H_i))$ build, by definition, a numerical simulation of the descending branch of the major hysteresis loop. Magnetic state changes between consecutive field steps are mostly reversible, except for few cases where the currently occupied local energy minimum disappears, and a new state is nucleated at a different energy minimum. These are the only places along the descending

hysteresis branch that give raise to consecutive, non-overlapping FORCs (Fig. S13). Accordingly, FORCs need to be calculated only for the few reversal fields immediately preceding a magnetic state transition.

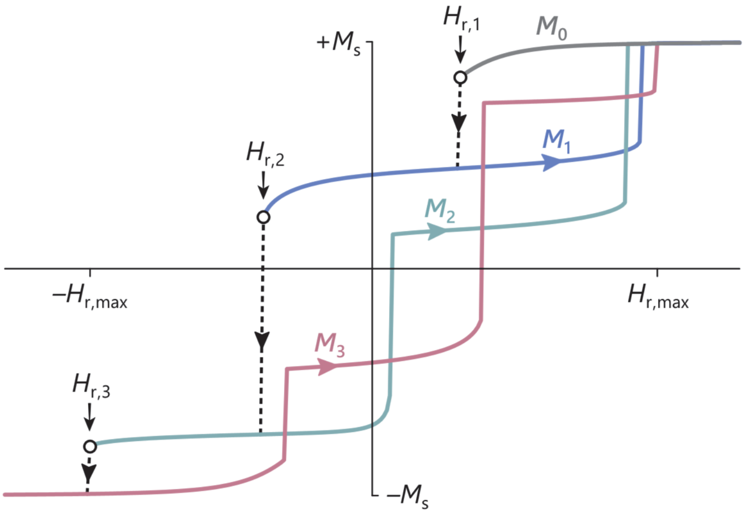

Figure S13: Micromagnetic FORC simulation of a double-stranded magnetosome chain. The solid and dashed lines connecting the open circles labeled $H_{r,1}$ through $H_{r,3}$ build the reversible and irreversible segments of the descending branch of the major hysteresis loop. Irreversible transitions between magnetic states occur at the three reversal fields $H_{r,1}$, $H_{r,2}$, and $H_{r,3}$. FORCs labeled M_0 through M_3 start at reversal fields immediately preceding a magnetic state transition on the descending hysteresis branch. All FORCs starting at reversal fields $H_r > H_{r,1}$ overlap exactly with M_0; all FORCs starting at $H_{r,1} \le H_r < H_{r,2}$ overlap exactly with M_1, and so on. Finally, all FORCs starting at $H_r < -H_{r,max}$ overlap exactly with M_3, which is the ascending branch of the major hysteresis. $H_{r,max}$ is the maximum field amplitude of irreversible magnetic state transitions along the major hysteresis loop. Accordingly, the major loop closes at $\pm H_{r,max}$.

In most cases, magnetic state transitions produce a discontinuity in the measured magnetization; however, this is not always the case, as seen for instance with Stoner-Wohlfarth particles at specific orientations with respect to the applied field (Stoner and Wohlfarth, 1948). Furthermore, small discontinuities might not be detected by the finite field steps used for the simulations. Therefore, irreversibilities along the descending branch are detected by reversing each field step. For instance, irreversibility between the k-th and the $(k+1)$-th point of the descending branch is detected by increasing the applied field H_{k+1} by a single step δH, starting with the magnetic state of the $(k+1)$-th point: if the resulting magnetic state at $H_{k+1} + \delta H = H_k$ does not coincide with that of the descending branch at the same field, a new FORC curve is calculated, starting from $H_r = H_{k+1}$ and increasing the applied field in steps of δH until the maximum field specified by the FORC protocol is reached.

FORC simulations of individual chains based on the abovementioned principles are calculated as follows. First, a maximum field H_{max} is defined: this field should be at least as large as the saturation field H_s, defined as the minimum field in which any chain configuration possess a single energy minimum in $\pm H_s$. For defect-free magnetic materials with weak magnetocrystalline anisotropy, $H_s \approx \mu_s/2$ (e.g., 0.3 T for magnetite), regardless of particle geometry and domain state. Simulations for this article have been performed using $H_{max} =$ 0.35 T. Next, the descending branch of the major hysteresis loop is calculated between $+H_{max}$ and $-H_{max}$ in steps of $-\delta H$, the resolution of FORC simulations, storing the magnetic states corresponding to each applied field. The reversibility of each field step is checked by taking each of the stored states and verifying that increasing the field by $+\delta H$ produces a magnetization change along the descending branch. There will be a limited number $p \ge 1$ of field values $H_{r,1},\dots,H_{r,p}$, for which this condition is violated. The magnetic states at $H_{r,1}+\delta H,\dots,$ $H_{r,p}+\delta H, -H_{max}$ represent the starting points for the calculation of $p+1$ selected FORCs in steps of δH, until the maximum field H_{max} is reached. This set of selected FORCs builds the so-called FORC core, which is then used to calculate complete FORC sets. A complete set of FORCs starting at H_r values ranging from $+H_{max}$ to $-H_{max}$ in steps of $-\delta H$ is constructed by taking the $[H_r, H_{max}]$ range of the first selected FORC that begins at a reversal field $\le H_r$. The classic FORC protocol (Pike et al., 1999) is a subset of simulated FORCs with $H_u^{max} \le H_{max} - H_c^{max}$, $H_u^{min} \ge -H_{max} + H_c^{max}$, and $H_c^{max} \le H_{max}$.

5.3 Implementation and parameter choice

The FORC simulation algorithms described in previous sections have been implemented in a Wolfram Mathematica package called `MagnetosomeChainSimulator`. The package functions `SingleChainForcSimulation` and `DoubleChainForcSimulation` calculate the FORC cores of individual single-stranded and double-stranded chains, respectively, with control parameters listed in Table S3. Stored lists of single or double chain results are merged into a single set of simulated FORC measurements using the `FORCMerger` function and exported to a special data format for further processing with VARIFORC (Egli, 2013) using the `FORCExporter` function.

`SingleChainForcSimulation` and `DoubleChainForcSimulation` enable a detailed control of the mean magnetosome and chain geometry, as well as the randomization of the mean parameters. The chosen set of parameters (Table S1) aims at reproducing the typical appearance of magnetofossils and single- or double-stranded chain geometries of wild-type magnetotactic bacteria (Fig. S2-S5). The in-situ geometry of intact and collapsed magnetofossil chains is largely unknown, because the magnetic extraction procedure employed to prepare samples for TEM observations destroys the original arrangement of magnetosomes. Magnetic extracts contain large, disordered magnetosome clusters, which might or might not retain, at least partially, fragments of the original chains (Vali et al., 1987; Kobayashi et al., 2006; Chen et al., 2007; Li et al., 2012). The dissolution of stabilizing biological structures, such as

magnetosome membranes, favor the formation of denser clusters (Lang and Schüler, 2006; Chen et al., 2007), while the application of large magnetic fields to aqueous suspensions, such as during magnetic extraction, promotes the formation of particle strings resembling magnetosome chains (Zhang et al., 2005; Barrett et al., 2011; Wang et al., 2013). On the other hand, the complete collapse of magnetosome chains in natural environments might be prevented by spontaneous adhesion of magnetite to larger sediment particles (Tombácz et al., 2001; Galindo-González et al., 2009).

Simulations in this article are limited to six types of hypothetical magnetofossil configurations that might be encountered in natural sediment. The first four types include intact single and double chains of equant and prismatic magnetosomes with size and shape distributions taken from 3441 magnetite crystals identified as magnetofossils (Fig. S1). The last two types include double chains of equant and prismatic magnetosomes resulting from the collapse of single chains when kinked beyond their elastic limit. This form of collapse is expected to occur in nature, since it is triggered by minor lateral forces that can be expected to occur in bioturbated sediment once the cell material has been dissolved. Other forms of collapse induced by specific treatments used for TEM observation, such as clumping (Kobayashi et al., 2006) and shrinkage (Fig. 9 in Shcherbakov et al., 1997) are not considered, since they result from the application of strong mechanical and magnetic forces to aqueous suspensions, which are not present in nature.

Chain folding, however, is not necessarily the only type of chain alteration that might occur during early sediment diagenesis. The complete collapse of isolated chains might produce isolated small magnetosome clusters similar to those produced by the ΔmamJ mutant of *M. gryphiswaldense*; however the small remanence ratio of these clusters ($M_{rs}/M_s \approx 0.23$, Katzmann et al., 2013), which is typical for dense clusters of single-domain magnetite (Muxworthy et al., 2003), is incompatible with typical magnetofossil values (Ludwig et al., 2013). Therefore, significant contributions from this form of total collapse can be excluded. Other forms of alterations of the original chain structure include fragmentation, that is, the subdivision into shorter chain fragments. Certain magnetotactic bacteria strains, such as MV-1 (Kalirai et al., 2013) produce chains containing large gaps, at least under fast growing conditions typical of cultures. Subsection of those chains might be easily ripped apart after cell dissolution, through adhesion to different sediment particles. Fragmentation is thus expected to decrease the mean length of magnetofossil chains. The abundance of short chain fragments in magnetofossil extracts might reflect this fragmentation process, but it might also be an artifact of the sample preparation procedure for TEM observations. For modeling purposes, fragmented chains differ from intact chains only by the number of magnetosomes. The effect of chain length on bulk magnetic properties is large for chains fragments containing 2 or 3 magnetosomes and becomes negligible with 6 or more crystals (Chang et al., 2019). A minimum of 4 magnetosomes has been assumed for single chains or chain fragments, based on the typical minimum length of natural sections of chains containing large gaps (Meldrum et al., 1993b; Bazylinski et al., 1995; Kalirai et al, 2013; Blondeau et al., 2018; Monteil et al., 2018; Zhu et al., 2016).

The maximum length of simulated single chains is limited to 9 magnetosomes since longer chains are magnetically undistinguishable. A uniform chain length distribution between these two limits is assumed (Table S1).

The other parameters are chosen according to the criteria discussed in Sections 1–3, so that the generated chains resemble TEM observation reported in the literature, with respect to the average shape and random deviations from this shape (Table S1). Random examples of double-stranded chains generated with these parameters are shown in Fig. S2–5. For each of the abovementioned six chain configuration, ~10^5 examples like those in Fig. S2–5 have been generated, and for each of these examples, a single set of FORC measurements has been simulated assuming a pure magnetite composition, random orientation with respect to the applied fields, a maximum applied field of 0.35 T, and 1 mT field steps.

The full FORC simulation of a single-stranded chain takes ~4 min on average in case of equant magnetosomes, and ~11 min in case of prismatic magnetosomes, on a single processor core running at 2.7 GHz. The longer time required for prismatic magnetosomes is due to the more complex handling of magnetostatic interactions when the point dipole approximation cannot be used. Double-stranded chains take a significant longer time: 4.5 and 7.6 hours on average for equant and prismatic magnetosomes respectively, under the same conditions, due to the larger number of particles and nearest neighbor magnetostatic interactions to account for. The total CPU time of ~3×10^6 hours·core·GHz required to complete all simulations has been shared among 6 desktop and 20 Raspberry Pi 4 computers.

Results obtained for individual chains have been merged into a single set of simulated FORC measurements with unit saturation magnetization ($M_s = 1$) for each of the six configurations (Fig. S6). Simulated measurements are apparently continuous; however, they contain small magnetization jumps from a finite number of individual chain contributions of the type shown in Fig. S14. Statistical fluctuations in the contribution of these jumps to the bulk magnetization become visible in the finite difference estimate $\rho = \delta M/(\delta B)^2$ of the FORC function (Fig. S14). Therefore, FORC diagrams have been calculated with the same variable smoothing procedure VARIFORC (Egli, 2013) used to process noisy measurements. The smoothing factor $S = 5 + 0.07B/\delta B$, where B is the FORC diagram coordinate and $\delta B = 1$ mT measurement resolution, with a limitation to $S = 2$ along the central ridge, and to $S = 9$ at places with maximum curve slope suppress the statistical noise of simulated measurements while preserving high-resolution features such as the central ridge.

The full FORC simulation of a single chain takes ~4 min on average in case of equant magnetosomes, and ~11 min in case of prismatic magnetosomes, on a single processor core running at 2.7 GHz. The longer time required for prismatic magnetosomes is due to the more complex handling of magnetostatic interactions when the point dipole approximation cannot be used.

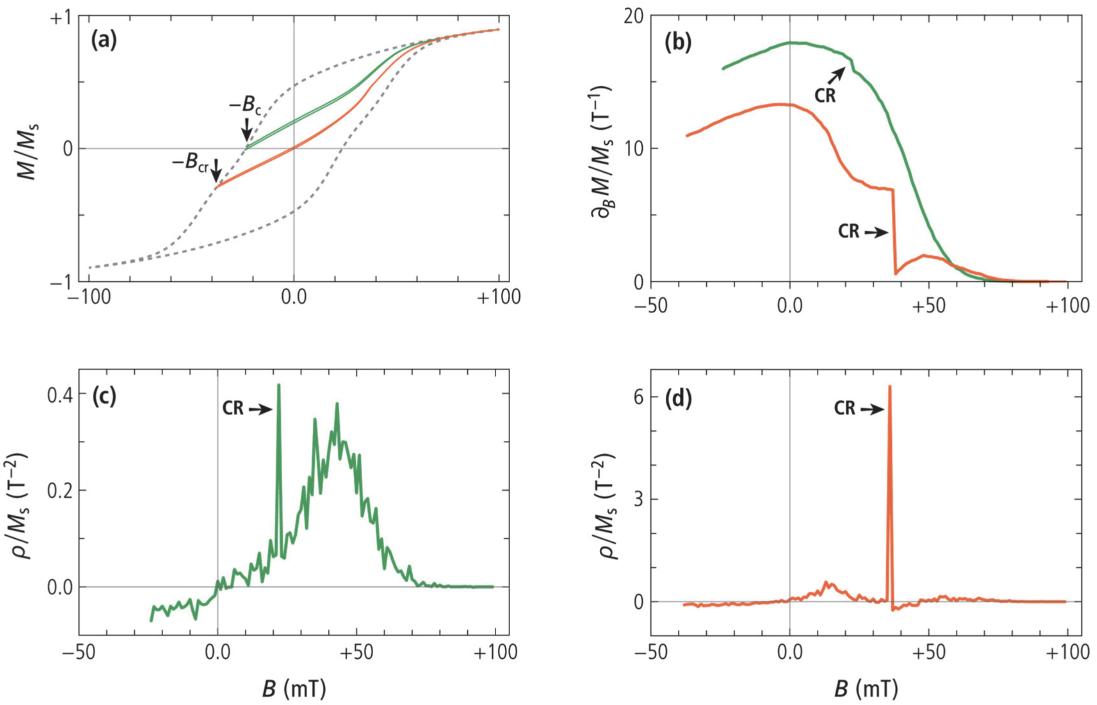

Figure S14: (**a**) Hysteresis (dashed lines) and two consecutive pairs of simulated FORCs starting at the coercive field B_c and at the coercivity of remanence B_{cr} (solid lines), for the ensemble of ~30,000 collapsed chains made of equant magnetosomes. (**b**) Derivative with respect to the applied field of one FORC of each pair in (a). Notice the discontinuities at $B = -B_r$, which correspond to the central ridge (CR) in the FORC diagram. (**c**) FORC function ρ defined by finite differences of the FORC pair starting at $B_r \approx -B_c$ in (a). The central ridge appears as a sharp peak at $B = -B_r$. (**d**) Same as (c) for the FORC pair starting at $B_r \approx -B_{cr}$. Irregular oscillations in (c-d) are produced by statistical noise associated with the limited number of simulated chains.

Table S2: Chain model parameters and their meaning.

**Parameter**	**Explanation**
$g_0, g_{\min}$	Mean and minimum gap between magnetosomes.
$\mathbf{H}$, $\mathbf{h}$	Applied magnetic field vector, and parallel unit vector.
H_c, $H_\mathrm{c}^\mathrm{max}$	Coercive field or horizontal FORC coordinate, and its maximum value
H_r, $H_\mathrm{r}^\mathrm{min}$, $H_\mathrm{r}^\mathrm{max}$	Reversal field, and corresponding minimum and maximum values.
H_sat	Saturation field.
H_u, $H_\mathrm{u}^\mathrm{min}$, $H_\mathrm{u}^\mathrm{max}$	Vertical FORC coordinate, and its minimum and maximum values
δH	Field step used in the FORC simulation
K_1, K_2	First and second cubic magnetocrystalline anisotropy constant.
l	Lag of sub-chain 2 with respect to sub-chain 1
n_t	Number of tapered magnetosomes at each chain end.
N, N_1, N_2	Number of magnetosomes in a single chain, or in the two chains composing a double chain.
s_0, s_t	Full magnetosome width (size of mature magnetosomes), and width of end magnetosomes in tapered chains.
$t(n)$	Chain tapering function, defining the size reduction of magnetosomes as a function of their position n inside the chain.
w_0	Mean chamfer width.
β_b	Chain bending angle.
θ, $\boldsymbol{\theta}$	Polar angle of magnetic moment, and vector of polar angles.
ϑ	Angle between midpoint chain axis and applied field.
$\varkappa_\mathrm{c}$	Concentration parameter for the Fisher-Von Mises distribution governing crystal axes misalignment with respect to shape axes.
$\varkappa_\mathrm{s}$	Concentration parameter for the Fisher-Von Mises distribution governing shape axes misalignment with respect to the chain axis.
λ_0	Mean magnetosome elongation.
μ_s	Spontaneous magnetization of magnetosomes.
σ_x	Standard deviation of the normal distribution governing the transversal randomization of magnetosome position.
σ_z	Standard deviation of the normal distribution governing the longitudinal (gap) randomization of magnetosome position.
σ_s	Standard deviation of the normal distribution governing the shape axes length randomization.
φ, $\boldsymbol{\varphi}$	Azimuthal angle of magnetic moment, and vector of azimuthal angles
ϕ	Azimuthal angle between field direction and chain plane.

Table S3: Other parameters and their meaning.

Parameter	Explanation
a_1, a_2, a_3	Lengths of shape axes, with a_3 nearly parallel to the chain axis.
A_{ex}	Exchange constant
$\mathbf{c}_1$, $\mathbf{c}_2$, $\mathbf{c}_3$	(100) magnetocrystalline axes of cubic anisotropy
g^l, g^t	Longitudinal and transversal gap between magnetosomes
$\mathbf{m}$	Magnetic moment vector.
s	Magnetosome width.
$t(n)$	Chain tapering function, defining the size reduction of magnetosomes as a function of their position n inside the chain.
$\mathbf{u}$	Unit vector specifying the magnetic moment direction.
w	Chamfer width.
V, V_0	Individual magnetosome volume, and mean volume of mature magnetosomes
l_{ex}	Exchange length
λ	Magnetosome elongation

References

Abo, G. S., Hong, Y.-K., Park,J., Lee, J., Lee, W., & Choi, B.-C (2013). Definition of magnetic exchange length. *IEEE Transactions on Magnetics*, *49*, 4937–4939, https://doi.org/10.1109/TMAG.2013.2258028.

Aragón, R. (1992). Cubic magnetic anisotropy of nonstoichiometric magnetite. *Physical Review B*, *46*, 5334–5338, https://doi.org/10.1103/PhysRevB.46.5334.

Barrett, M., Deschner, A., Embs, J. P., & Rheinstädter, M. C. (2011). Chain formation in a magnetic fluid under the influence of strong external magnetic fields studied by small angle neutron scattering. *Soft Matter*, *7*, 6678–6683, https://doi.org/10.1039/C1SM05104K.

Bazylinski, D. A., Frankel, R. B., Heywood, B. R., Mann, S., King, J. W., Donaghay, P. L., & Hanson, A. K. (1995). Controlled biomineralization of magnetite (Fe_3O_4) and greigite (Fe_3S_4) in a magnetotactic bacterium. *Applied and Environmental Microbiology*, *61*, 3232–3239, https://doi.org/10.1128/aem.61.9.3232-3239.1995.

Berndt, T. A., Chang, L., & Pei, Z. (2020). Mind the gap: Towards a biogenic magnetite palaeo-environmental proxy through an extensive finite-element micromagnetic simulation. *Earth and Planetary Science Letters*, *532*, 116010, https://doi.org/10.1016/j.epsl.2019.116010.

Blondeau, M., Guyodo, Y., Guyot, F., Gatel, C., Menguy, N., Chebbi, I., Haye, B., Durand-Dubiel, M., Alphandery, E., Brayner, R., & Coradin, T (2018). Magnetic-field induced rotation of magnetosome chains in silicified magnetotactic bacteria. *Scientific Reports*, *8*, 7699, https://doi.org/10.1038/s41598-018-25972-x.

Butler, R. F., & Banerjee, S. K. (1975). Theoretical single-domain grain size range in magnetite and titanomagnetite. *Journal of Geophysical Research*, *80*, 4049–4058, https://doi.org/10.1029/JB080i029p04049.

Chang, L., Harrison, R. J., & Berndt, T. A. (2019). Micromagnetic simulations of magnetofossils with realistic size and shape distributions: Linking magnetic proxies with nanoscale observations and implications for magnetofossil identification. *Earth and Planetary Science Letters*, *527*, 115790, https://doi.org/10.1016/j.epsl.2019.115790.

Chen, A. P., Egli, R., & Moskowitz, B. M. (2007). First-order reversal curve (FORC) diagrams of natural and cultured biogenic magnetic particles. *Journal of Geophysical Research: Solid Earth*, *112*, B08S90, https://doi.org/10.1029/2006JB004575.

Chen, D.-X., & Brug, J. A. (1991). Demagnetizing factors for cylinders. *IEEE Transactions on Magnetics*, *27*, 3601–3619, https://doi.org/10.1109/20.102932.

Clemett, S. J., Thomas-Keprta, K. L., Shimmin, J., Morphew, M., McIntosh, J. R., Bazylinski, D. A., Kirschvink, J. L., Wentworth, S. J., McKay, D. S., Vali, H., Gibson Jr., E. K., & Romanek, C. S. (2002). Crystal morphology of MV-1 magnetite. *American Mineralogist*, *87*, 1727–1730, https://doi.org/10.2138/am-2002-11-1223.

Draper, O., Byrne M. E., Li Z., Keyhani S., Barrozo J.C. Jensen G., & Komeili A. (2011). MamK, a bacterial actin, forms dynamic filaments in vivo that are regulated by the acidic proteins MamJ and LimJ. *Molecular Microbiology*, *82*, 342–354, https://doi.org/10.1111/j.1365-2958.2011.07815.x.

Egli, R. (2013). VARIFORC: An optimized protocol for calculating non-regular first-order reversal curve (FORC) diagrams. Global and Planetary Change, 110, 302–320, https://doi.org/10.1016/j.gloplacha.2013.08.003.

Enkin, R. J., & Williams, W. (1994). Three-dimensional micromagnetic analysis of stability in fine magnetic grains. *Journal of Geophysical Research*, *99*, 611–618, https://doi.org/10.1029/93JB02637.

Fabian, K., & Heider, F. (1996). How to include magnetostriction in micromagnetic models of titanomagnetite grains. *Geophysical Research Letters*, *23*, 2839–2842, https://doi.org/10.1029/96GL01429.

Fabian, K., & Shcherbakov, V. (2018). Energy barriers in three-dimensional micro-magnetic models and the physics of thermoviscous magnetization, *Geophysical Journal International*, *215*, 314–324, https://doi.org/10.1093/gji/ggy285.

Faivre, D., & Schüler, D. (2008). Magnetotactic bacteria and magnetosomes. *Chemical Reviews*, *108*, 4875–4898, https://doi.org/10.1021/cr078258w.

Fukuma, K., & Dunlop, D. J. (2006). Three-dimensional micromagnetic modeling of randomly oriented magnetite grains. *Journal of Geophysical Research: Solid Earth*, *111*, B12S11, https://doi.org/10.1029/2006JB004562.

Galindo-González, C., Feinberg, J. M., Kasama, T., Cervera Gontard, L., Pósfai, M., Kósa, I., Duran, J. D. G., Gil, J. E., Harrison, R. J., & Dunin-Borkowski, R. E. (2009). Magnetic and microscopic characterization of magnetite nanoparticles adhered to clay surfaces. *American Mineralogist*, *94*, 1120–1129, https://doi.org/10.2138/am.2009.3167.

Gorby, Y. A., Beveridge, T. J., & Blakemore, R. P. (1988). Characterization of the bacterial magnetosome membrane. *Journal of Bacteriology*, *170*, 834–841, https://doi.org/10.1128/jb.170.2.834-841.1988.

Hanzlik, M., Winklhofer, M., & Petersen, N. (2002). Pulsed-field-remanence measurements on individual magnetotactic bacteria. *Journal of Magnetism and Magnetic Materials*, *248*, 258–267, https://doi.org/10.1016/S0304-8853(02)00353-0.

Hesse, P. (1994). Evidence for bacterial palaeoecological origin of mineral magnetic cycles in oxic an sub-oxic Tasman Sea sediments. *Marine Geology*, *117*, 1–17, https://doi.org/10.1016/0025-3227(94)90003-5.

Iida, A., & Akai, J. (1996). TEM study on magnetotactic bacteria and contained magnetite grains as biogenic minerals, mainly from Hokuriku-Nigata region, Japan. *Scientific Reports of the Niigata University, Series EW, Geology and Mineralogy*, *11*, 43–66, https://ci.nii.ac.jp/naid/120006761887/en/.

Isambert, A., Menguy, M., Larquet, E., Guyot, F., & Valet, J.-P. (2007). Transmission electron microscopy study of magnetites in a freshwater population of magnetotactic bacteria. *American Mineralogist*, *92*, 621–630, https://doi.org/10.2138/am.2007.2278.

Jacobs, I. S., & Bean, C. P. (1955). An approach to elongated fine-particle magnetism, *Physical Review*, *100*, 1060–1067, https://doi.org/10.1103/PhysRev.100.1060.

Joseph, R. I. (1966). Ballistic demagnetizing factor in uniformly magnetized cylinders. *Journal of Applied Physics*, *37*, 4639–4643, https://doi.org/10.1063/1.1708110.

Kakol, Z., & Honig, J. M. (1989). Influence of deviations from ideal stoichiometry on the anisotropy parameters of magnetite $Fe_{3(1-\delta)}O_4$. *Physical Review B*, *40*, 9090–9097, https://doi.org/10.1103/PhysRevB.40.9090.

Kalirai, S. S., Bazylinski, D. A., & Hitchcock, A. P. (2013). Anomalous magnetic orientation of magnetosome chains in a magnetotactic bacterium: Magnetovibrio blakemorei Strain MV-1. *PLoS ONE*, *8*, e53368, https://doi.org/10.1371/journal.pone.0053368.g003.

Katzmann, E., Eibauer, M., Lin, W., Pan, Y., Plitzko, J. M., & Schüler, D. (2013). Analysis of magnetosome chains in magnetotactic bacteria by magnetic measurements and automated image analysis of electron micrograph. *Applied and Environmental Microbiology*, *79*, 7755–7762, https://doi.org/10.1128/AEM.02143-13.

Kiani, B., Faivre, D., & Klumpp, S. (2015). Elastic properties of magnetosome chains. *New Journal of Physics*, *17*, 043007, https://doi.org/10.1088/1367-2630/17/4/043007.

Kiani, B., Faivre, D., & Klumpp, S. (2018). Self-organization and stability of magnetosome chains – A simulation study. PLoS ONE, 13, e0190265, https://doi.org/10.1371/journal.pone.0190265.

Kobayashi, A., Kirschvink, J. L., Nash, C. Z., Kopp, R. E., Sauer, D. A., Bertani, L. E., Voorhout, W. F., & Taguchi, T. (2006). Experimental observation of magnetosome chain collapse in magnetotactic bacteria: Sedimentological, paleomagnetic, and evolutionary implications. *Earth and Planetary Science Letters*, *245*, 538–550, https://doi.org/10.1016/j.epsl.2006.03.041.

Komeili, A., Li Z., Newman D.K., & Jensen G.J. (2006). Magnetosomes are cell membrane invaginations organized by the actin-like protein MamK. *Science*, *311*, 242–245, https://doi.org/10.1126/science.1123231.

Körnig, A., Winklhofer, M., Baumgartner, J., Gonzalez, T. P., Fratzl, P., & Faivre, D. (2014). Magnetite crystal orientation in magnetosome chains. *Advanced Functional Materials*, *24*, 3926–3932, https://doi.org/10.1002/adfm.201303737.

Lang, C., & Schüler, D. (2006). Biogenic nanoparticles: production, characterization, and application of bacterial magnetosomes. *Journal of Physics: Condensed Matter*, *18*, S2815–S2828, https://doi.org/10.1088/0953-8984/18/38/S19.

Lean, C. M. B., & McCave, I. N. (1998). Glacial to interglacial magnetic and palaeoceanographic changes at Chatham Rise, SW Pacific Ocean. *Earth and Planetary Science Letters*, *163*, 247–260, https://doi.org/10.1016/S0012-821X(98)00191-5.

Lefèvre, C. T., Pósfai, M., Abreu, F., Lins, U., Frankel, & R. B., & Bazylinski, D. A. (2011). Morphological features of elongated-anisotropic magnetosome crystals in magnetotactic bacteria of the Nitrospirae phylum and the Deltaproteobacteria class. *Earth and Planetary Science Letters*, *312*, 194–200, https://doi.org/10.1016/j.epsl.2011.10.003.

Li, J., Wu W., Liu Q., & Pan Y. (2012). Magnetic anisotropy, magnetostatic interactions and identification of magnetofossils. Geochemistry Geophysics Geosystems, 13, Q10Z51, https://doi.org/10.1029/2012GC004384.

Lins, U., & Farina, M. (2004). Magnetosome chain arrangement and stability in magnetotactic cocci. *Antoine van Leeuwenhoek*, *85*, 335–341, https://doi.org/10.1023/B:ANTO.0000020393.71843.b0.

Lippert, P. C., & Zachos, J. C. (2007). A biogenic origin for anomalous fine-grained magnetic material at the Paleocene-Eocene boundary at Wilson Lake, New Jersey. *Paleoceanography*, *22*, PA4104, https://doi.org/10.1029/2007PA001471.

Ludwig, P., Egli R., Bishop S., Chernenko V., Frederichs T., Rugel G., Merchel S., & Orgeira M.J. (2013). Characterization of primary and secondary magnetite in marine sediment by combining chemical and magnetic unmixing techniques. Global and Planetary Change, 110, 321–339, https://doi.org/10.1016/j.gloplacha.2013.08.018.

Mann, S., Frankel, R. B., & Blakemore, R. P. (1984). Structure, morphology and crystal growth of bacterial magnetite. *Nature*, *310*, 405–407, https://doi.org/10.1038/310405a0.

Mann, S., Sparks, N. H. C., & Blakemore, R. P. (1987). Structure, morphology and crystal growth of anisotropic magnetite crystals in magnetotactic bacteria. *Proceedings of the Royal Society of London Series B, Biological Sciences*, *231*, 477–487, https://doi.org/10.1098/rspb.1987.0056.

McNeill, D. F. (1990). Biogenic magnetite from surface Holocene carbonate sediments, Great Bahama Bank. *Journal of Geophysical Research*, *95*, 4363–4371, https://doi.org/10.1029/JB095iB04p04363.

Meldrum, F. C., Mann, S., Heywood, B. R., Frankel, R. B., Bazylinski, D. A. (1993). Electron microscopy study of magnetosomes in a cultured coccoid magnetotactic bacterium. *Proceedings of the Royal Society of London B*, *251*, 231–236, https://doi.org/10.1098/rspb.1993.0034.

Meldrum, F. C., Mann, S., Heywood, B. R., Frankel, R. B., Bazylinski, D. A. (1993). Electron microscopy study of magnetosomes in two cultured vibroid magnetotactic bacteria. *Proceedings of the Royal Society of London B*, *251*, 237–242, https://doi.org/10.1098/rspb.1993.0035.

Mickoleit, F., Borkner, C. B., Toro-Nahuelpan, M., Herold, H. M., Maier, D. S., Pitzko, J. M., Scheibel, T., & Schüler, D. (2018). In vivo coating of bacterial magnetic nanoparticles by magnetosome expression of spider silk-inspired peptides. *Biomacromolecules*, *19*, 962–972, https://doi.org/10.1021/acs.biomac.7b01749.

Moisescu, C., Bonneville, S., Tobler, D., Ardelean, I., & Benning, L. G. (2008). Controlled biomineralization of magnetite (Fe_3O_4) by Magnetospirillum gryphiswaldense. *Mineralogical Magazine*, *72*, 333–336, https://doi.org/10.1180/minmag.2008.072.1.333.

Monteil, C. L., Perrière, G., Menguy, N., Ginet, N., Alonso, B., Waisbord, N., Cruveiller, S., Pignol, D., & Lefèvre, C. T (2018). Genomic study of a novel magnetotactic Alphaproteobacteria uncovers the multiple ancestry of magnetotaxis. *Environmental Microbiology*, *20*, 4415–4430, https://doi.org/10.1111/1462-2920.14364.

Murat, D., Quinlan A., Vali H., & Komeili A. (2010). Comprehensive genetic dissection of the magnetosome gene island reveals the step-wise assembly of a prokaryotic organelle. *Proceedings of the Royal Academy of Sciences of the United States*, *107*, 5593–5598, https://doi.org/10.1073/pnas.0914439107.

Muxworthy, A. R., & Williams, W. (2006). Critical single-domain/multidomain grain sizes in noninteracting and interacting elongated magnetite particles: Implications for magnetosomes. *Journal of Geophysical Research*, *111*, B12S12, https://doi.org/10.1029/2006JB004588.

Muxworthy, A. M., Williams, W., Virdee, D. (2003). Effect of magnetostatic interactions on the hysteresis parameters of single-domain and pseudo-single-domain grains. *Journal of Geophysical Research*, *108*, 2517, https://doi.org/10.1029/2003JB002588.

Newell, A. J. (2009). Transition to superparamagnetism in chains of magnetosome crystals. *Geochemistry Geophysics Geosystems*, *10*, Q11Z08, https://doi.org/10.1029/2009GC002538.

Newell, A. J., & Merrill, R. T. (2000). Nucleation and stability of ferromagnetic states. *Journal of Geophysical Research*, *105*, 19377–19391, https://doi.org/10.1029/2000JB900121.

Orue, I., Marcano, L., Bender, P., García-Prieto, A., Valencia, S., Mawass, M. A., Gil-Cartón, D., Alba Venero, D., Honecker, D., García-Arribas, A., Fernández Barquín, L., Muela, A., & Fdez-Gubieda, M. L (2018). Configuration of the magnetosome chain: a natural magnetic nanoarchitecture. *Nanoscale*, *10*, 7407–7419, https://doi.org/10.1039/C7NR08493E.

Osborn, J. A. (1945). Demagnetizing factors of the general ellipsoid. *Physical Review*, *67*, 351–357, https://doi.org/10.1103/PhysRev.67.351.

Petersen, N., von Dobeneck, T., & Vali, H. (1986). Fossil bacterial magnetite in deep-sea sediments from the South Atlantic Ocean. *Nature*, *320*, 611–615, https://doi.org/10.1038/320611a0.

Petersen, N., Weiss, D. G., & Vali, H. (1989). Magnetic bacteria in lake sediments. In: Lowes F. J., Collinson, D.W., Parry, J. H., Runcorn, S. K., Tozer, D. C., & Soward, A. (eds) Geomagnetism and Paleomagnetism, *NATO ASI Series (Series C: Mathematical and Physical Sciences)*, *261*, 231–241, Springer, Dordrecht. https://doi.org/10.1007/978-94-009-0905-2_17.

Pike, C. R., Roberts, A. P., & Verosub, K. L. (1999). Characterizing interactions in fine magnetic particle systems using first order reversal curves. *Journal of Applied Physics*, *85*, 6660–6667, https://doi.org/10.1063/1.370176.

Pósfai, M., Kasama, T., & Dunin-Borkowski, R. E. (2006). Characterization of bacterial magnetic nanostructures using high-resolution transmission electron microscopy and off-axis electron holography. In: Schüler, D. (eds) Magnetoreception and magnetosomes in bacteria, *Microbiology Monographs*, *3*, 197–225, https://doi.org/10.1007/7171_044.

Pósfai, M., Lefevre, C., Trubitsyn, D., Bazylinski, D., & Frankel, R. (2013). Phylogenetic significance of composition and crystal morphology of magnetosome minerals. *Frontiers in Microbiology*, *4*, 1–15, https://doi.org/10.3389/fmicb.2013.00344.

Ruder, W. C., Hsu C.-P. D., Edelman, B. D., Schwartz, R., & LeDuc, P. R. (2012). Biological colloid engineering: self-assembly of dipolar ferromagnetic chains in a functionalized biogenic ferrofluid. *Applied Physics Letters*, *101*, 063701, https://doi.org/10.1063/1.4742329.

Shcherbakov, V. P., Winklhofer, M., Hanzlik, M., & Petersen, N. (1997). Elastic stability of chains of magnetosomes in magnetotactic bacteria. *European Biophysics Journal*, *26*, 319–326, https://doi.org/10.1007/s002490050086.

Simpson, E. T., Kasama, T., Pósfai, M., Busek, P. R., Harrison, R. J., & Dunin-Borkowski, R. E. (2005). Magnetic induction mapping of magnetite chains in magnetotactic bacteria at room temperature and close to the Verwey transition using electron holography. *Journal of Physics: Conference Series*, *17*, 108–121, https://doi.org/10.1088/1742-6596/17/1/017.

Sparks, N. H. C., Mann, S., Bazylinski, D. A., Lovely, D. R., Jannash, H. W., & Frankel, R. B. (1990). Structure and morphology of magnetite anaerobically-produced by a marine magnetotactic bacterium and a dissimilatory iron-reducing bacterium. Earth and Planetary Science Letters, 98, 14–22, https://doi.org/10.1016/0012-821X(90)90084-B.

Spring, S., Amann, R., Ludwig, W., Schleifer, K.-H., Schüler, D., Poralla, K., & Petersen, N. (1994). Phylogenetic analysis of uncultured magnetotactic bacteria from the alpha-subclass of Proteobacteria. Systematic and Applied Microbiology, 17, 501–508, https://doi.org/10.1016/S0723-2020(11)80068-8.

Stoner, E. C., & Wohlfarth, E. P. (1948). A mechanism of magnetic hysteresis in heterogeneous alloys. *Philosophical Transactions of the Royal Society of London A*, *240*, 599–642, https://doi.org/10.1098/rsta.1948.0007.

Tombácz, E., Csanaky, & C., Illés, E. (2001). Polydisperse fractal aggregate formation in clay mineral and iron oxide suspensions, pH and ionic strength dependence. *Colloid and Polymer Science*, *279*, 484–492, https://doi.org/10.1007/s003960100480.

Vali, H., Förster, O. Amarantidis, G., & Petersen, N. (1987). Magnetotactic bacteria and their magnetofossils in sediments. *Earth and Planetary Science Letters*, *86*, 389–400, https://doi.org/10.1016/0012-821X(87)90235-4.

Varón, M., Beleggia, M., Kasama, T., Harrison, R. J., Dunin-Borkowski, R. E., Puntes, V. F., & Frandsen, C. (2013). Dipolar magnetism in ordered and disordered low-dimensional nanoparticle assemblies. *Scientific Reports*, *3*, 1234, https://doi.org/10.1038/srep01234.

Wang, M., He, L., & Yin, Y. (2013). Magnetic field guided colloidal assembly. *Materials Today*, *16*, 110–116, https://doi.org/10.1016/j.mattod.2013.04.008.

Werckmann, J., Cypriano, J., Lefèvre, C. T., Dembelé, K., Ersen, O., Bazylinski, D. A., Lins, U., & Farina, M. (2017). Localized iron accumulation precedes nucleation and growth of magnetite crystals in magnetotactic bacteria. Scientific Reports, 7, 8291, https://doi.org/10.1038/s41598-017-08994-9.

Weyland, M., Yates, T. J. V., Dunin-Borkowski, R. E., Laffont, L., & Midgley, P. A. (2006). Nanoscale analysis of three-dimensional structures by electron tomography. *Scripta Materialia*, *55*, 29–33, https://doi.org/10.1016/j.scriptamat.2005.12.058.

Zhang, H., Menguy N., Wang F., Benzerara K., Leroy E., Liu P., Liu W., Wang C., Pan Y., Chen Z., & Li J. (2017). Magnetotactic coccus strain SHHC-1 affiliated to Alphaproteobacteria forms octahedral magnetite magnetosomes. *Frontiers in Microbiology*, *8*, 969, https://doi.org/10.3389/fmicb.2017.00969.

Zhang, Y., Shi, R., Xiong, H. Q., & Zhai, Y. (2005). One-dimensional magnetite chains of nanoparticles synthesis by self-assembly in magnetic field. *International Journal of Modern Physics B*, *19*, 2757–2762, https://doi.org/10.1142/S0217979205031651.

Zhu, X., Hitchcock, A. P., Bazylinski, D. A., Denes, P., Joseph, J., Lins, U., Marchesini, S., Shiu, H.-W., Tyliszczak, T., & Shapiro, D. A. (2016). Measuring spectroscopy and magnetism of extracted and intracellular magnetosomes using soft X-ray ptychography. *Proceedings of the National Academy of Sciences of the United States*, *113*, E8219–E8227, https://doi.org/10.1073/pnas.1610260114.
