# Supplementary figures and images for: Key Signatures of Magnetofossils Elucidated by Mutant Magnetotactic Bacteria and Micromagnetic Calculations
Source: J Geophys Res Solid Earth. 2022 Jan 18;127(1):e2021JB023239. doi: 10.1029/2021JB023239 (PMC9017866; doi:10.1029/2021JB023239)

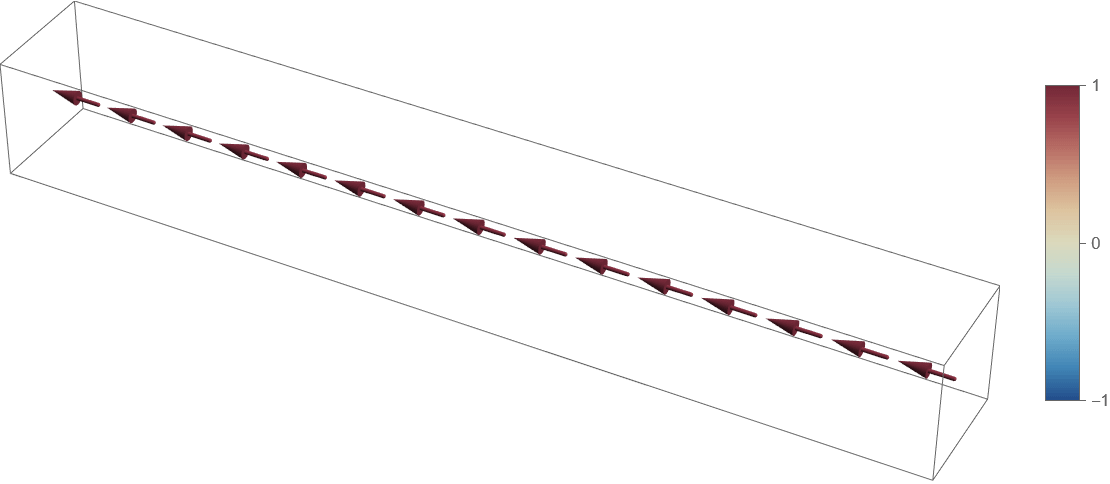

Supplement: Supplementary file 2 — Movie S1 [file JGRB-127-0-s001.gif]

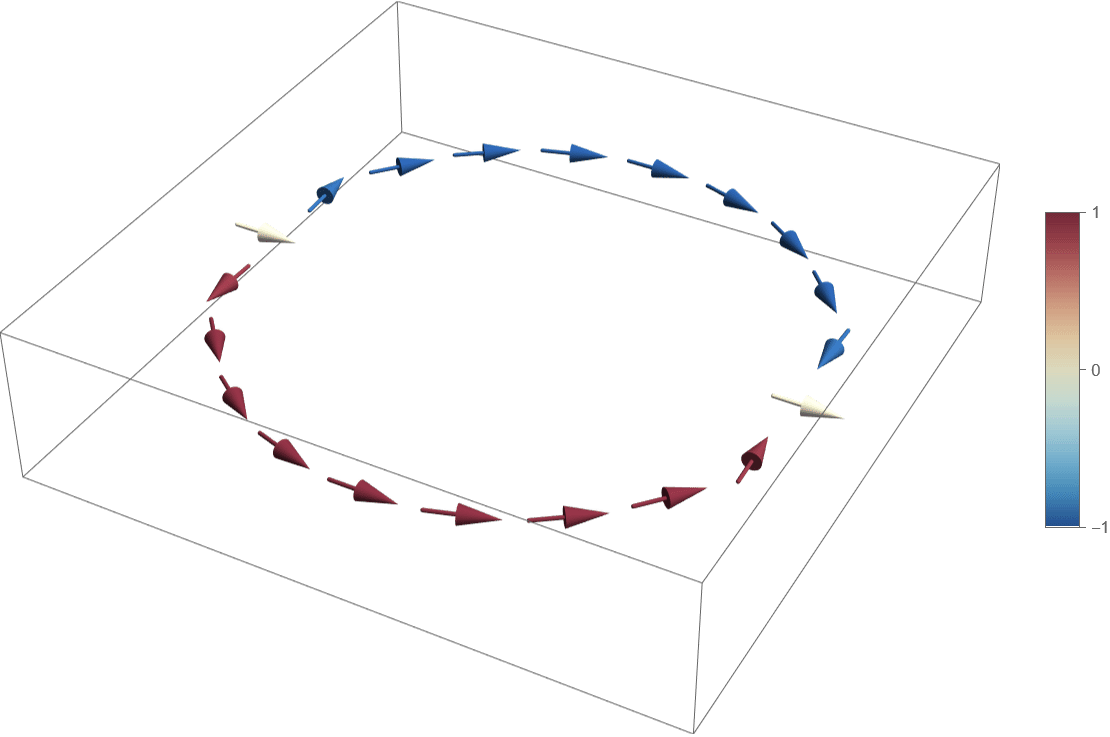

Supplement: Supplementary file 3 — Movie S2 [file JGRB-127-0-s003.gif]

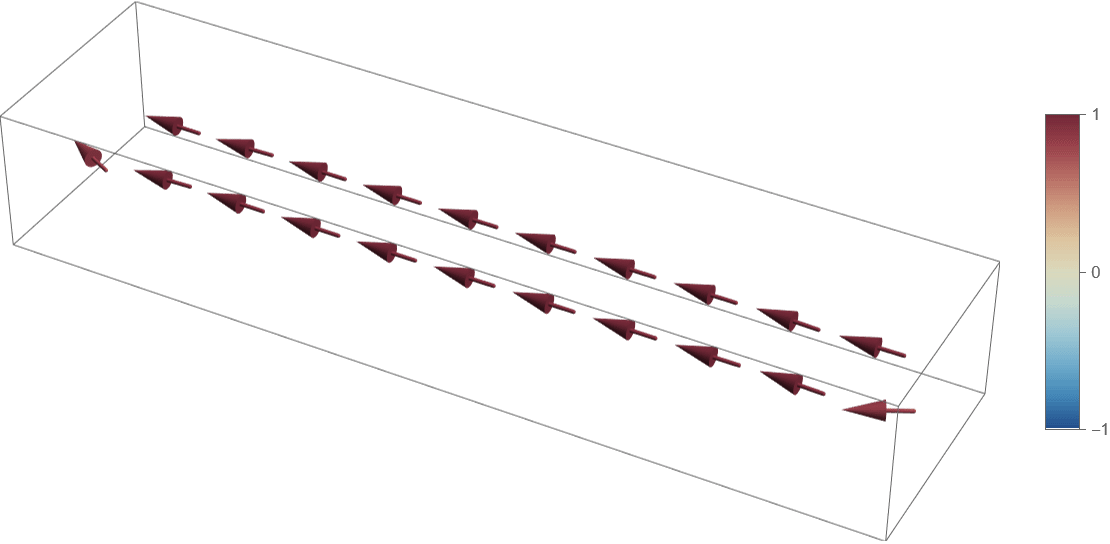

Supplement: Supplementary file 4 — Movie S3 [file JGRB-127-0-s002.gif]

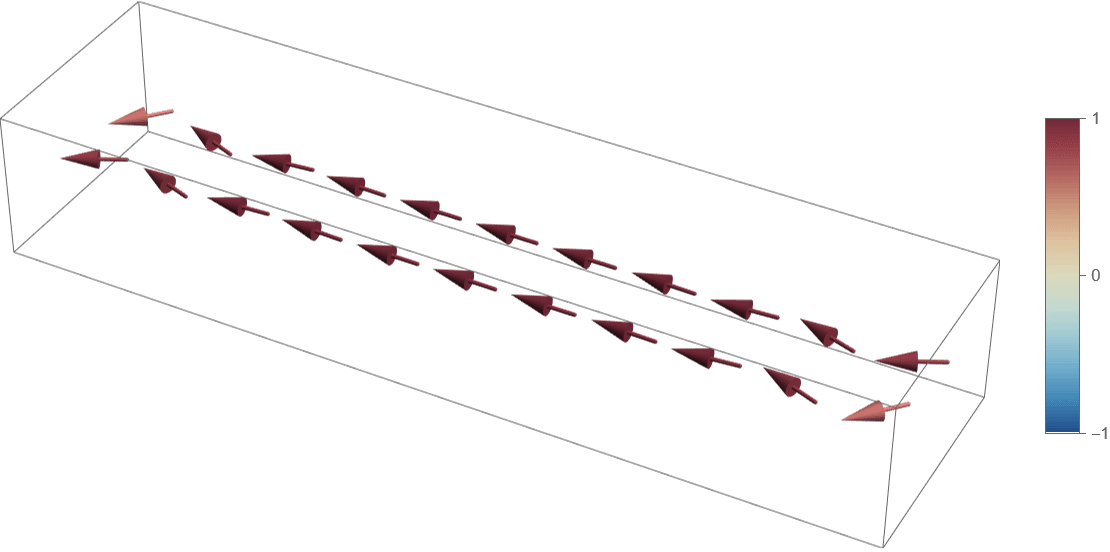

Supplement: Supplementary file 5 — Movie S4 [file JGRB-127-0-s005.gif]
